# Targeted therapy combinations with ipatasertib in multi-cell type 3D tumor spheroid models

**DOI:** 10.20935/acadonco7726

**Published:** 2025-06-17

**Authors:** Beverly A. Teicher, Naoko Takebe, Thomas S. Dexheimer, Thomas E. Silvers, Nathan P. Coussens, Melinda G. Hollingshead, James H. Doroshow

**Affiliations:** 1 Division of Cancer Treatment and Diagnosis, National Cancer Institute, Bethesda, MD 20892, USA.; 2 Molecular Pharmacology Laboratory, Applied and Developmental Research Directorate, Frederick National Laboratory for Cancer Research, Frederick, MD 21702, USA.

**Keywords:** ipatasertib, spheroids, patient-derived cell lines, AKT inhibition, selumetinib, ravoxertinib

## Abstract

We investigated the growth-inhibitory activity of the pan-AKT inhibitor ipatasertib in combination with other targeted therapies. Thirty-nine patient-derived cancer cell lines from the NCI Patient-Derived Models Repository and nine NCI-60 tumor cell lines were grown as mct-spheroids. The mct-spheroids, a mixture of tumor cells (60%), endothelial cells (25%), and mesenchymal stem cells (15%), were established for 3 days before compounds(s) were added. All agents were tested at concentrations up to the reported clinical C_max_ values or a high concentration of 10 μM. Cell viability was assayed using CellTiter-Glo 3D after 7 days of exposure. Ipatasertib was selective for tumor cells harboring activating PI3K/AKT/mTOR pathway mutations. Dual inhibition of the PI3K/AKT/mTOR and RAS/MEK/ERK pathways was very effective. The combination of ipatasertib with the MEK inhibitor selumetinib or the ERK inhibitor ravoxertinib resulted in additive and/or greater-than-additive cytotoxicity in approximately half the cell lines screened. The V600E mutation-specific BRAF inhibitor vemurafenib and the KRAS G12C selective inhibitor sotorasib in combination with ipatasertib were active in the eight BRAF V600E and four KRAS G12C mutant-containing cell lines, respectively. Vertical inhibition of the PI3K/AKT/mTOR pathway with the mTORC1/2 kinase inhibitor sapanisertib demonstrated additive and/or greater-than-additive effects in multiple cell lines. In early experiments, there was a correlation between the response to ipatasertib and selumetinib in two patient-derived tumor lines grown as mct-spheroids and the corresponding patient-derived xenografts. All data are accessible via the PubChem BioAssay public database.

## 1. Introduction

Transmembrane receptors, especially receptors with tyrosine kinase activity, often initiate intracellular signaling through RAS. Two major cytosolic pathways downstream of RAS are (1) the v-Raf murine sarcoma viral oncogene homolog B (RAF)/mitogen-activated protein kinase (MEK)/extracellular signal-regulated kinase (ERK) pathway and (2) the phosphoinositide 3-kinase (PI3K)/v-akt murine thymoma viral oncogene (AKT)/mammalian target of rapamycin (mTOR) pathway [1]. These kinases and phosphatases are targets for medicinal chemistry, and all have small-molecule targeted inhibitors, now approved drugs. Protein kinases have pivotal regulatory roles in nearly every aspect of cell biology, including cell growth and proliferation, cell survival, differentiation, the immune response, metabolism, and transcription [2]. The clinical activity of the RAS G12C-targeted drugs adagrasib and sotorasib advanced the treatment of non-small-cell lung cancer. These drugs have limitations in the depth and duration of the response but have validated the deep interest of the cancer community in KRAS as a drug target [3–6]. AKT is the most frequently upregulated protein kinase in human cancers. AKT activation is potentiated by PI3K and inhibited by phosphatase and tensin homolog (PTEN). Aberrant activation of the PI3K/AKT pathway results in resistance to anticancer therapies in many human cancers. Ipatasertib, an orally administered ATP-competitive selective AKT inhibitor, was explored as a treatment for prostate cancer and breast cancer [7, 8]. Phosphoinositide 3-kinases (PI3Ks) are lipid kinase proteins with a regulatory and a catalytic subunit that phosphorylate the 3-hydroxyl group of inositol phospholipids. Copanlisib is a class I PI3K inhibitor active against PI3K-δ and PI3K-α isoforms. In in vitro studies with leukemia cells, the copanlisib concentrations leading to a half-maximal reduction in leukemia cell survival were more than 10-fold lower for copanlisib than for idelalisib and duvelisib [9].

The mitogen-activated protein kinase (MAPK) pathway, including the kinases RAS, RAF, MEK, and ERK, is essential for normal cellular function and is implicated in a broad array of cancers. The MAPK pathway transduces extracellular signals through receptor tyrosine kinase activity [10]. The MAPK pathway is upregulated in 66% of melanomas, and many harbor mutated BRAF with the activating BRAF V600E mutation; thus, targeting the RAS/RAF/mitogen-activated protein (MAP)/extracellular signal-regulated kinase (ERK) kinase (MEK)/ERK pathway may be effective in melanoma. The V600E mutation results in an acidic substitution in the kinase domain of BRAF, thus increasing kinase activity compared with wildtype BRAF [11]. Inhibition of MEK by the MEK inhibitor selumetinib is cytostatic as a monotherapy in melanoma but cytotoxic when combined with docetaxel [12–14]. Vemurafenib, dabrafenib, and encorafenib inhibit BRAF kinase and interfere with the mitogen-activated protein kinase (MAPK) signaling pathway, which regulates the proliferation and survival of melanoma cells [15–17]. MEK inhibitors have been combined with BRAF inhibitors to further reduce MAPK signaling.

ERK1 and ERK2 are the protein kinases downstream of MEK in the RAS/RAF/MEK signaling pathway. MEK1/2 activate human ERK1/2 by catalyzing the phosphorylation of two amino acids, both of which are in the enzyme activation region and are required for enzyme activation. ERK1/2 phosphorylates a range of cytoplasmic and nuclear substrates. The linear MAP kinase pathway branches extensively at the ERK1/2 node [2]. AKT is a family of highly conserved homologues, AKT1, AKT2, and AKT3, found in the cytoplasm of human cells. Following activation, AKT is found at the plasma membrane and in the nucleus [18].

Ipatasertib is a novel, highly selective ATP-competitive AKT inhibitor with selectivity, efficacy, and oral bioavailability that support clinical development as an anticancer agent either singly or in combination with chemotherapeutic agents [19]. Treatment with ipatasertib improved progress-free survival in a subset of metastatic TNBC patients when combined with paclitaxel in the first-line setting, indicating that PI3K pathway-targeted therapies may be promising [20]. The mammalian target of rapamycin (mTOR) is an atypical serine/threonine protein kinase that acts as a central hub of signal integration. mTOR is found in two large multiprotein complexes referred to as mTORC1 and mTORC2. mTORC1 controls the anabolic pathways needed for cell growth, proliferation, and survival, including protein synthesis, ribosome production, lipid synthesis, and nucleotide synthesis [21]. Sapanisertib, a dual mTORC1/2 kinase inhibitor, was granted fast track designation by the FDA for patients with unresectable or metastatic squamous-cell non-small-cell lung cancer who have an NRF2 mutation. The mTORC1 phosphatase and tensin homolog (PTEN) acts as an upstream regulator to the PI3K/AKT/mTOR pathway [22].

The current study was undertaken to explore the activity of the AKT inhibitor ipatasertib in combination with other targeted agents covering the pathways downstream of RAS in an mct-spheroid model developed from patient-derived cell lines in the NCI Patient-Derived Models Repository (https://pdmr.cancer.gov/ [cited 2025 May 10]) and established cell lines from the NCI60 tumor cell line panel covering breast, colon, pancreas, prostate, ovary, lung, and head and neck cancers and melanoma. All data are accessible via the PubChem BioAssay public database (Supplemental Table S1).

## 2. Materials and methods

### Drugs and Investigational Agents:

The drugs and investigational agents used in this study are ipatasertib (NSC767898), selumetinib (NSC741078), trametinib (758246), adavosertib (NSC754352), everolimus (NSC733504), sapanisertib (NSC764658), vemurafenib (NSC761431), venetoclax (NSC766270), copanlisib (NSC809693), apelisib (NSC765974), palbociblib (NSC758247), vemurafenib (NSC761431), dabrafenib (NSC764134), ravoxertinib (NSC781343), BMS-387032 (NSC767048), sotorasib (NSC818433), talazoparib (NSC767125), and afursertib (NSC778305), as listed in Table 1. All the agents used, including staurosporine (NSC755774) and gemcitabine (NSC613327), were obtained from the Developmental Therapeutics Program, NCI. FDA-approved anticancer drugs are available from the NCI at http://dtp.nci.nih.gov/branches/dscb/oncology_drugset_explanation.html [cited 2025 May 10]. Drugs and investigational agents were >95% pure by proton nuclear magnetic resonance (NMR) and liquid chromatography/mass spectrometry (LC/MS). Stock solutions were prepared at 400× in DMSO, at 800-fold the tested concentration, and stored at −80 °C prior to their use [23]. All drugs and investigational agents were tested over a range starting from a high concentration at or near the clinical Cmax and decreasing in half-log increments. If the clinical Cmax for an agent had not been determined, the highest concentration tested was 10 μM. The final concentration of DMSO in cell culture wells never exceeded 0.25% (*v*/*v*).

### NCI60 Cell Culture:

NCI60 cell lines were obtained from the NCI Developmental Therapeutics Program Tumor Repository. For each lot of cells, the Repository performed Applied Biosystems AmpFLSTR Identifiler testing with PCR amplification to confirm consistency with the published Identifier STR profile for each cell line. Cells were kept in continuous culture for no more than 20 passages. The optimal seeding densities for each of the cell lines at each time point assessed were determined prior to performing the concentration response studies. Briefly, the NCI60 human tumor lines were grown as monolayers in RPMI 1640 medium supplemented with 10% FBS and 2 mmol/L L-glutamine.

### MCT-Spheroid Cell Culture:

All cells were maintained in an incubator at 37 °C and 5% CO_2_ with 95% humidity. The PDC lines were cultured according to standard operating procedures established by the NCI PDMR (https://pdmr.cancer.gov [cited 2025 May 10]). Table 2 lists the cell lines, along with the number of the three cell types plated to form the mct-spheroids and the media used for tumor cell growth. The activities of single agents and combinations were evaluated in mct-spheroids grown from patient-derived tumor cells from the NCI Patient-Derived Models Repository (https://pdmr.cancer.gov [cited 2025 May 10]), including 171881-019-R-J1 invasive breast carcinoma; 349418-098-R, LG0567-F671, LG0703-F948, and 989133-093-R-J1 non-small-cell lung carcinomas; 417821-307-R-J1 lung squamous-cell carcinoma; 541946-237-B-J1 small-cell lung carcinoma; 186277-243-T-J2, 254851-301-R-J1, 276233-004-R-J1, 282377-053-R-J1, 361931-004-R-J1, 519858-162-T-J1, 616215-338-R-J1, 817829-284-R-J1, 825966-067-R-J1, CN0375-F725, 762968-020-R-J2, 439559-082-T-J2, and 463943-066-T-J2 colon carcinomas; 253994-281-T-J1 and 931267-113-T-J1 colorectal carcinomas; 292921-168-R-J2, 323965-272-R-J2, 521955-158-R3-J5, 521955-158-R6-J3, 521955-158-R7-J2, 885724-159-R-J1, 485368-065-R4-J2, and K24384-001-R pancreatic carcinomas; 556581-035-R-J1 ovarian carcinoma; 327498-153-R-J2 uterine carcinosarcoma; 138582-337-R-J1 Merkel cell tumor; 832693-133-R-J1, 845751-090-R-J2, and 958767-090-R-J1 head and neck squamous carcinomas; and 128128-338-R-J1, 156681-154-R-J1, and 299254-011-R-J1 melanomas. An additional 9 lines were from the NCI60 cell line panel, including MDA-MB-231 breast carcinoma, MDA-MB-468 breast carcinoma, MCF7 breast carcinoma, Hs578T breast carcinoma, MALME-3M melanoma, SK-MEL-2 melanoma, SK-OV-3 ovarian carcinoma, DU-145 prostate carcinoma, and PC-3 prostate carcinoma. All cell lines along with major genetic alterations are listed in Table 2. Pooled donor human umbilical vein endothelial cells (HUVECs, Lonza, cat. CC-2519) and human mesenchymal stem cells (hMSCs, Lonza, cat. PT-2501) were purchased from Lonza (Walkersville, MD, USA).

PDMR cell lines were grown in complete medium (DC medium): Dulbecco’s modified Eagle’s medium (DMEM)/F12 supplemented with 5% fetal bovine serum (FBS), 2 mM L-glutamine, 100 U/mL penicillin and 100 μg/mL streptomycin, 0.4 μg/mL hydrocortisone, 10 ng/mL epidermal growth factor (EGF) recombinant human protein, 24 μg/mL adenine, and 0.1 mg/mL primocin. During culture from frozen stock vials, PDMR cells were cultured in the same medium containing 10 μM Y-27632 (see Table 3). The PDCs were cultured in complete DMEM/F12 media without 10 μM Y-27632 dihydrochloride for at least two passages prior to the screen. The pooled donor HUVECs and hMSCs were cultured in endothelial cell growth medium 2 (PromoCell, Heidelberg, Germany, cat. C-22011) and mesenchymal stem cell growth medium 2 (PromoCell, cat. C-28009), respectively. For all experiments, HUVECs and hMSCs were used at passages ≤ 5.

For the pre-screen, cell seeding densities were determined from a titration spanning the range from 5000 to 313 cells per well. The mct-spheroids, including 60% malignant cells, 25% HUVECs, and 15% MSCs, were dispensed as a mixture into U-bottom Ultra-Low Attachment 384-well plates (Corning, NY, USA) (Table 3) [24–26].

### High-throughput Drug Combination Screening:

Prior to their inoculation into 384-well plates, malignant cells, HUVECs, and hMSCs were removed from T flasks using TrypLE express (Thermo Fisher Scientific, Waltham, MA, USA, cat. 12605036) and harvested by centrifugation for 5 min at 233× *g*. Following the removal of the supernatant, the cells were resuspended in fresh medium and counted using a Cellometer auto T4 bright field cell counter (Nexcelom, Lawrence, MA, USA) and trypan blue to distinguish dead cells. Mct-spheroids were grown from a mixture of three cell types: 60% malignant cells, 25% HUVECs, and 15% hMSCs, as described previously [24–26]. Mixed-cell suspensions of 50 μL were dispensed into the wells of 384-well black/clear round-bottom ULA spheroid microplates (Corning Inc., Corning, NY, USA, cat. 3830). Following inoculation, the microplates were transferred to an incubator (Thermo Fisher Scientific, Waltham, MA, USA) and maintained at 37 °C and 5% CO_2_ with 95% humidity. Three days after inoculation, test agents or controls were dispensed into the wells of microplates. The approved and investigational anticancer agents, prepared as 800× stock solutions, were subsequently transferred in 62.5 nL volumes to the appropriate wells of microplates using an I.DOT non-contact dispenser (DISPENDIX, Stuttgart, Germany) to achieve a 1x final concentration. All anticancer agents and their combinations were tested in triplicate. Additionally, each microplate included a DMSO vehicle control (n = 14) and a cytotoxicity control (5 μM staurosporine and 3 μM gemcitabine, n = 14). After the delivery of the test agents and controls, the microplates were returned to the incubator for 7 days. The experiment was terminated by the addition of CellTiter-Glo 3D (CTG3D, Promega, WI, USA), which solubilizes the spheroids and releases ATP, which serves as a surrogate for viable cell number. The CTG3D luminescence endpoint was measured with a standard plate reader, and cell viability was reported as percent treated/control survival. Samples of the cell lines were collected at regular intervals throughout the screening process for short tandem repeat (STR) profiling and mycoplasma testing by Labcorp (Laboratory Corporation of America Holdings, Burlington, NC, USA) to confirm their authenticity and integrity.

### Combination Data Analysis.

Plate readings (.csv files) from the screen were exported to custom Microsoft Excel spreadsheets for analysis. The raw luminescence data were evaluated for quality control, filtered for outliers, and converted to percent viability by normalizing to the DMSO (vehicle) control. Concentration response data were fit with 4-parameter curve fits using the Solver Add-In in Excel. Synergy evaluations were based on the Bliss Independence Model (Webb’s Fractional Product Method). Following Bliss Independence, the expected (additive) effect would be equal to the product of the percent viability for each compound alone [27]. The synergy of the combination of the two compounds would be indicated by a lower-than-expected observed percent viability. Antagonism would be indicated by a higher-than-expected observed percent viability. All data are accessible via the PubChem BioAssay public database: AID 2060343; AID 2060342; AID 2060341; AID 2060340; AID 2060339; AID 2060338; AID 2060337; AID 2060333; AID 2060336; AID 2060334; AID 2060335; AID 2060331; AID 2060332; AID 2060330; AID 2060329; AID 2060328; AID 2060326; AID 2060327; AID 2060325; AID 2060362; AID 2060363; AID 2060358; AID 2060359; AID 2060360; AID 2060357; AID 2060356; AID 2060361; AID 2060355; AID 2060354; AID 2060353; AID 2060350; AID 2060351; AID 2060352; AID 2060349; AID 2060347; AID 2060348; AID 2060346; AID 2060345; AID 2060344; AID 1963875; AID 1963874; AID 1963868; AID 1963873; AID 1963870; AID 1963869; AID 1963867; AID 1963866; AID 1963872; AID 1963871; AID 1963864; AID 1963865; AID 1963861; AID 1963863; AID 1963862; AID 1963860; AID 1963858; AID 1963859; AID 1963857; AID 1963856. See also Supplemental Table S2.

### In vivo experiments:

Animal studies were performed according to the National Cancer Institute (NCI) at Frederick (Frederick, MD, USA) Institutional Animal Care and Use Committee guidelines (IACUC Protocol No. 23–058). Tumors and PDCs were from the NCI Patient-derived Tumor Repository (https://pdmr.cancer.gov/ [cited 2025 May 10]). Tumor fragments (8 mm^3^) were harvested from mice bearing the 171881-019-R-J1 breast carcinoma cancer PDX grown in NSG mice and directly implanted as tissue fragments into the mammary fat pad of 6-week-old female NOD-scid gamma NSG mice weighing approximately 26 g (Frederick National Laboratory for Cancer Research, Biological Testing Branch Animal Production, Frederick, MD, USA). Tumors of the 845751 Head & Neck squamous cell cancer were induced by subcutaneous injection of 0.8–1 × 10^7^ cells into the flank of 6 wk old female NSG mice. The mice were housed in an AAALAC-accredited facility with food and water provided ad libitum. When tumors reached the predetermined starting weight (staging weight) of 200 mg, the animals were randomized into experimental groups and treatment was initiated. Groups included a vehicle control group as well as the drug-treated groups. Drug doses were selected on the basis of prior experience or newly conducted mouse tolerability studies as described elsewhere [28]. Details of dosing, and formulation as shown in Supplementary Table S1. Red bars indicate days of treatment. Tumors were monitored by bidirectional caliper measurements, and the tumor weights were calculated as tumor volume was calculated as Volume = 0.5 × Length × Width2 [29, 30]. Data collection was performed using the StudyLog software program StudyDirector (Studylog Systems, Inc., South San Francisco, CA, USA). Data were calculated and plotted using Microsoft EXCEL.

## 3. Results

**Figure 1 •:**
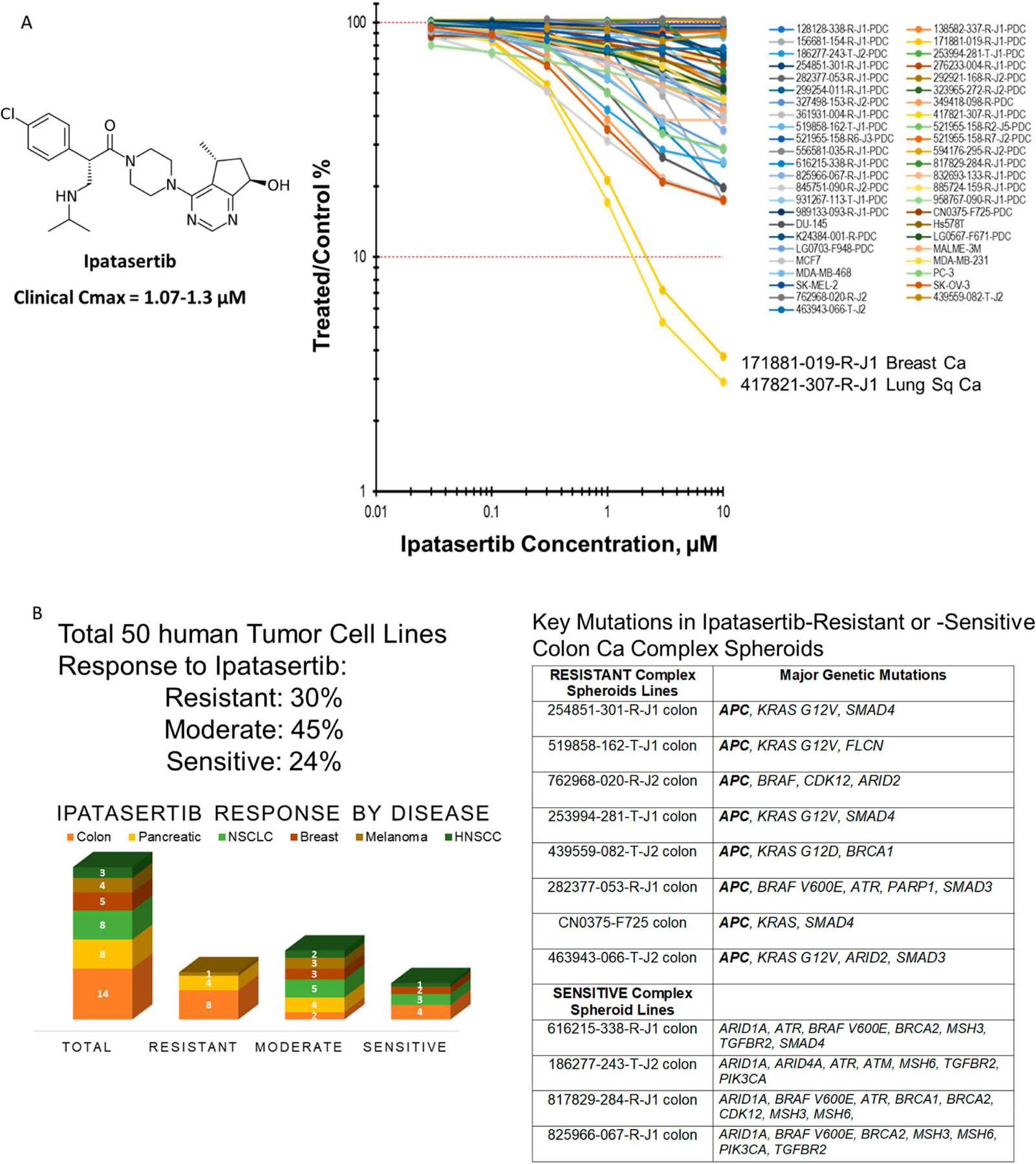
(A) Concentration response and chemical structure of ipatasertib. The concentration response for ipatasertib in 42 human tumor cell lines grown as mct-spheroids. The cell lines were deemed sensitive if 1-log or more cells were killed (blue), moderately responsive if an IC50 was reached (black), and non-responsive if an IC50 was not reached (red) after 7-day exposure to ipatasertib. (B) The 42 human tumor cell lines were of 6 types: colon, pancreatic, NSCLC, melanoma, breast, and HNSCC. The resistant group included colon, pancreatic, and melanoma lines. All tumor types were represented in the moderate response group. The resistant colon lines all harbored mutant APC.

The highly selective pan-AKT inhibitor ipatasertib has been extensively studied in clinical trials. Clinical trials.gov (https://clinicaltrials.gov/ [cited 2025 May 10]) lists 57 clinical trials involving ipatasertib, which cover solid tumors, both adenocarcinomas and squamous carcinomas, with a major focus on breast carcinomas (Table S3). The clinical Cmax concentration for ipatasertib is 2.0 micromolar. Ipatasertib was tested in combination with other signaling pathway inhibitors along the RAS-RAF-MEK-ERK pathway and along the PI3K-AKT-mTOR pathway in doublets in patient-derived human tumor cell lines and well-established human tumor cell lines in mct-spheroids. The concentration response data for ipatasertib in human tumor mct-spheroids after a 7-day exposure to ipatasertib over a concentration range from 10 μM to 0.03 μM allowed the cell lines to be grouped into three classes of response (Figure 1A). The tumor cell lines tested were from six tumor types: colon, pancreas, NSCLC, breast, melanoma, and HNSCC. Overall, 30% of the mct-spheroids were resistant to ipatasertib, in that an IC_50_ was not reached with these lines; 45% were moderately responsive, in that an IC_50_ was reached in the 7-day exposure. However, only two lines were sensitive, with 1-log of cytotoxicity reached in the 7-day exposure (Figure 1B). Among the fourteen colon carcinoma lines, eight were resistant and four were sensitive. All the ipatasertib-resistant colon carcinoma lines were APC-mutant [31, 32].

**Figure 2 •:**
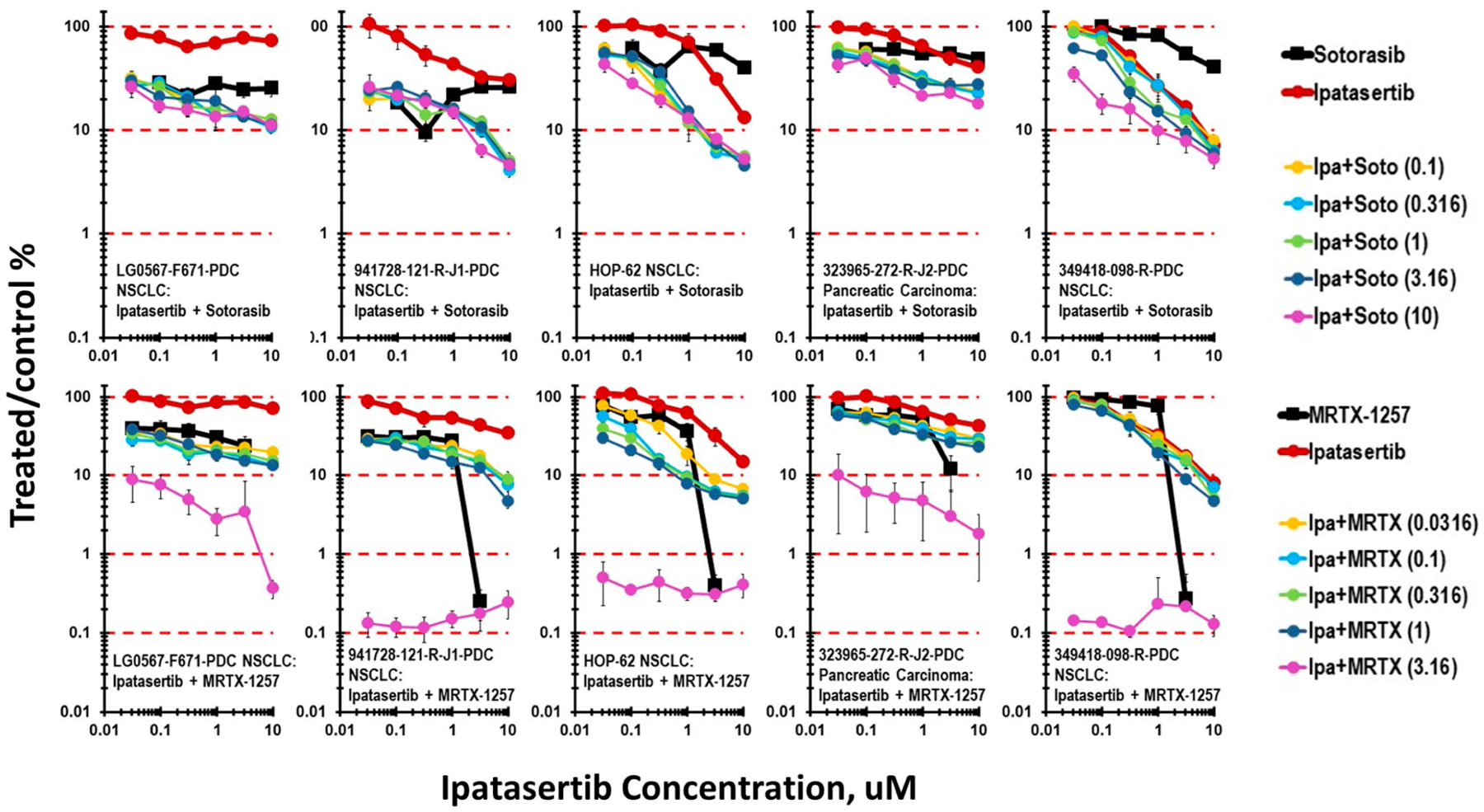
Concentration response data for the KRAS G12C inhibitors sotorasib and MTRX-1257 tested in combination with ipatasertib. The NSCLC cell lines LG-0567-F671, 941728-121-R-J1, and HOP-62 and the pancreatic cancer line 323695-272-R-J2 are KRAS G12C-mutated, and the NSCLC line 349418-098-R is KRAS wildtype.

Two KRAS G12C inhibitors, sotorasib and MTRX-1257, were tested in simultaneous combination with ipatasertib. The concentration response data shown in Figure 2 highlight the findings. Three of the lines, LG0567-F671, 941728-121-R-J1, and HOP-62, are non-small-cell lung cancers harboring the KRAS G12C mutation. The 323965-272-R-J2 pancreatic cancer also harbors the KRAS G12C mutation, while the 349418-098-R non-small-cell line is KRAS wildtype. Sotorasib has a relatively flat concentration response in the four KRAS G12C mutant cell lines; however, additivity between ipatasertib and sotorasib cytotoxicity is evident. The KRAS wildtype line is responsive to ipatasertib, and an indication of additive cytotoxicity between ipatasertib and sotorasib at the highest sotorasib concentrations is evident. Except at the highest concentration tested (3 μM), the tool compound MRTX-1257 has a flat concentration response. Additive cytotoxicity between ipatasertib and MRTX-1257 was evident, except in the KRAS wildtype line.

**Figure 3 •:**
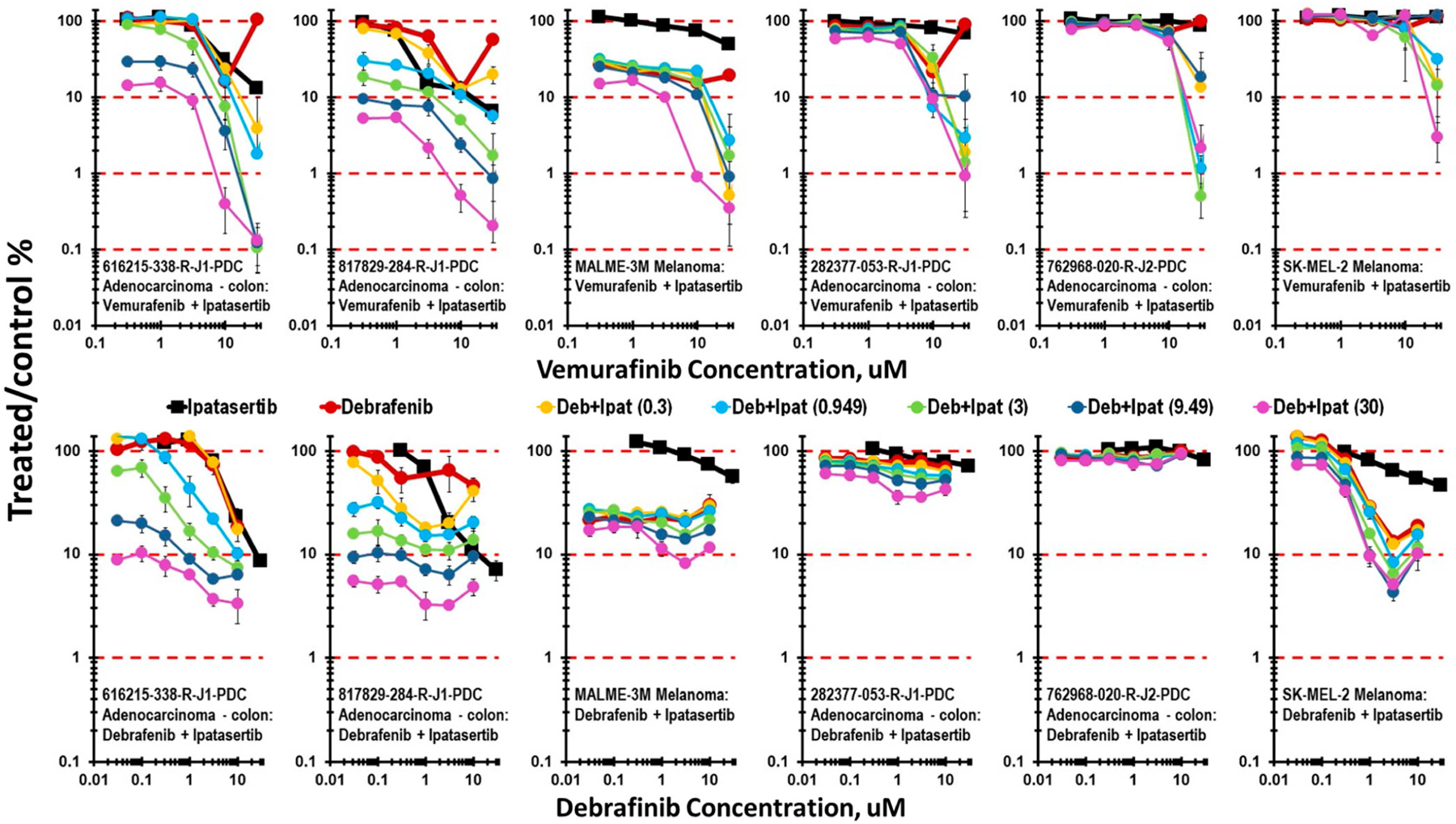
Concentration response data for the BRAF V600E inhibitors vemurafenib and debrafenib tested in combination with ipatasertib. The colon carcinoma cell lines 616215-338-R-J1, 817829-248-R-J1, and 283377-053-R-J1 and the melanoma cell line MALME-3M are BRAF V600E-mutated, while the colon carcinoma cell line, 762968-020-R-J2 contains a BRAF D594N variant and the melanoma cell line SK-MEL-2 is BRAF wildtype.

The combination of ipatasertib with BRAF V600E mutant and BRAF wildtype mct-spheroids is shown in Figure 3. Two BRAF V600E selective inhibitors vemurafenib (clinical Cmax 127 μM) and dabrafenib (clinical Cmax 4.86 μM) were tested in combination with ipatasertib in two BRAF V600E mutant colon carcinoma lines, 616215-338-R-J1 and 817829-284-R-J1, and in a single BRAF V600E mutant melanoma line, MALME-3M. Over the concentration ranges tested, both vemurafenib and dabrafenib resulted in additive cytotoxicity in the BRAF V600E mutant lines, resulting in 1- to 1.5-logs of cytotoxicity over the 7-day exposure time. The combination of vemurafenib with ipatasertib had no effect beyond that of ipatasertib alone on the two BRAF V600E mutant colon carcinoma lines 282377-053-R-J1 and 762968-020-R-J2 or the BRAF wildtype melanoma line SK-MEL-2, except at the very highest concentrations of the BRAF inhibitors.

**Figure 4 •:**
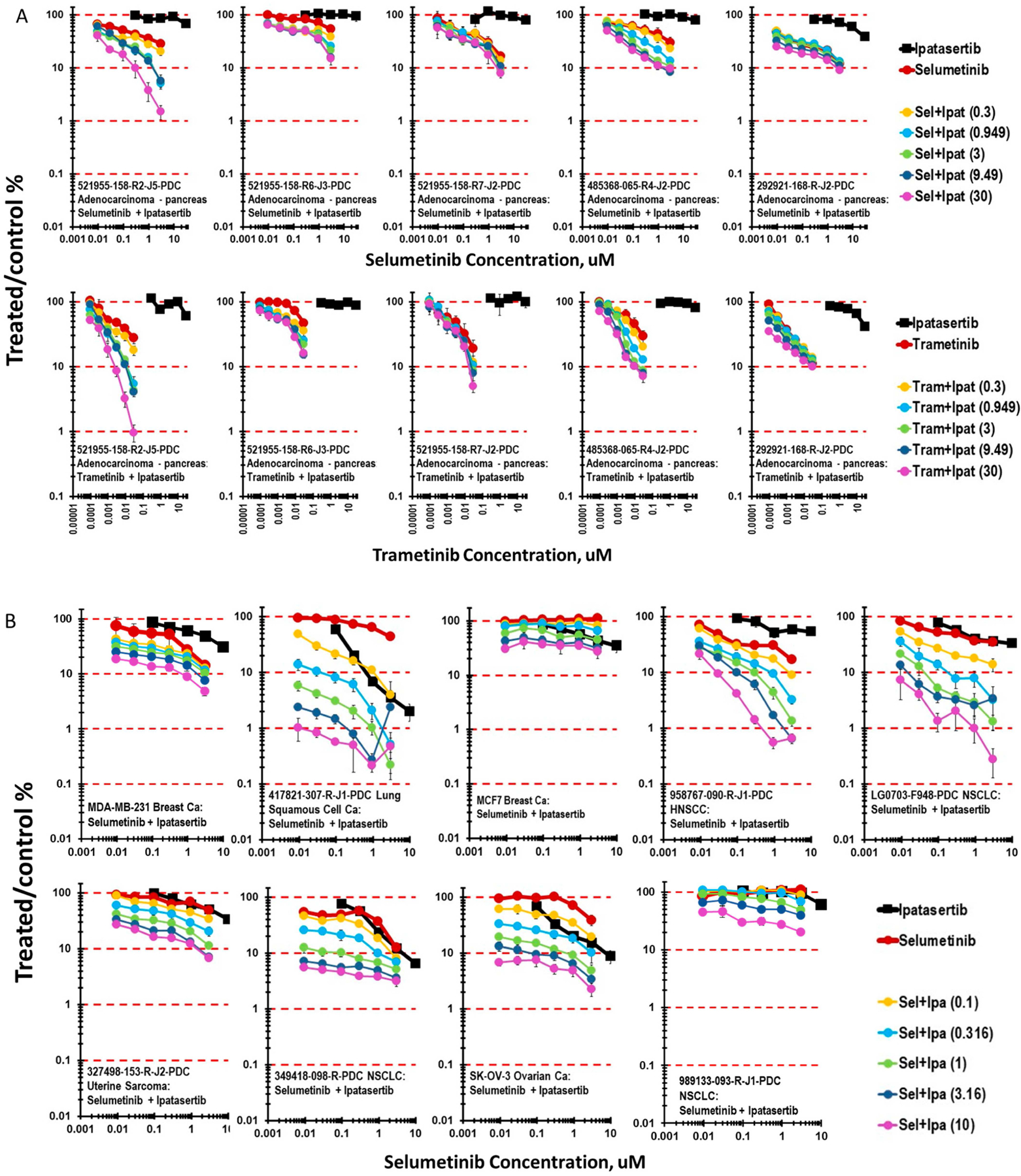

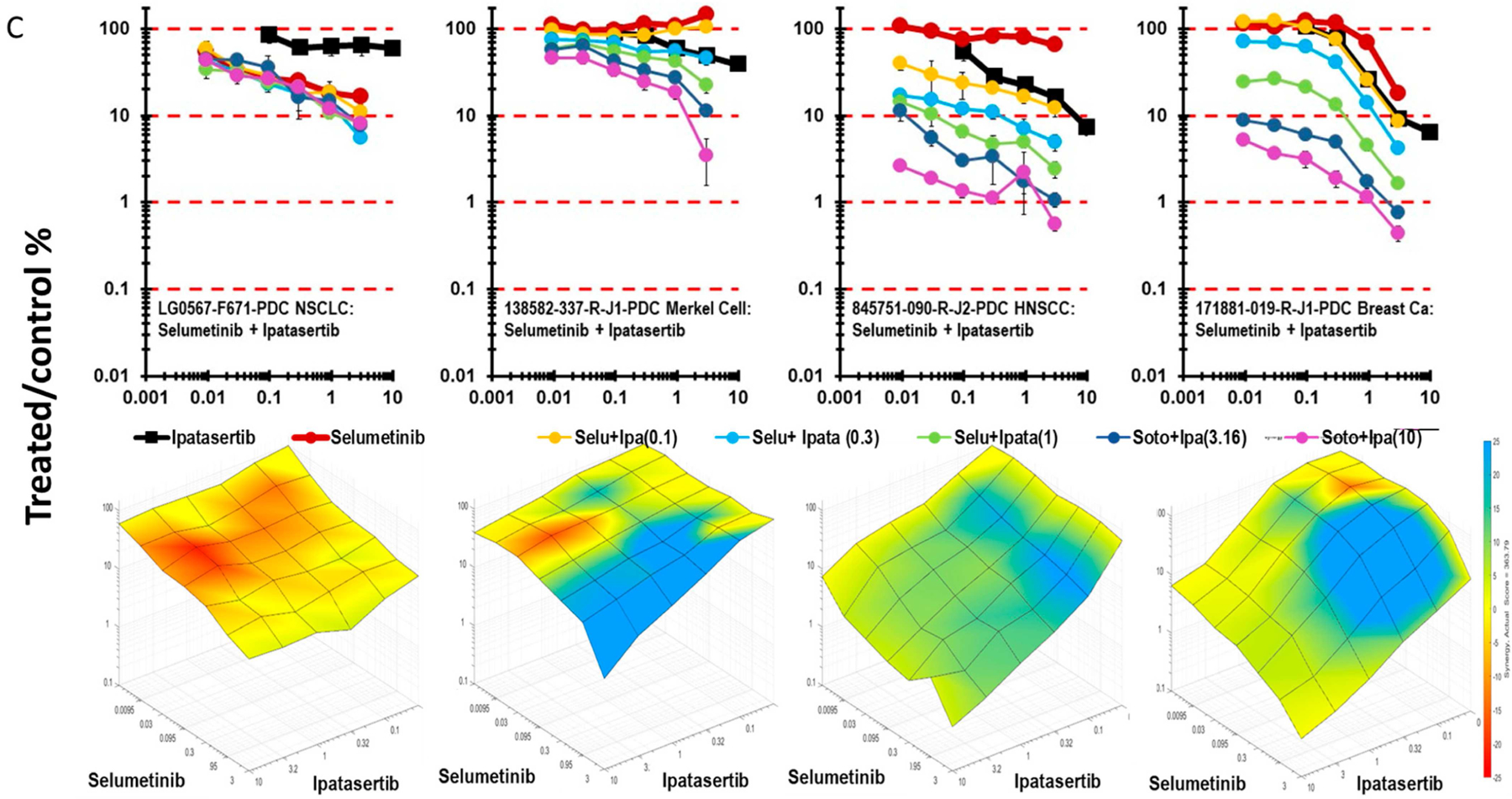
(A) Concentration response data for the MEK inhibitors selumetinib and trametinib tested in combination with the AKT inhibitor ipatasertib. The pancreatic carcinoma cell lines 521955-158-R2-J5, from a liver metastasis, 521955-158-R5-J6, from a pancreas tumor, and 521955-158-R7-J2, from a colon metastasis, were derived from a single patient. (B) Concentration response data for nine patient-derived tumor cell lines exposed to the combination of ipatasertib and selumetinib. There was >2 logs of cell killing by the combination for 3 out of 9 lines and >1 log of killing for 4 out of 9 lines. (C) Concentration response data for four patient-derived tumor cell lines exposed to the combination of ipatasertib and selumetinib along with the response surface plot for each. Response surface areas which are red indicate less-than-additive cytotoxicity from the combination, areas which are yellow indicate additive cytotoxicity by the combination, and areas which are blue indicate greater-than-additive cytotoxicity by the combination.

Several patient-derived cell lines were developed from 521955-158 tissue samples from a single patient. The 521955-158-R5-J3 specimen was from a pancreas tumor, the 521955-158-R2-J5 was from a liver metastasis and the 521955-158-R7-J2 was from a colon metastasis. The response of these three patient-derived cell lines grown as mct-spheroids to the combination of ipatasertib with a MEK inhibitor, selumetinib or trametinib (clinical Cmax of 1.74 or 0.021 μM), was assessed (Figure 4A). While ipatasertib had little activity as a single agent, both MEK inhibitors reached an IC_50_ in all three 521955-158 lines. The line from the pancreas tumor was the least sensitive to ipatasertib plus a MEK inhibitor, reaching less than 1-log of cytotoxicity with ipatasertib in combination with selumetinib or trametinib. The most responsive cell line was derived from the liver metastasis 521955-158-R2-J3, with 2-logs of cytotoxicity with a MEK inhibitor and ipatasertib combination. Exposure to the combination of ipatasertib and selumetinib or trametinib resulted in more than 1-log of cytotoxicity with mct-spheroids of the colon metastasis 521955-158-R7-J2. Each of these lines had the same key genetic changes with mutations in KRAS (G12D), ATR, and GNAQ; therefore, a deeper study would be needed to understand the difference in response.

Among the cell lines grown as mct-spheroids which were most responsive to ipatasertib as a single agent were a lung squamous carcinoma cell line 417821-307-R-J1, a head and neck squamous carcinoma line 958767-090-R-J1, and a non-small-cell lung carcinoma line LG0703-F948. Exposure of each of these lines grown as mct-spheroids to the combination of ipatasertib and selumetinib resulted in more than 2-logs of cytotoxicity (Figure 4B). The other four lines shown were responsive to this combination but less so, each reaching 1-log of cytotoxicity. Surface plots demonstrated the response to ipatasertib and selumetinib of mct-spheroids in the LG0567-F671 NSCLC ranged from sub-additive to additive, while surface plots of ipatasertib in combination with selumetinib of mct-spheroids of 138582-337-R-J1 Merkel cell tumor, 845751-090-R-J2 H&N squamous carcinoma, and 171881-019-R-J1 breast carcinoma had regions of greater-than-additive cytotoxicity over the range of ipatasertib concentrations (Figure 4C).

**Figure 5 •:**
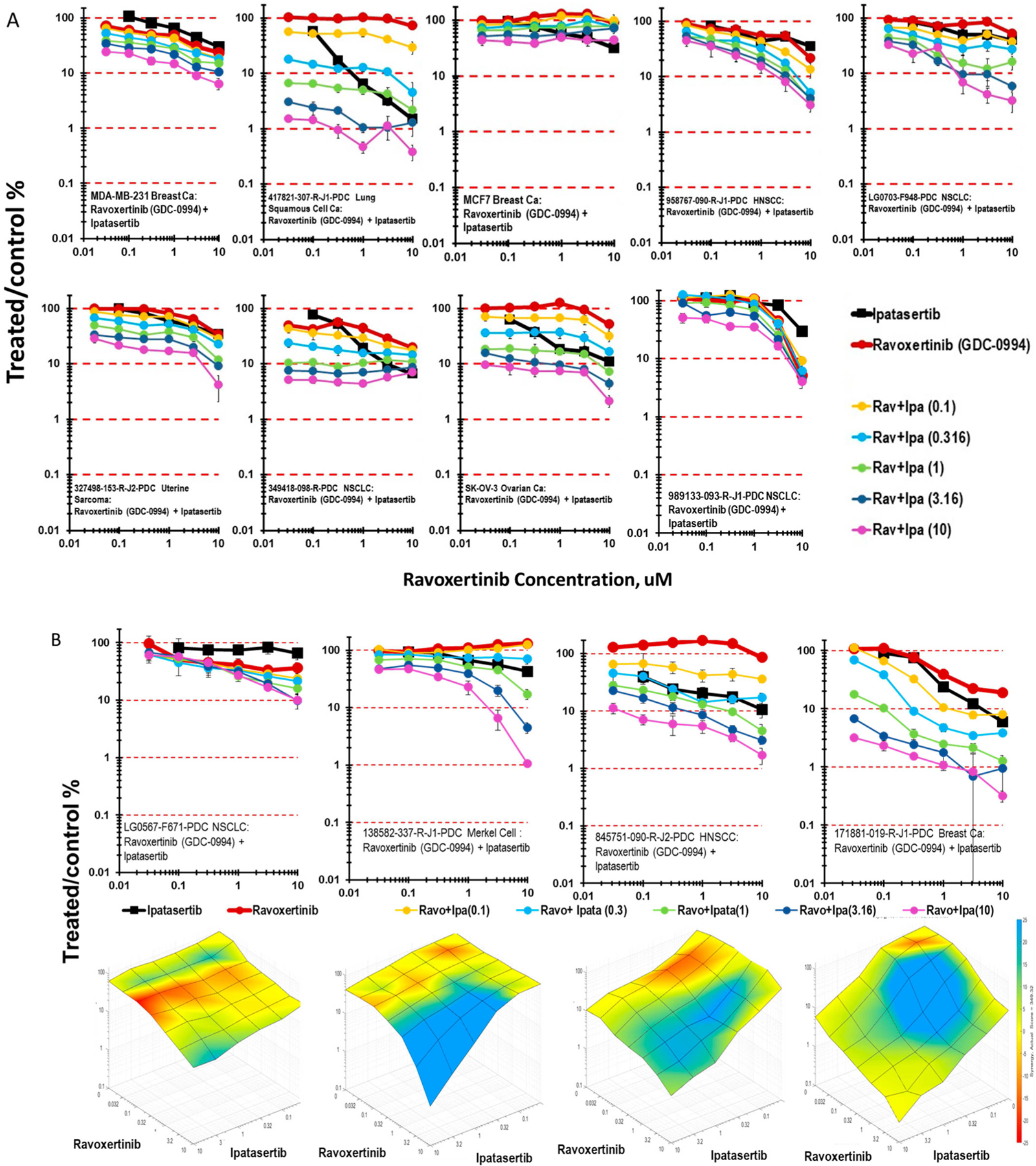
(A) Concentration response data for nine patient-derived tumor cell lines and the SK-OV-3 ovarian cancer cell line exposed to the combination of the AKT inhibitor ipatasertib and the ERK1/2 inhibitor ravoxertinib. The 417821-307-R-J1 lung squamous-cell carcinoma was more sensitive to the combination than the other lines. (B) Concentration response data for four patient-derived tumor cell lines exposed to the combination of ipatasertib and ravoxertinib along with the response surface plot for each. Response surface areas which are red indicate less-than-additive cytotoxicity from the combination, areas which are yellow indicate additive cytotoxicity by the combination, and areas which are blue indicate greater-than-additive cytotoxicity by the combination.

The next step along the RAS-RAF-MEK-ERK signaling pathway is ERK inhibition. The ERK1/2 inhibitor ravoxertinib was tested in combination with ipatasertib (Figure 5A). Exposure to the combination of ipatasertib and ravoxertinib produced more than 2-logs of cytotoxicity in the lung squamous carcinoma cell line 417821-307-R-J1, as was observed with the MEK inhibitor selumetinib. The combination of ipatasertib and ravoxertinib was primarily additive in the other six cell lines highlighted, resulting in 1- to 2-logs of cytotoxicity with the combination. Surface plots allow for a comparison of the response of mct-spheroids to ipatasertib and selumetinib with their response to ipatasertib and ravoxertinib. The combination of ipatasertib and ravoxertinib in the LG0567-F671 NSCLC ranged from sub-additive to additive cytotoxicity, while surface plots of ipatasertib in combination with ravoxertinib in the 138582-337-R-J1 Merkel cell tumor, 845751-090-R-J2 H&N squamous carcinoma, and 171881-019-R-J1 breast carcinoma mct-spheroids have regions of greater-than-additive cytotoxicity over the range of ipatasertib concentrations (Figure 5B).

**Figure 6 •:**
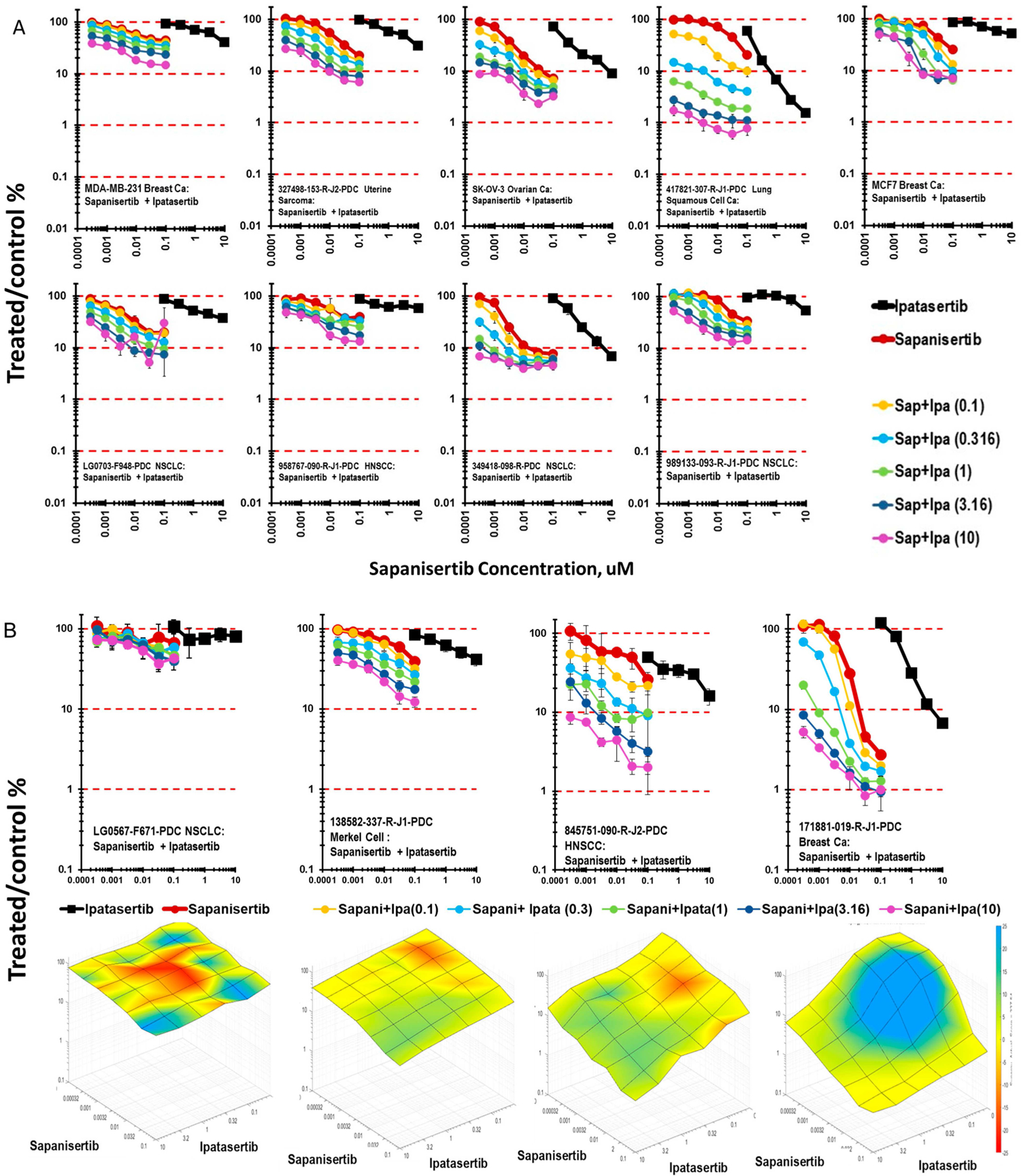
(A) Concentration response data for nine patient-derived tumor cell lines and the SK-OV-3 ovarian cancer cell line exposed to the combination of the AKT inhibitor ipatasertib and the mTORC1/2 inhibitor sapanisertib. The 417821-307-R-J1 lung squamous cell carcinoma was more sensitive to the combination than the other lines. (B) Concentration response data for four patient-derived tumor cell lines exposed to the combination of ipatasertib and sapanisertib along with the response surface plot for each. Response surface areas which are red indicate less-than-additive cytotoxicity from the combination, areas which are yellow indicate additive cytotoxicity by the combination and areas which are blue indicate greater-than-additive cytotoxicity by the combination.

In the PI3K-AKT-mTOR signaling pathway, ipatasertib was tested in combination with the mTORC1/2 inhibitor sapanisertib. Overall, the combination of ipatasertib and sapanisertib was additive in the nine lines grown as mct-spheroids shown in Figure 6A. The most responsive lines were the lung squamous carcinoma cell line 417821-307-R-J1, where 2-logs of cytotoxicity were achieved with ipatasertib and sapanisertib in combination with a 7-day exposure. More than 1-log of cytotoxicity occurred with the combination of ipatasertib and sapanisertib in all but the 138582-337-R-J1 Merkel cell tumor mct-spheroids after a 7-day exposure to the combination. Surface plots show that the response to ipatasertib and sapanisertib in mct-spheroids was additive to sub-additive in the LG0567-F671 NSCLC, the 138582-337-R-J1 Merkel cell tumor, and 845751-090-R-J2 H&N squamous carcinoma. The 171881-019-R-J1 breast carcinoma, however, had regions of greater-than-additive cytotoxicity over the range of ipatasertib concentrations (Figure 6B).

**Figure 7 •:**
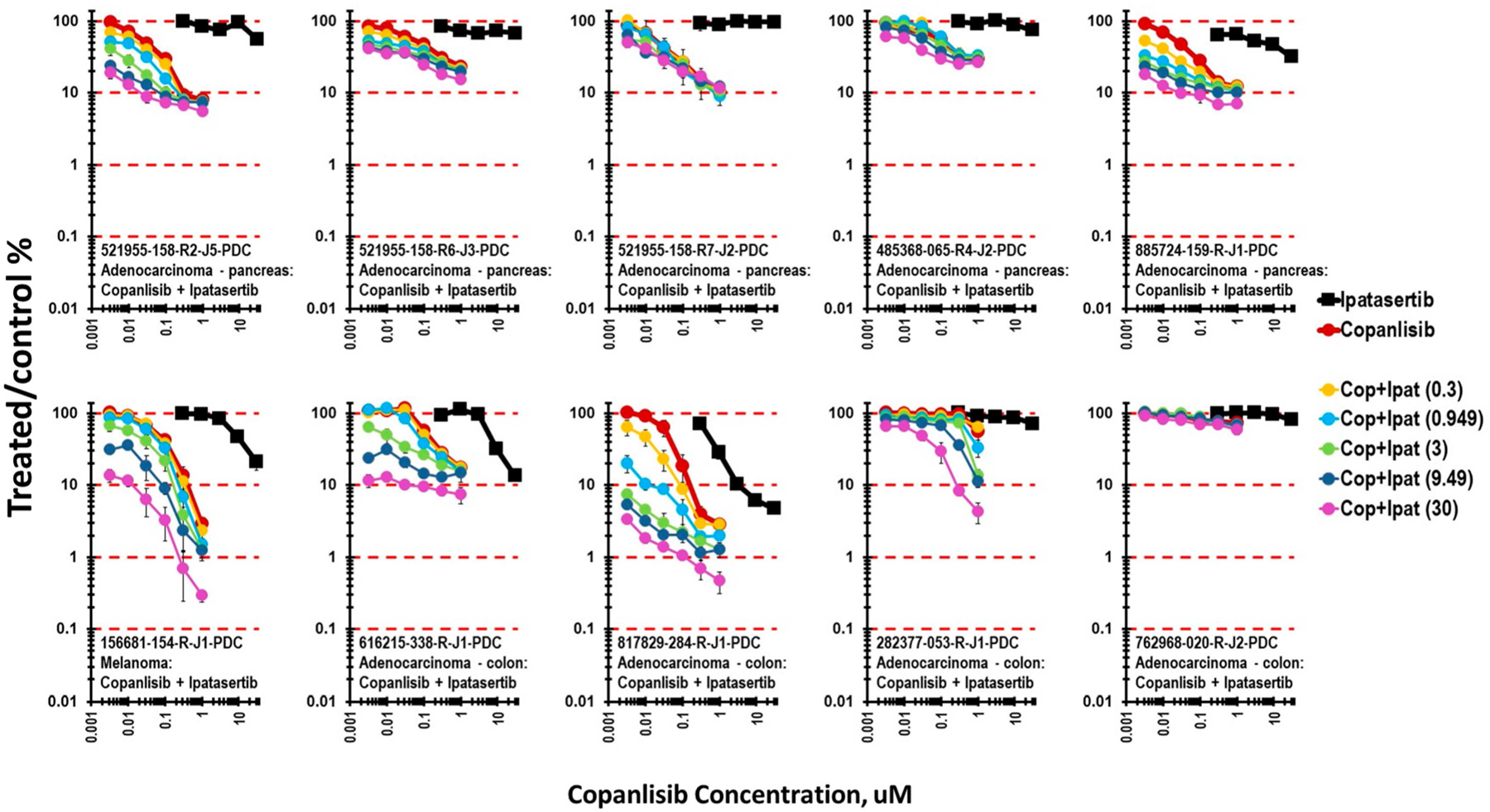
Concentration response data for ten patient-derived tumor cell lines exposed to the combination of the AKT inhibitor ipatasertib and the PI3K inhibitor copanlisib. The 156681-154-R-J1 melanoma and the 817829-284-R-J1 colon carcinoma were most responsive to the combination.

Copanlisib (clinical Cmax 0.964 μM), an inhibitor of PI3K kinase, is active in the PI3K-AKT-mTOR signaling pathway. The combination of copanlisib and ipatasertib represents sequential inhibition along the signaling pathway. Five pancreatic cancer cell lines, four colon carcinoma cell lines, and one melanoma cell line grown as mct-spheroids were exposed to copanlisib and ipatasertib for 7 days (Figure 7). The 521955-158 series of pancreatic carcinoma cell lines had a pattern of response like that observed with the combination of ipatasertib with MEK inhibitors. The 521955-158-R2-J3 line, derived from a liver metastasis, was most responsive to the combination of copanlisib and ipatasertib; the 521955-158-R5-J3 line, derived from a pancreas tumor, was moderately responsive to the combination; and the response of the 521955-158-R7-J2 colon metastasis was not altered from the response to copanlisib alone by the addition of ipatasertib. The pancreatic carcinoma line 485368-065-R4-J2 was moderately responsive to the combination of ipatasertib and copanlisib, while the pancreatic carcinoma line 885274-159-R-J1 had a similar response to the combination of copanlisib and ipatasertib as did the 521955-158-R2-J3 line, derived from a liver metastasis. When grown as mct-spheroids, the colon carcinoma cell lines 616215-338-R-J1, 817829-284-R-J1, 282377-053-R-J1, and 762968-020-R-J2 responded heterogeneously to the combination of ipatasertib and copanlisib. The 817829-284-R-J1 colon carcinoma line was responsive to the combination of ipatasertib and copanlisib with 2-logs of cytotoxicity, the 616215-338-R-J1 and 282-377-053-R-J1 colon carcinoma lines were moderately responsive with 1-log of cytotoxicity, while 762968-020-R-J1 was not responsive with cytotoxicity, which was the same as for the single agent copanlisib.

Patient-derived xenograft (PDX) tumor growth delay studies were carried out with the 171881-019-R breast carcinoma and the 845751-090-R head and neck squamous carcinoma to explore the efficacy of the combinations of ipatasertib with selumetinib, ravoxertinib, or sapanisertib (Figures S1 and S2). The tumor volume doubling time for the 171881-019-R breast carcinoma ranged from 7 to 14 days in the controls. The 845751-090-R head and neck squamous carcinoma was slower growing; the control tumor volume doubling times ranged from 24 to 30 days. The drugs were administered orally. The details of the drug formulations and schedule of administration are shown in Table S1. Ipatasertib was well tolerated at doses of 60 mg/kg and 30 mg/kg administered in a twice-daily regimen for a total of 20 days by mice bearing the 171881-019-R breast carcinoma. The combination of ipatasertib with selumetinib (20 or 15 mg/kg) was well tolerated. The most effective treatment regimen was ipatasertib (60 mg/kg) in combination with selumetinib (20 mg/kg), which resulted in tumor control during the treatment period; however, the tumor resumed growing at the same rate as the control tumors at the completion of the dosing period (Figure S1). The combination of ipatasertib (60 mg/kg) and selumetinib (20 mg/kg) was more effective in the 845751-090-R head and neck squamous carcinoma, resulting in stable disease during the dosing period and slower growth than the control thereafter (Figure S2). Ravoxertinib (30 mg/kg and 20 mg/kg) was well tolerated in animals bearing either the 171881-019-R breast carcinoma or the 845751-090-R head and neck squamous carcinoma (Figures S1 and S2). The combination of ipatasertib (60 mg/kg) and ravoxertinib (30 mg/kg) was a less effective therapy than ipatasertib in combination with selumetinib, and there was progressive disease in both PDX models during treatment with ipatasertib plus ravoxertinib. In the 171881-019-R breast carcinoma, the dose of ipatasertib in combination with sapanisertib was reduced to 30 mg/kg. The combination of ipatasertib (30 mg/kg) with sapanisertib (1 mg/kg) resulted in stable disease during the treatment period and tumor growth at the same rate as the control tumor after the completion of dosing (Figure S1). Animals bearing the 845751-090-R tumor were treated with ipatasertib (60 mg/kg) in combination with sapanisertib (1 mg/kg). Toxicity was observed, with 4 out of 5 animals succumbing to drug-related toxicity on day 36. One mouse continued on the study and progressive, slow tumor growth was observed (Figure S2).

## 4. Discussion

Most cell surface receptors stimulate intracellular proteins which are linked to G protein-coupled receptors or kinase-stimulated signaling proteins. These intracellular components stimulate downstream signaling, which propagates and amplifies the signal. AKT plays a central role in human malignancy, and AKT activation can contribute to all the hallmarks of cancer [6]. Despite the central role of AKT in the processes involved in malignancy, drugs targeting AKT do not elicit durable responses when used as monotherapies. The critical need for AKT activity in normal tissues makes finding therapeutic benefit difficult. The targets of signaling pathways frequently are transcription factors connecting the cell surface to the nucleus, leading to changes in gene expression. There are many potential drug targets downstream of cell surface receptors, often through RAS, including in the PI3K-AKT-mTOR pathway and the RAF-MEK-ERK pathway. Gene expression of PI3K-AKT-mTOR signaling axis pathway members in the PDMR cell lines used in the current study are shown in Figure S3. PI3K-AKT-mTOR intracellular signaling is key in cell growth, survival, and metabolism in cancer and is an established oncogenic driver pathway [4, 6].

The MEK kinase inhibitor selumetinib was the first therapy approved for pediatric patients 2 years of age and older with symptomatic, inoperable plexiform neurofibromas (PNs). Selumetinib is a non-ATP-competitive agent that locks MEK1/2 into an inactive conformation; thus, selumetinib works by binding to the MEK1/2 allosteric site, locking the protein into an inactive conformation, preventing ATP binding and substrate access, and disrupting the molecular interventions needed for ERK1/2 activation. By inhibiting the MEK1/2 pathway, selumetinib reduces cell proliferation and promotes pro-apoptotic signal transduction [13, 14]. In preclinical studies, selumetinib arrested the cell cycle in the G1-S phase and induced apoptosis. Selumetinib selectively inhibits MEK1 and MEK2, which can effectively blunt the pleiotropic effects of the RAS-RAF-MEK-ERK cascade, reducing cell proliferation and promoting pro-apoptotic signal transduction.

Ravoxertinib, an inhibitor of extracellular signal-regulated kinase (ERK) with potential antineoplastic activity, has been approved by the FDA to treat melanoma patients with BRAF V600E mutation. Ravoxertinib inhibits both ERK phosphorylation and activation of ERK-mediated signal transduction pathways, thus preventing ERK-dependent tumor cell proliferation [2]. The mitogen-activated protein kinase (MAPK)/ERK pathway is upregulated in a variety of tumor cell types and plays a key role in tumor cell proliferation, differentiation, and survival [2]. Sapanisertib is a mTOR inhibitor that targets both mTORC1 and mTORC2. Sapanisertib was granted fast-track designation by the FDA for the treatment of adults with unresectable or metastatic squamous non-small-cell lung cancer (NSCLC) who have a mutation in nuclear factor erythroid 2-related factor (NRF2) and who have received prior platinum-based chemotherapy and immune checkpoint inhibitor therapy [33, 34]. The PI3K inhibitor copanlisib was approved by FDA in 2017 for the treatment of adult patients with relapsed follicular lymphoma [35, 36]. However, in 2023, the FDA acknowledged Bayer’s request to withdraw the approval of copanlisib. The withdrawal was due to the results of the CHRONOS-4 study, which found that the addition of copanlisib to standard immunochemotherapy regimens did not meet the primary endpoint of progression-free survival benefit [37].

The FDA granted accelerated approval to sotorasib in mid-2021 for the treatment of patients with a KRAS G12C mutation who have received at least one prior line of therapy. Sotorasib was the first KRAS G12C inhibitor to be approved in the United States. The FDA’s approval was based on data from the phase 2 CodeBreaK 100 trial, which showed that 36% of patients had an objective response and 58% had a duration of response of at least 6 months. The FDA issued a complete response letter to sotorasib’s supplemental new drug application in December 2023, declining to convert the accelerated approval to traditional approval [38, 39]. Over 40 inhibitors of these critical cellular pathways have entered the clinical trial, and a few PI3K, mTOR, and KRAS inhibitors are now approved drugs. While drugs targeting these pathways have had promising results, resistance and normal tissue toxicity remain challenging.

While a plethora of selective compounds which target members of the major metabolic pathways downstream from RAS have been developed and additive to greater-than-additive activity can be achieved with combinations of the agents in preclinical models, demonstrating anticancer activity without untoward normal tissue toxicity has been difficult in the clinic.

Ipatasertib exposure often produces greater-than-additive cytotoxicity in combination with chemotherapy preclinically. Although ipatasertib inhibits all three isoforms of the AKT enzyme, ipatasertib achieved only one of two co-primary endpoints in Phase III trials in prostate cancer and mTNBC [31, 32]. The development of ipatasertib was discontinued in late 2023. Capivasertib is an approved AKT inhibitor which is used in combination with the estrogen receptor antagonist fulvestrant for adults with hormone receptor (HR)-positive, HER2-negative locally advanced or metastatic breast cancer and one or more biomarker alterations in *PIK3CA*, *AKT1*, or *PTEN* [31].

## 5. Conclusions

Most investigational anticancer agents developed through preclinical studies in cell culture and animal models look promising until clinical trial. While a lack of clinical activity predictability in cell-based and human xenograft preclinical models is often blamed, the failure of ipatasertib to reach FDA approval cannot be attributed to the lack of translation of preclinical models. There are more than 20 AKT inhibitors in clinical trials, some in cancer, some in other disease areas. Some of the compounds in clinical trials are ATP-competitive and some bind to allosteric sites. There are three isoforms of AKT, which have varied tissue distribution and functions. Early ATP-competitive AKT inhibitors, including ipatasertib, do not distinguish amongst the three isoforms, leading to toxicity. The PDMR cell lines used in the current study express very similar levels of AKT1 and AKT2 and lower levels of AKT3 (Supplemental Figure S3). These findings illustrate the therapeutic advantage which could be achieved with an isoform-selective compound. The limited safety margin of ipatasertib combinations tested in the clinic led to the discontinuation of ipatasertib. The lesson learned may be that combinations focusing on blocking a metabolic pathway critical in both malignant and normal tissues at multiple nodes may be powerfully toxic and not sufficiently tumor selective. Combinations of ipatasertib with immunotherapy agents, drugs targeting other tumor-selective pathways, or hormonal agents led to the approval of capivasertib [40]. Improving efficacy and minimizing toxicity through combination therapies, isoform-specific inhibitors, elucidating targetable mutations, and identifying biomarkers and precision medicine approaches may be able to improve clinical efficacy for AKT inhibitors.

## Supplementary Material

Supplementary Material

The supplementary materials are available at https://doi.org/10.20935/AcadOnco7726 and include: Table S1. In vivo study drugs dosing, route and schedule of administration; Table S2. Data files accessible via the PubChem BioAssay public database; Table S3. Ipatasertib clnical trials from clinical trials.gov; Figure S1. Response of 171881-019-R breast carcinoma sc tumor PDX to ipatasertib with selumentinib, ravoxertinib or sapanisertib; Figure S2. Response of 845751-090-R H&N squamouse ca sc tumor PDX to ipatasertib with selumentinib, ravoxertinib or sapanisertib; and Figure S3. Gene expression of PI3K-AKT-mTOR signaling axis pathway members in the PDMR cell lines used in the current study.

## Figures and Tables

**Table 1 •:** Drugs and investigational agents tested as single agents and in combination with ipatasertib, showing NSC number, molecular weight, clinical Cmax concentration, if known, and molecular target.

Drug or investigational agent	NSC #	Mol Wt	Clin C_max_ or 10 μM (default)	Target
Ipatasertib	767898	458	10 μM	Akt
Selumetinib	741078	457.68	1.74 μM	MEK
Trametinib	758246	615	0.021 μM	MEK
Adavosertib	754352	500.6	10 μM	WEE1
Everolimus	733504	958.24	0.064 μM	mTORC1
Sapanisertib	764658	309.33	0.08 μM	mTORC1/2
Vemurafenib	761431	489.92	127 μM	Raf
Debrafenib	764134	520	4.86 μM	Raf
Venetoclax	766270	468	4.48 μM	Bcl2
Copanlisib	809693	480.53	0.964 μM	PI3K
Alpelisib	765974	441	5.6 μM	PIK3CA
Palbociclib	758247	448	0.101 μM	CDK4/6
Ravoxertinib	781343	439.85	10 μM	ERK1/2
BMS-387032	767048	380.5	10 μM	CDK2/7/9
Sotorasib	818433	561	10 μM	KRAS G12C
Talazoparib	767125	380.4	0.043 μM	PARP
Afuresertib	778305	427.3	10 μM	Akt

**Table 2 •:** Ipatasertib combination study (PDMR) and standard cell lines screened as mct-spheroids. The disease type and selected genetic properties for each line are shown.

Cell line	Tumor type	Key genetic alterations
171881-019-R-J1	Breast Ca	PIK3CA; KEAP1; TP53; MSH3; MLH1; ATXN2
MDA-MB-231	Breast Ca	TNBC; MUC16(CA125); KRAS G12D; NF2
MDA-MB-468	Breast Ca	TNBC; MUC16(CA125); BRCA2; DDR2
Hs578T	Breast Ca	TNBC; HRAS G12D; EPAS1
MCF7	Breast Ca	PIK3CA; POU2F3; MUC16(CA125)
CN0375-F725	Colon Ca	KRAS A146T; SOX9; SMAD4; APC
616215-338-R-J1	Colon Ca	BRAF V600E; ATR; JAK1; TGFbR2
439559-082-T-J2	Colon Ca	KRAS G12D; BRCA1; SOX9; APC
463943-066-T-J2	Colon Ca	KRAS G12V; TCF7L2; SMAD3; SOX9; APC
519858-162-T-J1	Colon Ca	KRAS G12V; APC
762968-020-R-J2	Colon Ca	BRAF D594N; CDK12; APC
817829-284-R-J1	Colon Ca	PTEN del; BRAF V600E; EGFR; MSH3; ATR; BRCA2; MSH6; JAK1; BCL10; PIK3R; TP53
825966-067-R-J1	Colon Ca	BRAF V600E; MSH2; MSH6; TGFbR2; NOTCH2; PIK3CA; EGFR V769A
254851-301-R-J1	Colon Ca	KRAS G12D; APC
276233-004-R-J1	Colon Ca	KRAS G12S
361931-004-R-J1	Colon Ca	BRAF V600E; PIK3CA H1047R
282377-053-R-J1	Colon Ca	PTEN del; APC; BRAF V600E; ATR; PARP1; SMAD3
186277-243-T-J2	Colon Ca	KRAS G12D; PIK3Ca C420R
931267-113-T-J1	Colorectal Ca	KRAS G12D; PIK3CA E545K
253994-281-T-J1	Colorectal NOS	KRAS G12V; APC
832693-133-R-J1	HNSCC	CDKN2A; RASA1
845751-090-R-J2	HNSCC	PTEN loss; ASXL1; ATM; TP53
958767-090-R-J1	HNSCC	EPHA3; TP53; KMT2C
128128-338-R-J1	Melanoma	BRAF V600K; SMARCA4;
156681-154-R-J1	Melanoma	BRAF V600E; ARID1A; CDKN2A
MALME-3M	Melanoma	BRAF V600E
SK-MEL-2	Melanoma	NRAS G61A
299254-011-R-J1	Melanoma	KRAS G12C; BRAF G466E
138582-337-R-J1	Merkel Cell	PTEN; PMS2
417821-307-R-J1	Lung Sq CC	FOXP1; MET; KMT2D; ERBB2; TP53; KMT2C
989133-093-R-J1	NSCLC	CDKN2A
LG0703-F948	NSCLC	EGFR L858R; KIT R49H; TP53
LG0567-F671	NSCLC NOS	KRAS G12C; TP53 R273C
349418-098-R	NSCLC NOS	ARID1A; BRAF V600E; GPS2; ERF; FAM58A; AR
556581-035-R-J1	Ovarian Ca	PIK3CA
SK-OV-3	Ovarian Ca	PIK3CA; TP53; APC; FBXW7; MSI high
292921-168-R-J2	Pancreatic Ca	MSH2; SETD2; RAD50; DNMT3A; APC; KRAS G12D
485368-065-R4-J2	Pancreatic Ca	KRAS G12V; ARID1A
521955-158-R7-J2	Pancreatic Ca	KRAS G12D; ATR; GNAQ
885724-159-R-J1	Pancreatic Ca	KRAS G12V; BRCA2; ERBB2; SMAD4;
521955-158-R6-J3	Pancreatic Ca	KRAS G12D; ATR; GNAQ
521955-158-R2-J5	Pancreatic Ca	KRAS G12D; ATR; GNAQ
K24384-001-R	Pancreatic Ca	KRAS G12V; TP53 Y238C
323965-272-R-J2	Pancreatic Ca	KRAS G12C
DU-145	Prostate Ca	CDKN2A; RB1; STK11
PC-3	Prostate Ca	Androgen independent
541946-237-B-J1	SCLC	RB1 loss of function; HDAC4
327498-153-R-J2	Uterine Sarcoma	ARID1A; PTEN del; CCND1; PARP1; MET; KRAS G12C; SMARCA4

**Table 3 •:** Growth media and number of tumor cells, endothelial cells, and human mesenchymal stem cells plated per well to form the tested mct-spheroids for response to ipatasertib alone and in simultaneous combination with the compounds in Table 1. Patient-derived cell lines are from the NCI PDMR (https://pdmr.cancer.gov/ [cited 2025 May 10]).

Malignant cell line	Malignant cell growth medium	Malignant cells per well	HUVECs per well	hMSCs per well
171881-019-R-J1	Complete DMEM/F12 Media-Y	2500	1042	625
MDA-MB-231	RPMI 1640/10% FBS	625	260	156
MDA-MB-468	RPMI 1640/10% FBS	625	260	156
Hs578T	RPMI 1640/10% FBS	1250	521	313
MCF7	RPMI 1640/10% FBS	313	130	78
CN0375-F725	Complete DMEM/F12 Media-Y	2500	1042	625
616215-338-R-J1	Complete DMEM/F12 Media-Y	625	260	156
439559-082-T-J2	Complete DMEM/F12 Media-Y	2500	1042	625
463943-066-T-J2	Complete DMEM/F12 Media-Y	2500	1042	625
519858-162-T-J1	Complete DMEM/F12 Media-Y	2500	1042	625
762968-020-R-J2	Complete DMEM/F12 Media-Y	2500	1042	625
817829-284-R-J1	Complete DMEM/F12 Media-Y	625	260	156
825966-067-R-J1	Complete DMEM/F12 Media-Y	1250	521	313
254851-301-R-J1	Complete DMEM/F12 Media-Y	2500	1042	625
276233-004-R-J1	Complete DMEM/F12 Media-Y	2500	1042	625
361931-004-R-J1	Complete DMEM/F12 Media-Y	1250	521	313
282377-053-R-J1	Complete DMEM/F12 Media-Y	625	260	156
186277-243-T-J2	Complete DMEM/F12 Media-Y	1250	521	313
931267-113-T-J1	Complete DMEM/F12 Media-Y	1250	521	313
253994-281-T-J1	Complete DMEM/F12 Media-Y	5000	2083	1250
832693-133-R-J1	Complete DMEM/F12 Media-Y	1250	521	313
845751-090-R-J2	Complete DMEM/F12 Media-Y	2500	1042	625
958767-090-R-J1	Complete DMEM/F12 Media-Y	625	260	156
128128-338-R-J1	Complete DMEM/F12 Media-Y	625	260	156
156681-154-R-J1	Complete DMEM/F12 Media-Y	1250	521	313
MALME-3M	RPMI 1640/10% FBS	625	260	156
SK-MEL-2	RPMI 1640/10% FBS	625	260	156
299254-011-R-J1	Complete DMEM/F12 Media-Y	1250	521	313
138582-337-R-J1	Complete DMEM/F12 Media-Y	5000	2083	1250
417821-307-R-J1	Complete DMEM/F12 Media-Y	2500	1042	625
989133-093-R-J1	Complete DMEM/F12 Media-Y	313	130	78
LG0703-F948	Complete DMEM/F12 Media-Y	625	260	156
LG0567-F671	Complete DMEM/F12 Media-Y	2500	1042	625
349418-098-R	Complete DMEM/F12 Media-Y	313	130	78
556581-035-R-J1	Complete DMEM/F12 Media-Y	2500	1042	625
SK-OV-3	RPMI 1640/10% FBS	313	130	78
292921-168-R-J2	Complete DMEM/F12 Media-Y	625	260	156
485368-065-R4-J2	Complete DMEM/F12 Media-Y	2500	1042	625
521955-158-R7-J2	Complete DMEM/F12 Media-Y	1250	521	313
885724-159-R-J1	Complete DMEM/F12 Media-Y	2500	1042	625
521955-158-R6-J3	Complete DMEM/F12 Media-Y	1250	521	313
521955-158-R2-J5	Complete DMEM/F12 Media-Y	625	260	156
K24384-001-R	Complete DMEM/F12 Media-Y	625	260	156
323965-272-R-J2	Complete DMEM/F12 Media-Y	1250	521	313
DU-145	RPMI 1640/10% FBS	1250	521	313
PC-3	RPMI 1640/10% FBS	625	260	156
541946-237-B-J1	Complete DMEM/F12 Media-Y	2500	1042	625
327498-153-R-J2	Complete DMEM/F12 Media-Y	625	260	156

## Data Availability

All data are accessible via the PubChem BioAssay public database (https://pubchem.ncbi.nlm.nih.gov/#query= [cited 2025 May 10]).
