# Supplementary material for: Targeted therapy combinations with ipatasertib in multi-cell type 3D tumor spheroid models
Source: Acad Oncol. Author manuscript; Available in PMC 2025 Aug 4. (PMC12320941; doi:10.20935/acadonco7726)
Supplement: Supplementary Material [file NIHMS2092104-supplement-Supplementary_Material.pdf]

Supplemental Table S1. Drugs and investigational agents used in this study. The agents included four PI3K inhibitors (*top*) that were each combined with molecular targeted agents (*bottom*). If available at the time of this study, the clinical C_{max} is listed.

**PI3K Inhibitors**	**Clinical C_{max}**	**Molecular Target(s)**
inavolisib	0.17 µM	PI3Kα
alpelisib	5.6 µM	PI3Kα
duvelisib	3.6 µM	PI3Kδ/γ
copanlisib	0.964 µM	pan-PI3K
**Combination Agents**	**Clinical C_{max}**	**Molecular Target(s)**
abemaciclib	0.59 µM	CDK4/6
palbociclib	0.101 µM	CDK4/6
SY5609	*NA*	CDK7
selumetinib	3.3 µM	MEK
tovorafenib (DAY101)	*NA*	RAF (type 2)
vemurafenib	127 µM	BRAF V600E
ravoxertinib	*NA*	ERK1/2
sotorasib	9.3 µM	KRAS G12C
MRTX1133	*NA*	KRAS G12D
sapanisertib	0.08 µM	mTORC1/2
afuresertib	*NA*	AKT
ipatasertib	*NA*	AKT
venetoclax	4.48 µM	BCL2
enzalutamide	35.7 µM	androgen receptor
darolutamide	*NA*	androgen receptor
talazoparib	0.043 µM	PARP

NA, clinical C_{max} unknown, highest concentration tested was 10 µM.

Supplemental Table S2. The malignant cell lines grown as multi-cell type tumor spheroids for this study. The names of both patient-derived (https://pdmr.cancer.gov/) and established cell lines are listed along with the tumor type they were derived from and alterations classified as oncogenic by OncoKB.

Malignant Cell Line	Tumor Type	PI3K Status	Additional Oncogenic Alterations
186277-243-T-J2	Colon	PIK3CA C420R	KRAS G12D; DNMT3A R882H
233499-124-R-J3	Colon	-	KRAS A146T; MTOR T1977I
361931-004-R-J1	Colon	PIK3CA H1047R	BRAF V600E; FBXW7 R465C
435261-313-R-J1	Colon	PIK3CA E542K	KRAS G12V; copy number amplification of AKT3, MCL1, and MDM4
616215-338-R-J1	Colon	-	BRAF V600E; KRAS G12S
624824-186-R-J2	Colon	PIK3CA H1047R	BRAF V600E; copy number amplification of MYC
624824-186-R-J3	Colon	PIK3CA H1047R	BRAF V600E; copy number amplification of MYC
817829-284-R-J1	Colon	-	BRAF V600E; EGFR A611T
958667-338-R-J2	Colon	PIK3CA E542K	KRAS G13D
967376-340-R-J2	Colon	PIK3CA R108H	-
996289-038-R-J1	Colon	-	KRAS G12D
K98450-129-R-J1	Colon	-	KRAS G12C; copy number amplification of CCNE1, deletion of CDKN2A and CDKN2B
931267-113-T-J1	Colorectal	PIK3CA E545K	KRAS G12D; FBXW7 R465C
475296-252-R-J1	Rectal	PIK3CA E545K	KRAS G12D; copy number amplification of MYC
945468-187-T-J2	Rectal	PIK3CA Q546K	VHL R200W
945468-187-T-J3	Rectal	PIK3CA Q546K	VHL R200W
935938-334-R-J1	Small intestine	PIK3CA R88Q	-
252836-082-R-J2	Head and neck	-	Copy number amplification of FGFR1
354836-022-R-J1	Head and neck	PIK3CA E545A	PTEN deletion; EGFR E758Q
171881-019-R-J1	Breast	PIK3CA E545Q, H1047R	Copy number amplification of PAK1
MDA-MB-231	Triple negative breast	-	TP53 R280K; BRAF G464V; KRAS G13D; deletion of CDKN2A and CDKN2B
MDA-MB-468	Triple negative breast	-	TP53 R273H; PTEN X85_splice; copy number amplification of ERBB2 and EGFR
T-47D	Triple negative breast	PIK3CA H1047R	TP53 L194F; copy number amplification of PIK3CA
997726-040-R-J2	Lung	PIK3CA E545K	NFE2L2 G31A
313798-341-R-J2	Cervical	PIK3CA E545K	Copy number amplification of RICTOR
953936-095-R-J2	Cervical	PIK3CA E542K	-
648629-189-R-J1	Bladder	PIK3CA E542Q	Copy number amplification of CCNE1 and YAP1
855422-203-R-J1	Bladder	PIK3CA E545K	Deletion of CDKN2A and CDKN2B
883617-216-R-J1	Bladder	-	HRAS G12C; EGFR R222C; deletion of CDKN2A and CDKN2B

All data were obtained from the NCI Patient-Derived Models Repository (https://pdmr.cancer.gov) or cBioPortal (https://www.cbioportal.org/).

Supplemental Table S3. Multi-cell type tumor model cell plating densities per well in 384-well microplates.

Malignant Cell Line	Malignant Cells per well	HUVEC[a] per well	hMSC[b] per well
233499-124-R-J3	1250	521	313
361931-004-R-J1	1250	521	313
435261-313-R-J1	1250	521	313
616215-338-R-J1	625	260	156
624824-186-R-J2	2500	1042	625
624824-186-R-J3	2500	1042	625
817829-284-R-J1	625	260	156
958667-338-R-J2	2500	1042	625
967376-340-R-J2	2500	1042	625
996289-038-R-J1	2500	1042	625
K98450-129-R-J1	2500	1042	625
931267-113-T-J1	1250	521	313
475296-252-R-J1	5000	2083	1250
945468-187-T-J2	1250	521	313
945468-187-T-J3	625	260	156
935938-334-R-J1	625	260	156
252836-082-R-J2	625	260	156
354836-022-R-J1	2500	1042	625
171881-019-R-J1	2500	1042	625
MDA-MB-231	625	260	156
MDA-MB-468	625	260	156
T-47D	625	260	156
997726-040-R-J2	1250	521	313
313798-341-R-J2	2500	1042	625
953936-095-R-J2	1250	521	313
648629-189-R-J1	625	260	156
855422-203-R-J1	313	130	78
883617-216-R-J1	625	260	156

[a] human umbilical vein endothelial cells

[b] human mesenchymal stem cells

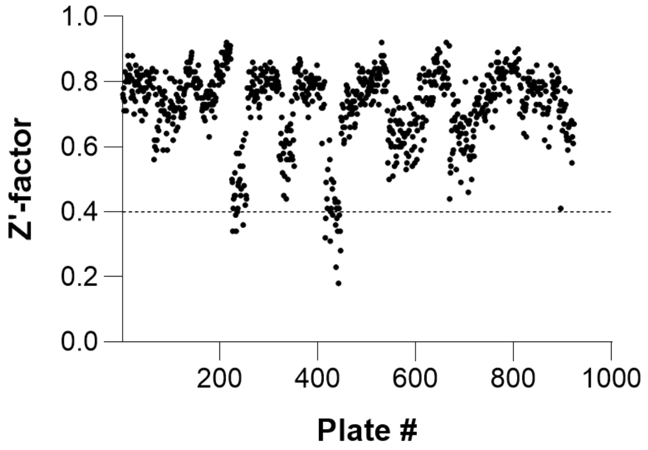

Supplemental Figure S1. Z'-factors from the drug combination screen. The Z'-factor from each of the 384-well plates (*n* = 923) are shown as solid dots. Two plates with Z'-factor values below the x-axis are not displayed. The dashed line represents the 0.4 threshold.

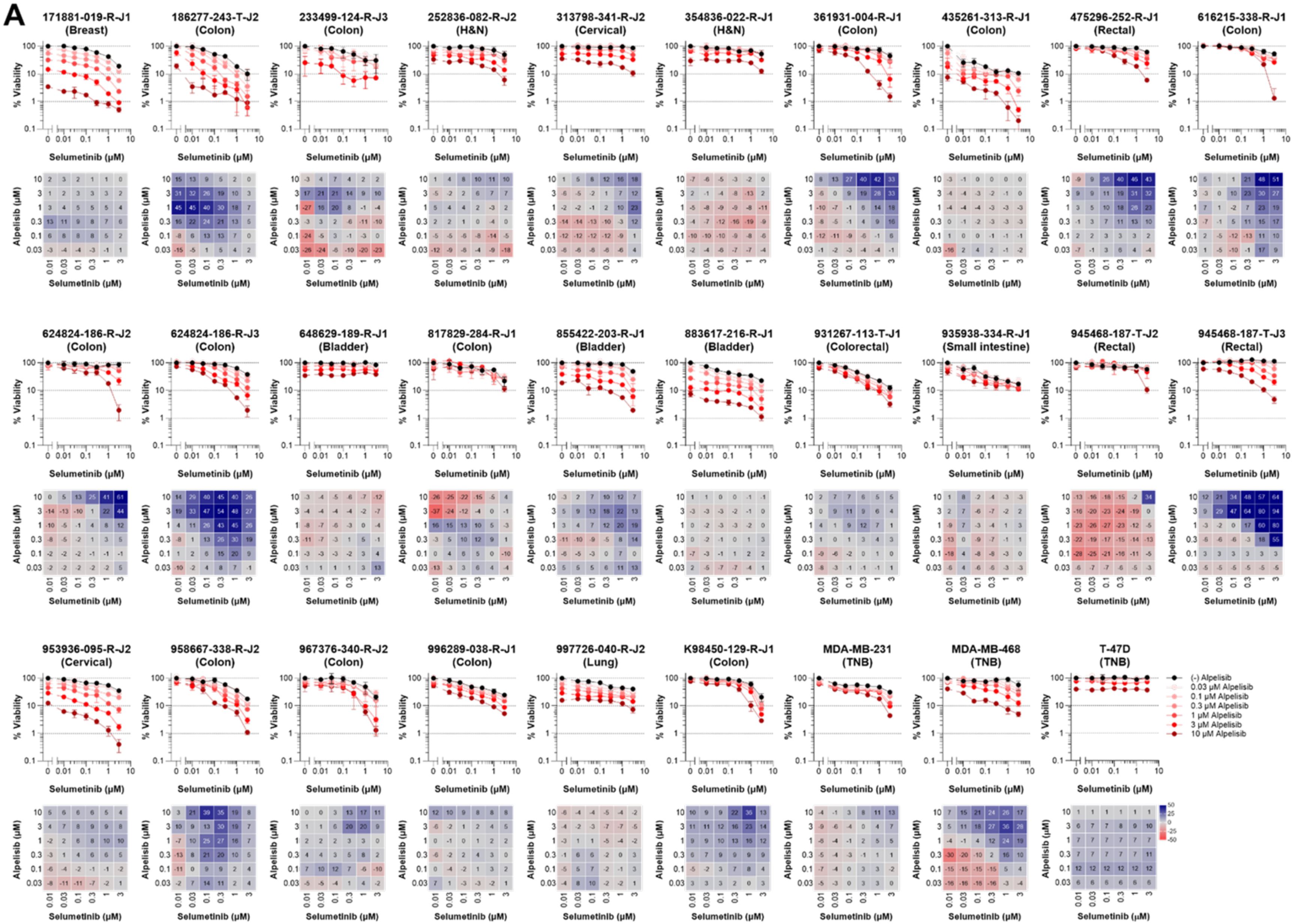

A
171881-019-R-J1 (Breast)
186277-243-T-J2 (Colon)
233499-124-R-J3 (Colon)
252836-082-R-J2 (H&N)
313798-341-R-J2 (Cervical)
354836-022-R-J1 (H&N)
361931-004-R-J1 (Colon)
435261-313-R-J1 (Colon)
475296-252-R-J1 (Rectal)
616215-338-R-J1 (Colon)
624824-186-R-J2 (Colon)
624824-186-R-J3 (Colon)
648629-189-R-J1 (Bladder)
817829-284-R-J1 (Colon)
855422-203-R-J1 (Bladder)
883617-216-R-J1 (Bladder)
931267-113-T-J1 (Colorectal)
935938-334-R-J1 (Small intestine)
945468-187-T-J2 (Rectal)
945468-187-T-J3 (Rectal)
953936-095-R-J2 (Cervical)
958667-338-R-J2 (Colon)
967376-340-R-J2 (Colon)
996289-038-R-J1 (Colon)
997726-040-R-J2 (Lung)
K98450-129-R-J1 (Colon)
MDA-MB-231 (TNB)
MDA-MB-468 (TNB)
T-47D (TNB)
% Viability
Selumetinib (µM)
Alpelisib (µM)
(-) Alpelisib
0.03 µM Alpelisib
0.1 µM Alpelisib
0.3 µM Alpelisib
1 µM Alpelisib
3 µM Alpelisib
10 µM Alpelisib

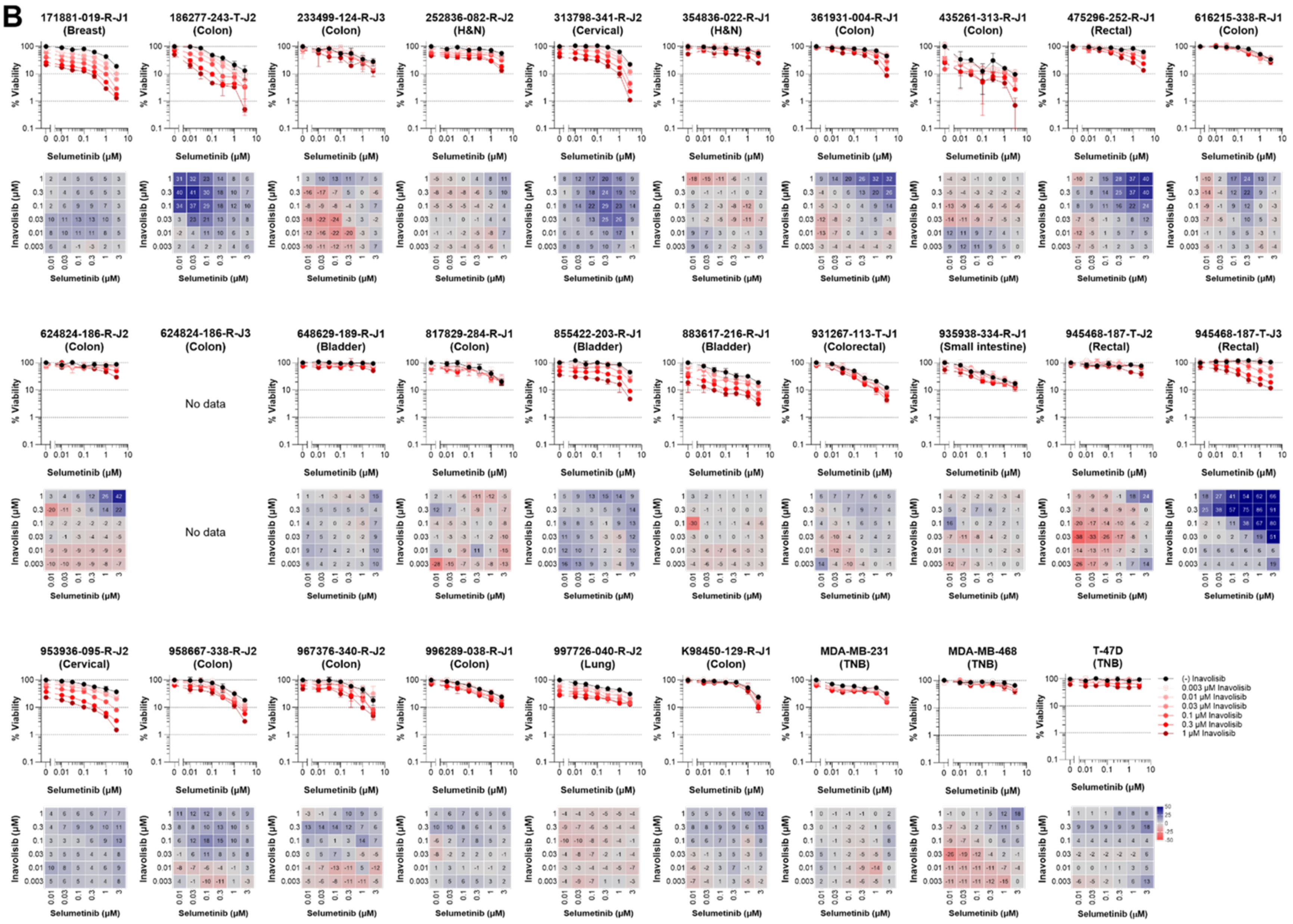
B
171881-019-R-J1 (Breast)
186277-243-T-J2 (Colon)
233499-124-R-J3 (Colon)
252836-082-R-J2 (H&N)
313798-341-R-J2 (Cervical)
354836-022-R-J1 (H&N)
361931-004-R-J1 (Colon)
435261-313-R-J1 (Colon)
475296-252-R-J1 (Rectal)
616215-338-R-J1 (Colon)
624824-186-R-J2 (Colon)
624824-186-R-J3 (Colon)
No data
No data
648629-189-R-J1 (Bladder)
817829-284-R-J1 (Colon)
855422-203-R-J1 (Bladder)
883617-216-R-J1 (Bladder)
931267-113-T-J1 (Colorectal)
935938-334-R-J1 (Small intestine)
945468-187-T-J2 (Rectal)
945468-187-T-J3 (Rectal)
953936-095-R-J2 (Cervical)
958667-338-R-J2 (Colon)
967376-340-R-J2 (Colon)
996289-038-R-J1 (Colon)
997726-040-R-J2 (Lung)
K98450-129-R-J1 (Colon)
MDA-MB-231 (TNB)
MDA-MB-468 (TNB)
T-47D (TNB)
% Viability
Selumetinib (µM)
Inavolisib (µM)
(-) Inavolisib
0.003 µM Inavolisib
0.01 µM Inavolisib
0.03 µM Inavolisib
0.1 µM Inavolisib
0.3 µM Inavolisib
1 µM Inavolisib

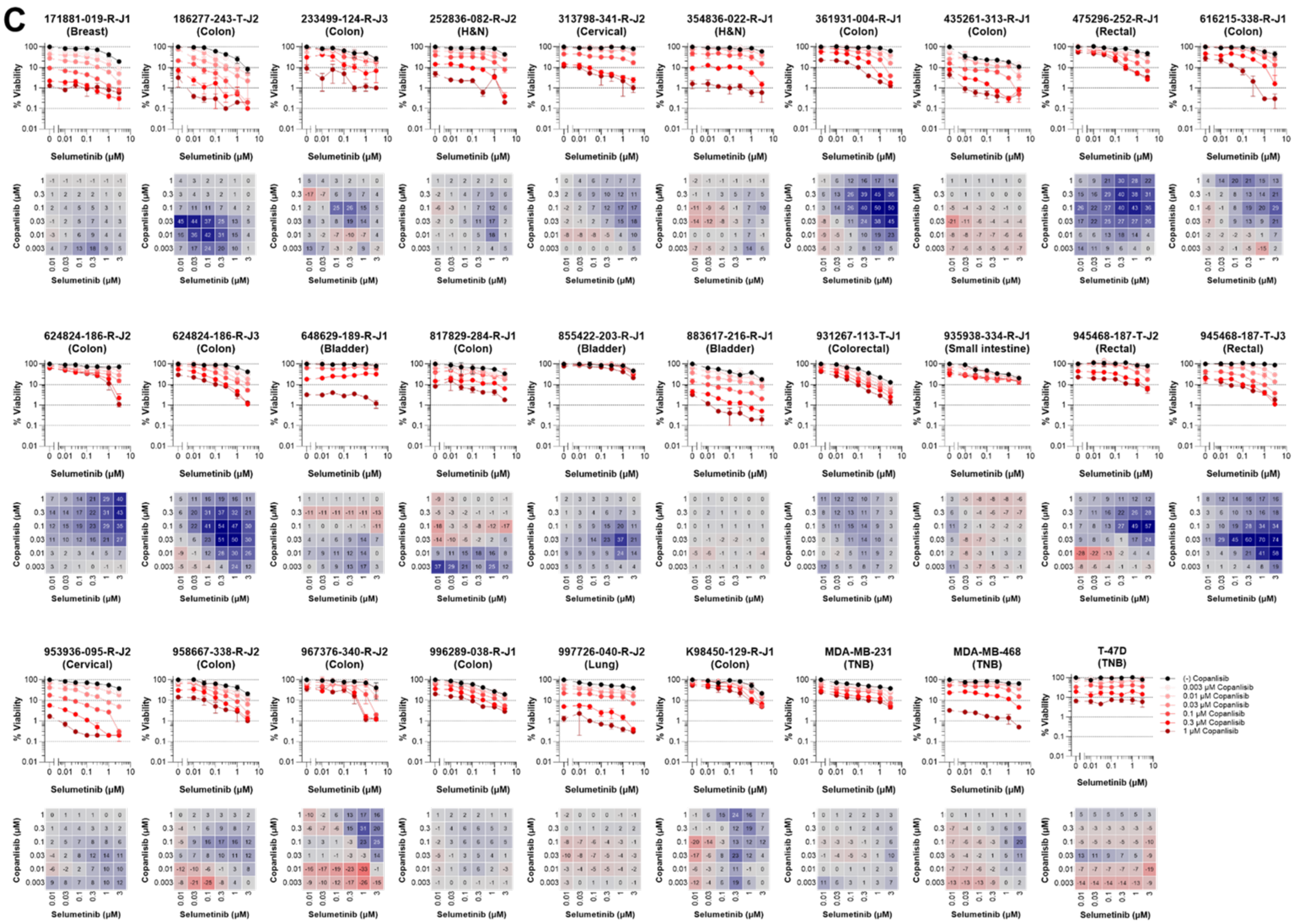
C
171881-019-R-J1 (Breast)
186277-243-T-J2 (Colon)
233499-124-R-J3 (Colon)
252836-082-R-J2 (H&N)
313798-341-R-J2 (Cervical)
354836-022-R-J1 (H&N)
361931-004-R-J1 (Colon)
435261-313-R-J1 (Colon)
475296-252-R-J1 (Rectal)
616215-338-R-J1 (Colon)
624824-186-R-J2 (Colon)
624824-186-R-J3 (Colon)
648629-189-R-J1 (Bladder)
817829-284-R-J1 (Colon)
855422-203-R-J1 (Bladder)
883617-216-R-J1 (Bladder)
931267-113-T-J1 (Colorectal)
935938-334-R-J1 (Small intestine)
945468-187-T-J2 (Rectal)
945468-187-T-J3 (Rectal)
953936-095-R-J2 (Cervical)
958667-338-R-J2 (Colon)
967376-340-R-J2 (Colon)
996289-038-R-J1 (Colon)
997726-040-R-J2 (Lung)
K98450-129-R-J1 (Colon)
MDA-MB-231 (TNB)
MDA-MB-468 (TNB)
T-47D (TNB)
% Viability
Selumetinib (μM)
Copanlisib (μM)
(-) Copanlisib
0.003 μM Copanlisib
0.01 μM Copanlisib
0.03 μM Copanlisib
0.1 μM Copanlisib
0.3 μM Copanlisib
1 μM Copanlisib

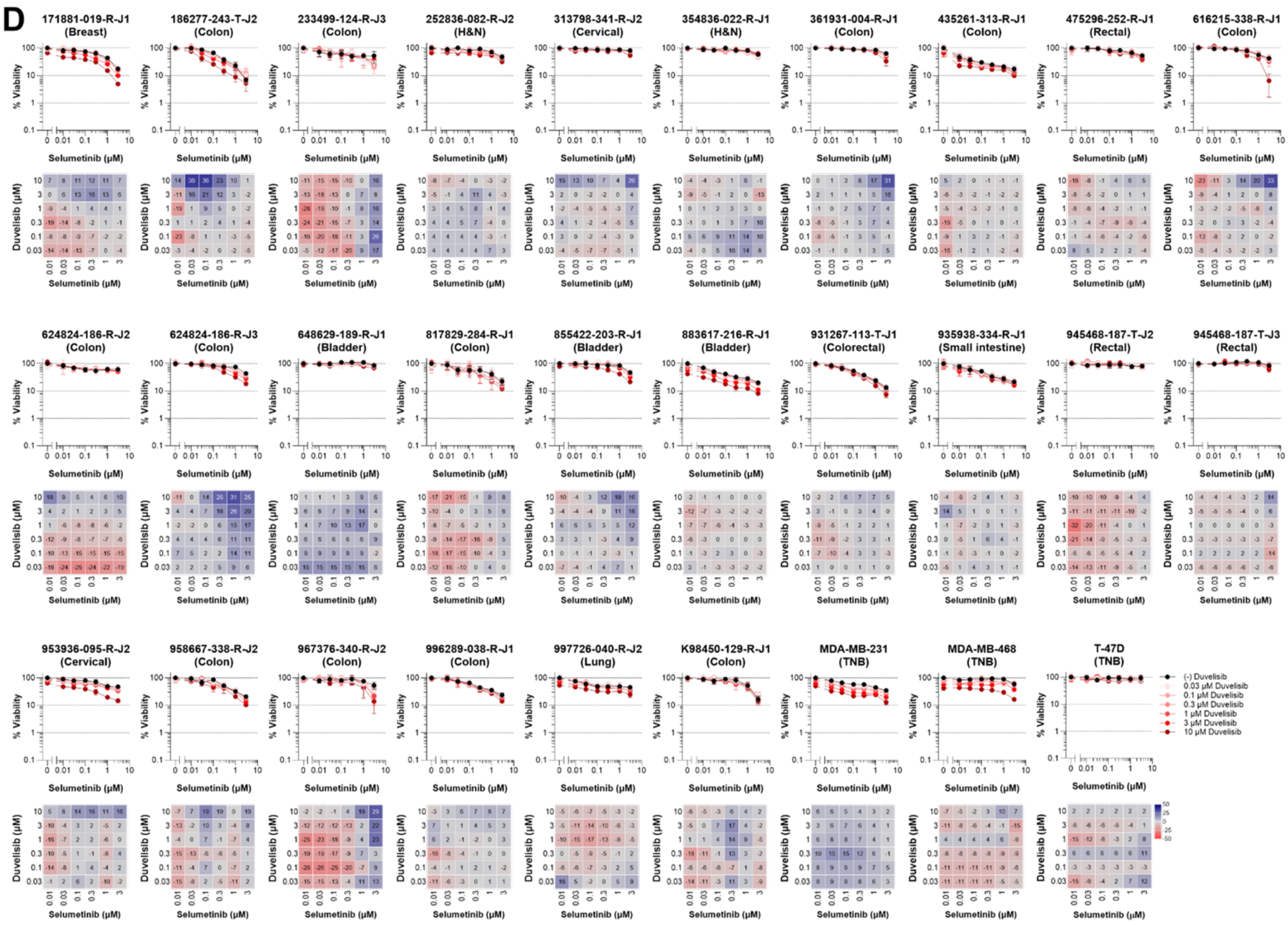
D
171881-019-R-J1 (Breast)
186277-243-T-J2 (Colon)
233499-124-R-J3 (Colon)
252836-082-R-J2 (H&N)
313798-341-R-J2 (Cervical)
354836-022-R-J1 (H&N)
361931-004-R-J1 (Colon)
435261-313-R-J1 (Colon)
475296-252-R-J1 (Rectal)
616215-338-R-J1 (Colon)
624824-186-R-J2 (Colon)
624824-186-R-J3 (Colon)
648629-189-R-J1 (Bladder)
817829-284-R-J1 (Colon)
855422-203-R-J1 (Bladder)
883617-216-R-J1 (Bladder)
931267-113-T-J1 (Colorectal)
935938-334-R-J1 (Small intestine)
945468-187-T-J2 (Rectal)
945468-187-T-J3 (Rectal)
953936-095-R-J2 (Cervical)
958667-338-R-J2 (Colon)
967376-340-R-J2 (Colon)
996289-038-R-J1 (Colon)
997726-040-R-J2 (Lung)
K98450-129-R-J1 (Colon)
MDA-MB-231 (TNB)
MDA-MB-468 (TNB)
T-47D (TNB)
% Viability
Selumetinib (μM)
Duvelisib (μM)
(-) Duvelisib
0.03 μM Duvelisib
0.1 μM Duvelisib
0.3 μM Duvelisib
1 μM Duvelisib
3 μM Duvelisib
10 μM Duvelisib

Supplemental Figure S2. Combination activity for selumetinib with a PI3K inhibitor. Concentration-response graphs (*top*, mean ± SD, $n = 3$ technical replicates) and Bliss independence scores across the concentration matrix (*bottom*, mean of $n = 3$ technical replicates) are shown as numerical values and heat maps (blue indicates synergy, gray indicates additivity, and red indicates antagonism). Data are presented, if available, from twenty-nine malignant cell lines grown as multi-cell type tumor spheroids and treated with selumetinib in combination with (A) alpelisib, (B) inavolisib, (C) copanlisib, or (D) duvelisib. The tumor model name and type are indicated above each set of graphs.

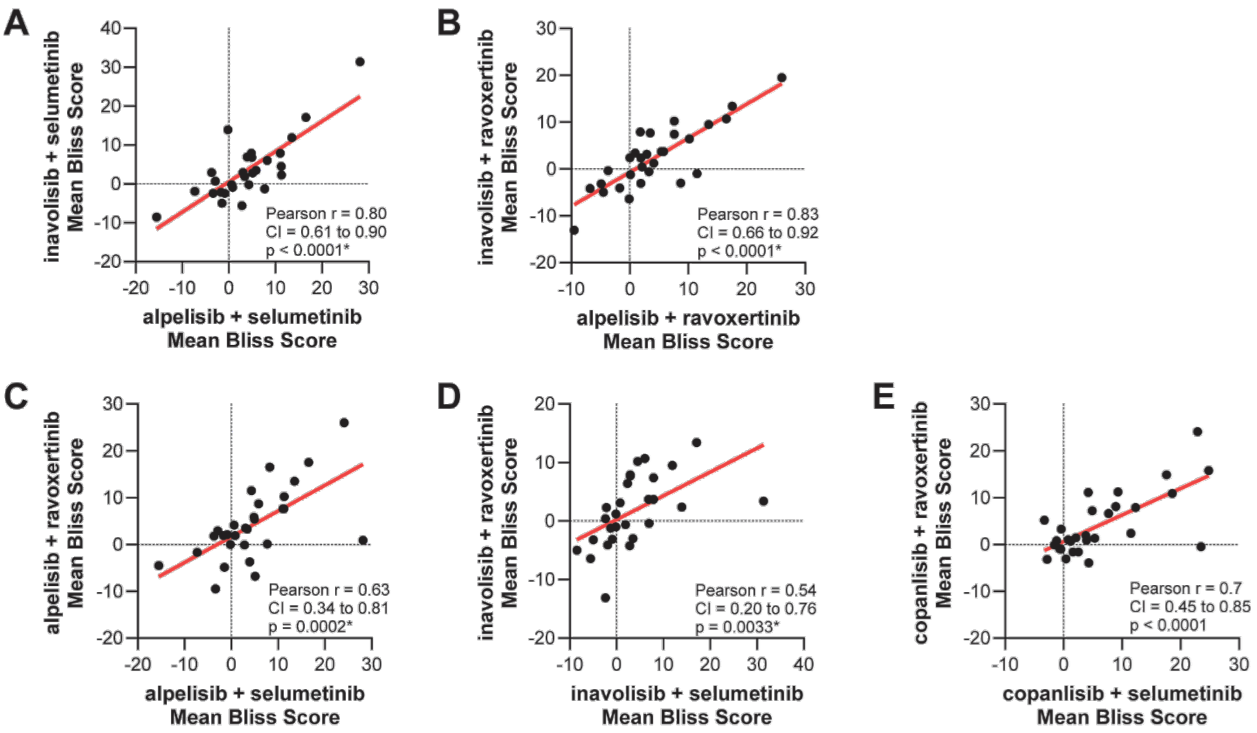

Supplemental Figure S3. Mean Bliss score correlations for alpelisib, inavolisib, or copanlisib in combination with selumetinib or ravoxertinib. Scatter plots depict significant correlations between the mean Bliss scores from combinations of (A) alpelisib or inavolisib with selumetinib, (B) alpelisib or inavolisib with ravoxertinib, (C) alpelisib with selumetinib or ravoxertinib, (D) inavolisib with selumetinib or ravoxertinib, and (E) copanlisib with selumetinib or ravoxertinib. Pearson correlation coefficients (r), confidence intervals (CI), and p-values are shown, with statistical significance indicated by an asterisk (*).

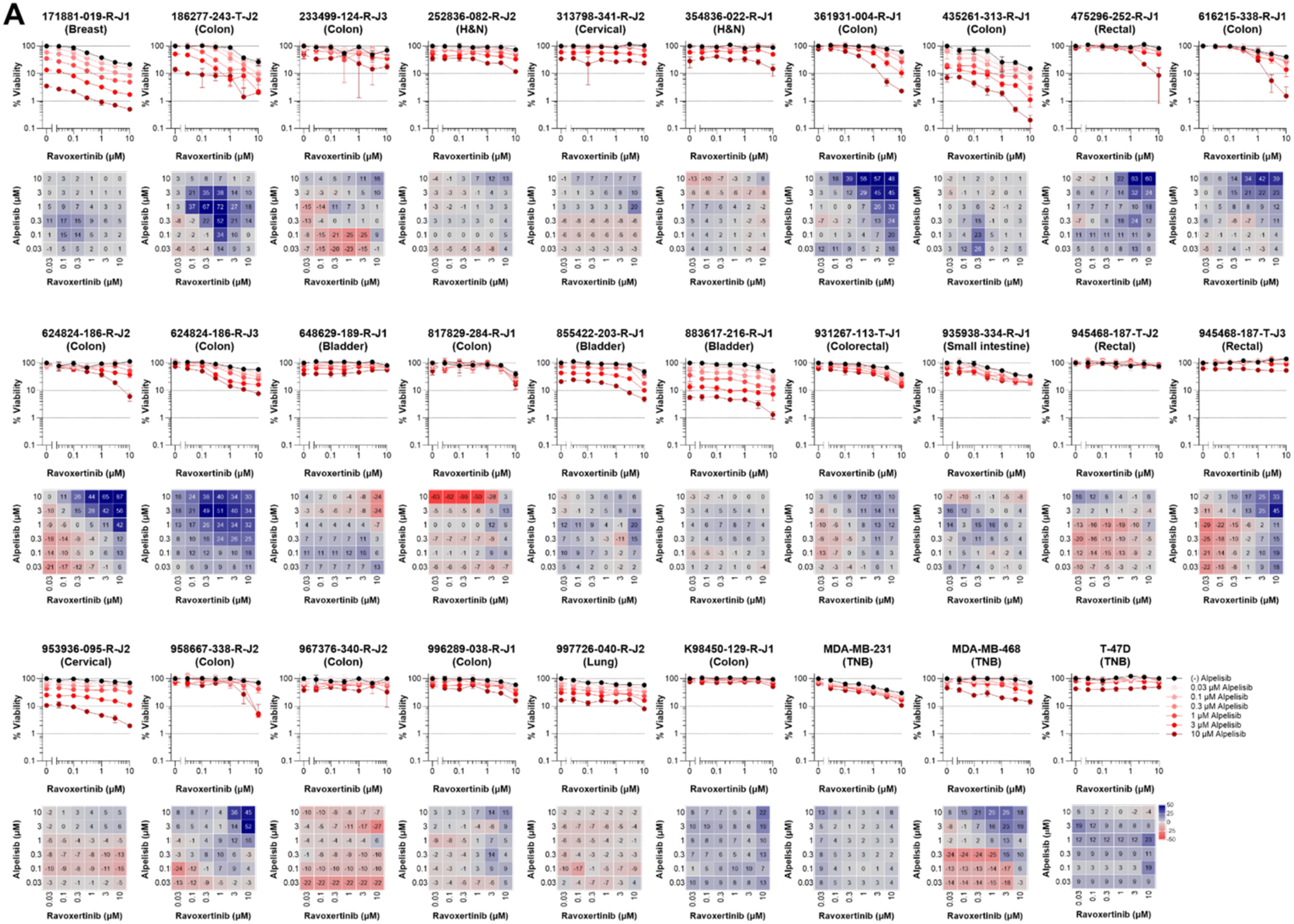
A
171881-019-R-J1 (Breast)
186277-243-T-J2 (Colon)
233499-124-R-J3 (Colon)
252836-082-R-J2 (H&N)
313798-341-R-J2 (Cervical)
354836-022-R-J1 (H&N)
361931-004-R-J1 (Colon)
435261-313-R-J1 (Colon)
475296-252-R-J1 (Rectal)
616215-338-R-J1 (Colon)
624824-186-R-J2 (Colon)
624824-186-R-J3 (Colon)
648629-189-R-J1 (Bladder)
817829-284-R-J1 (Colon)
855422-203-R-J1 (Bladder)
883617-216-R-J1 (Bladder)
931267-113-T-J1 (Colorectal)
935938-334-R-J1 (Small intestine)
945468-187-T-J2 (Rectal)
945468-187-T-J3 (Rectal)
953936-095-R-J2 (Cervical)
958667-338-R-J2 (Colon)
967376-340-R-J2 (Colon)
996289-038-R-J1 (Colon)
997726-040-R-J2 (Lung)
K98450-129-R-J1 (Colon)
MDA-MB-231 (TNB)
MDA-MB-468 (TNB)
T-47D (TNB)
% Viability
Ravoxertinib (µM)
Alpelisib (µM)
(-) Alpelisib
0.03 µM Alpelisib
0.1 µM Alpelisib
0.3 µM Alpelisib
1 µM Alpelisib
3 µM Alpelisib
10 µM Alpelisib

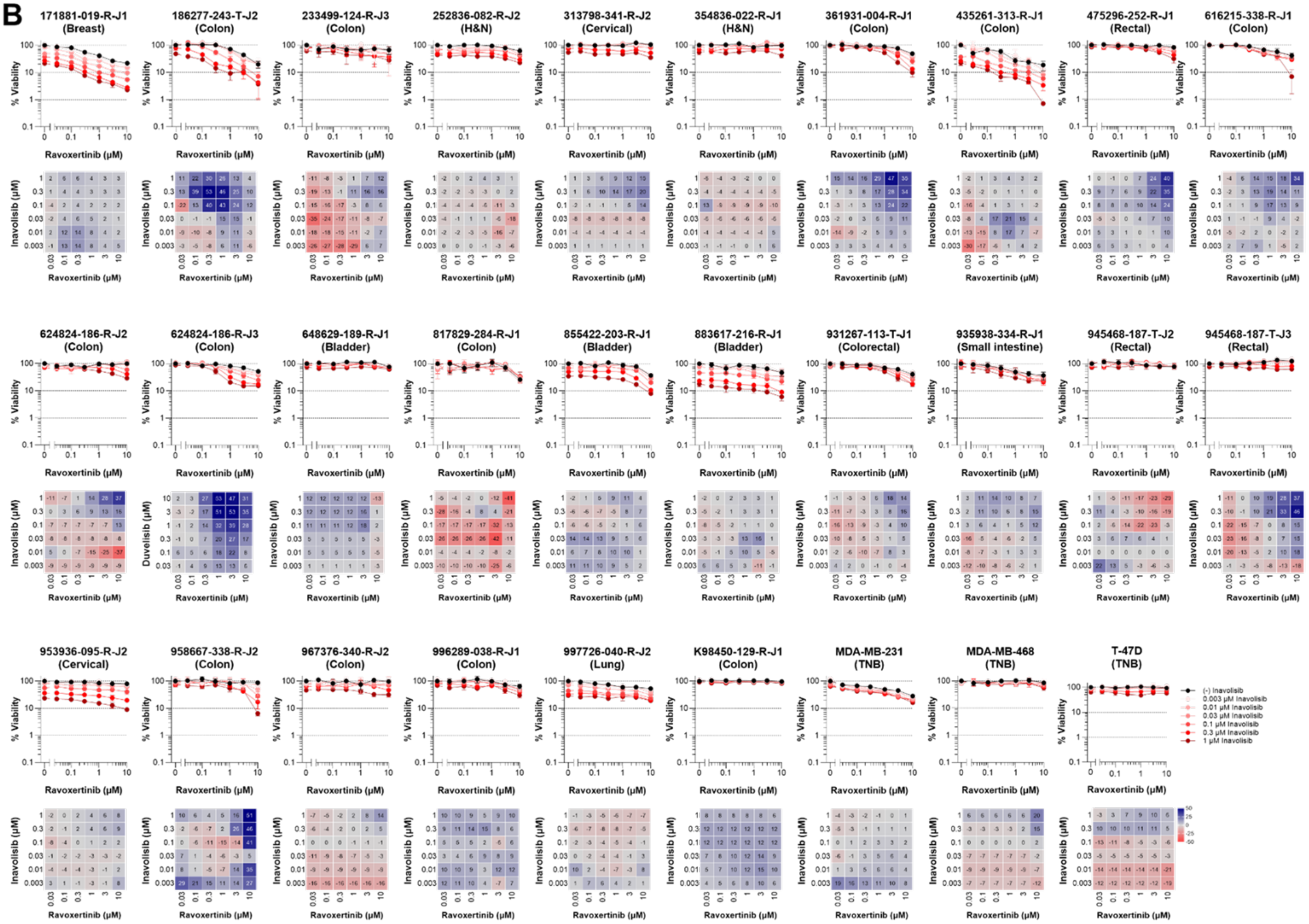
B
171881-019-R-J1 (Breast)
186277-243-T-J2 (Colon)
233499-124-R-J3 (Colon)
252836-082-R-J2 (H&N)
313798-341-R-J2 (Cervical)
354836-022-R-J1 (H&N)
361931-004-R-J1 (Colon)
435261-313-R-J1 (Colon)
475296-252-R-J1 (Rectal)
616215-338-R-J1 (Colon)
624824-186-R-J2 (Colon)
624824-186-R-J3 (Colon)
648629-189-R-J1 (Bladder)
817829-284-R-J1 (Colon)
855422-203-R-J1 (Bladder)
883617-216-R-J1 (Bladder)
931267-113-T-J1 (Colorectal)
935938-334-R-J1 (Small intestine)
945468-187-T-J2 (Rectal)
945468-187-T-J3 (Rectal)
953936-095-R-J2 (Cervical)
958667-338-R-J2 (Colon)
967376-340-R-J2 (Colon)
996289-038-R-J1 (Colon)
997726-040-R-J2 (Lung)
K98450-129-R-J1 (Colon)
MDA-MB-231 (TNB)
MDA-MB-468 (TNB)
T-47D (TNB)
% Viability
Ravoxertinib (µM)
Inavolisib (µM)
Duvelisib (µM)
(-) Inavolisib
0.003 µM Inavolisib
0.01 µM Inavolisib
0.03 µM Inavolisib
0.1 µM Inavolisib
0.3 µM Inavolisib
1 µM Inavolisib

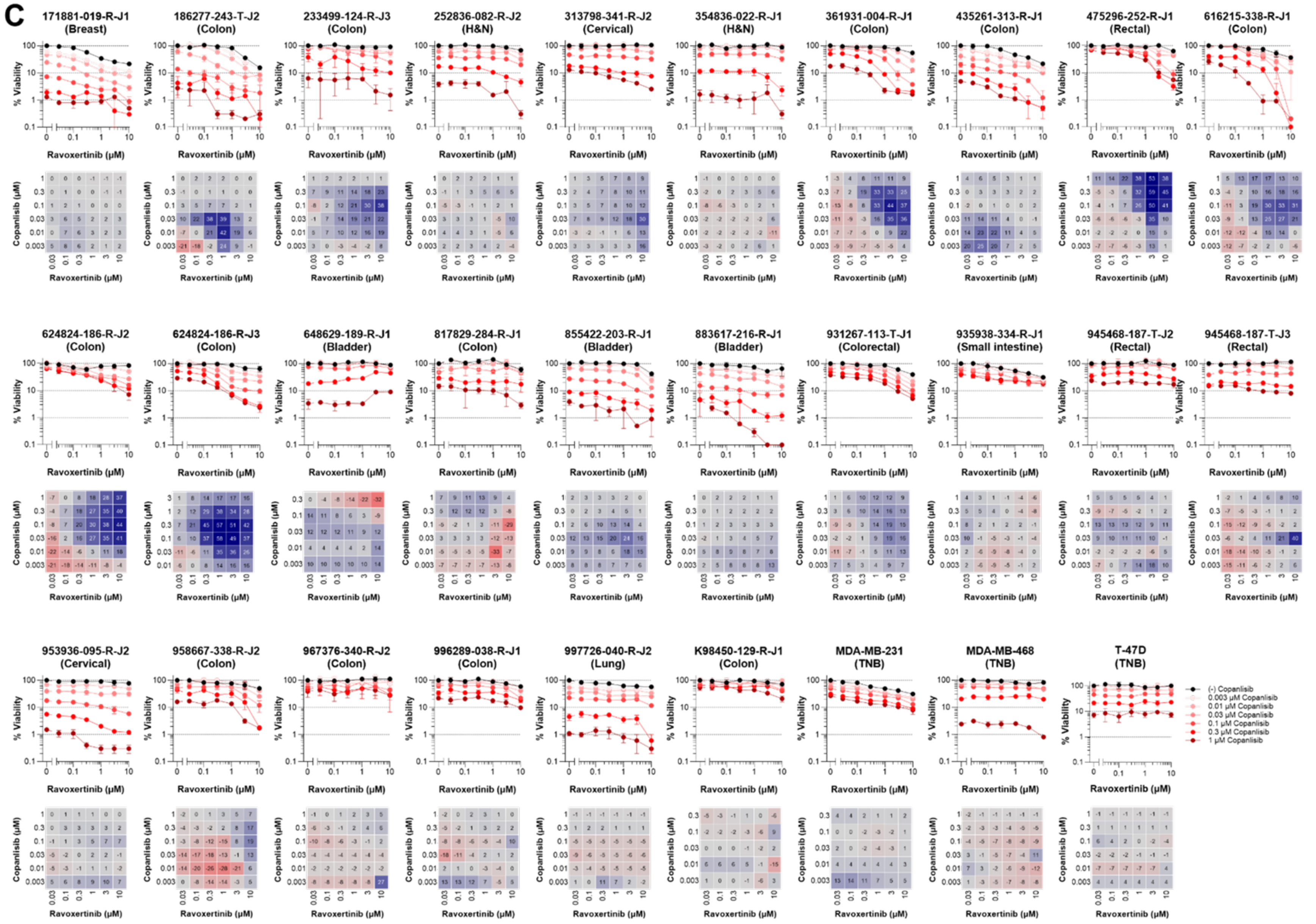
C
171881-019-R-J1 (Breast)
186277-243-T-J2 (Colon)
233499-124-R-J3 (Colon)
252836-082-R-J2 (H&N)
313798-341-R-J2 (Cervical)
354836-022-R-J1 (H&N)
361931-004-R-J1 (Colon)
435261-313-R-J1 (Colon)
475296-252-R-J1 (Rectal)
616215-338-R-J1 (Colon)
624824-186-R-J2 (Colon)
624824-186-R-J3 (Colon)
648629-189-R-J1 (Bladder)
817829-284-R-J1 (Colon)
855422-203-R-J1 (Bladder)
883617-216-R-J1 (Bladder)
931267-113-T-J1 (Colorectal)
935938-334-R-J1 (Small intestine)
945468-187-T-J2 (Rectal)
945468-187-T-J3 (Rectal)
953936-095-R-J2 (Cervical)
958667-338-R-J2 (Colon)
967376-340-R-J2 (Colon)
996289-038-R-J1 (Colon)
997726-040-R-J2 (Lung)
K98450-129-R-J1 (Colon)
MDA-MB-231 (TNB)
MDA-MB-468 (TNB)
T-47D (TNB)
% Viability
Ravoxertinib (μM)
Copanlisib (μM)
(-) Copanlisib
0.003 μM Copanlisib
0.01 μM Copanlisib
0.03 μM Copanlisib
0.1 μM Copanlisib
0.3 μM Copanlisib
1 μM Copanlisib

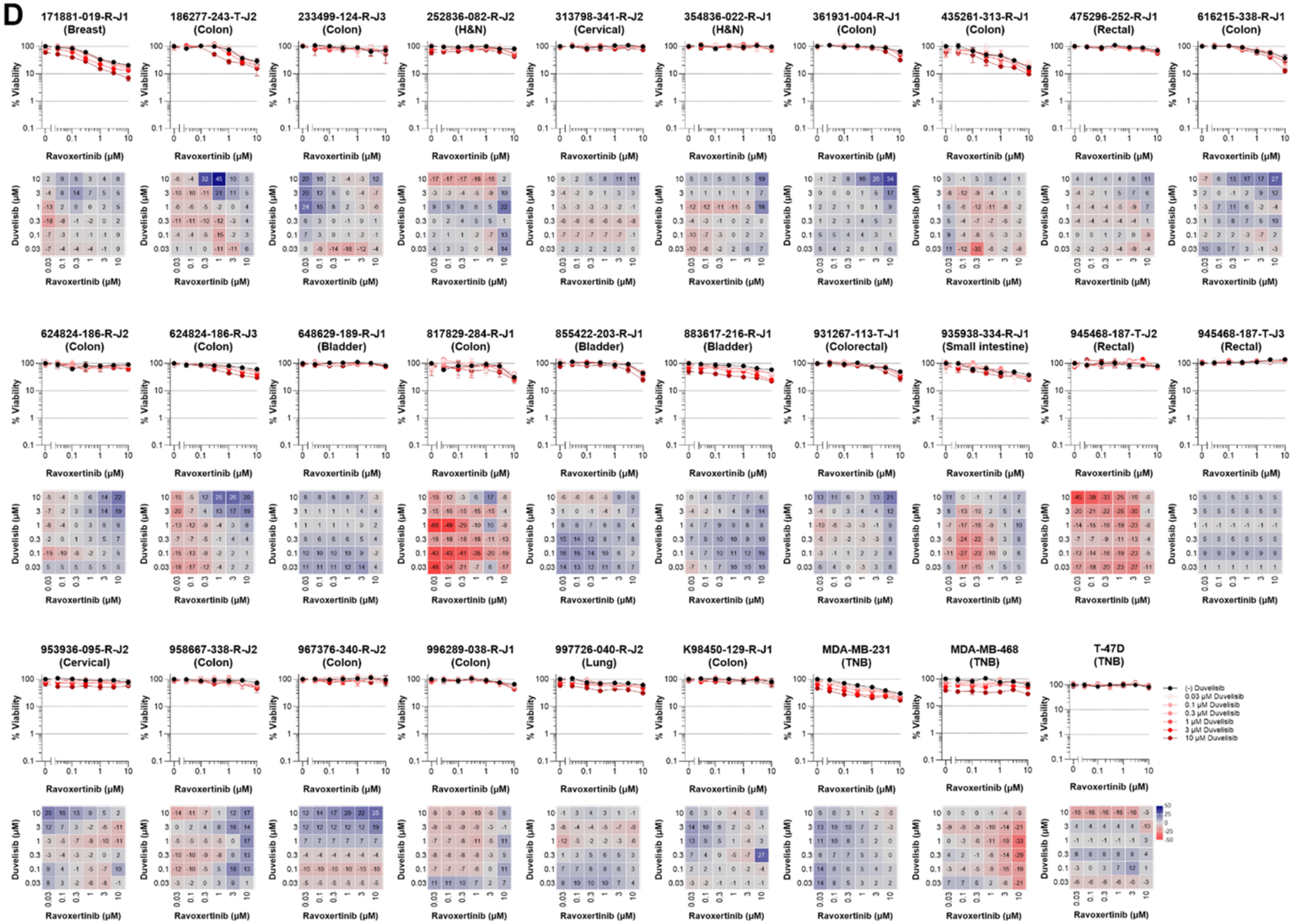
D
171881-019-R-J1 (Breast)
186277-243-T-J2 (Colon)
233499-124-R-J3 (Colon)
252836-082-R-J2 (H&N)
313798-341-R-J2 (Cervical)
354836-022-R-J1 (H&N)
361931-004-R-J1 (Colon)
435261-313-R-J1 (Colon)
475296-252-R-J1 (Rectal)
616215-338-R-J1 (Colon)
624824-186-R-J2 (Colon)
624824-186-R-J3 (Colon)
648629-189-R-J1 (Bladder)
817829-284-R-J1 (Colon)
855422-203-R-J1 (Bladder)
883617-216-R-J1 (Bladder)
931267-113-T-J1 (Colorectal)
935938-334-R-J1 (Small intestine)
945468-187-T-J2 (Rectal)
945468-187-T-J3 (Rectal)
953936-095-R-J2 (Cervical)
958667-338-R-J2 (Colon)
967376-340-R-J2 (Colon)
996289-038-R-J1 (Colon)
997726-040-R-J2 (Lung)
K98450-129-R-J1 (Colon)
MDA-MB-231 (TNB)
MDA-MB-468 (TNB)
T-47D (TNB)
% Viability
Ravoxertinib (µM)
Duvelisib (µM)
(-) Duvelisib
0.03 µM Duvelisib
0.1 µM Duvelisib
0.3 µM Duvelisib
1 µM Duvelisib
3 µM Duvelisib
10 µM Duvelisib

Supplemental Figure S4. Combination activity for ravoxertinib with a PI3K inhibitor. Concentration-response graphs (*top*, mean ± SD, *n* = 3 technical replicates) and Bliss independence scores across the concentration matrix (*bottom*, mean of *n* = 3 technical replicates) are shown as numerical values and heat maps (blue indicates synergy, gray indicates additivity, and red indicates antagonism). Data are presented, if available, from twenty-nine malignant cell lines grown as multi-cell type tumor spheroids and treated with ravoxertinib in combination with (A) alpelisib, (B) inavolisib, (C) copanlisib, or (D) duvelisib. The tumor model name and type are indicated above each set of graphs.

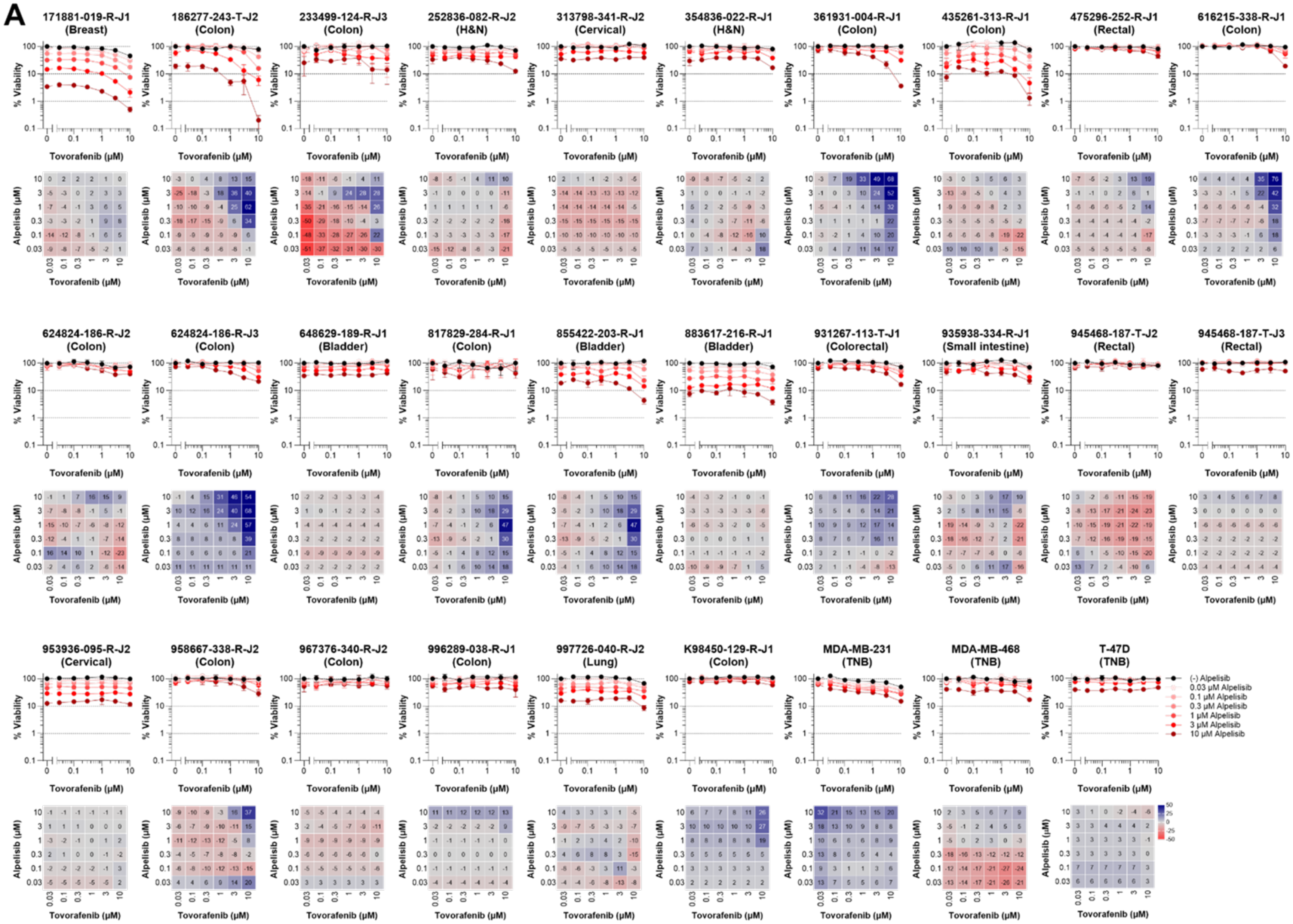
A
171881-019-R-J1 (Breast)
186277-243-T-J2 (Colon)
233499-124-R-J3 (Colon)
252836-082-R-J2 (H&N)
313798-341-R-J2 (Cervical)
354836-022-R-J1 (H&N)
361931-004-R-J1 (Colon)
435261-313-R-J1 (Colon)
475296-252-R-J1 (Rectal)
616215-338-R-J1 (Colon)
624824-186-R-J2 (Colon)
624824-186-R-J3 (Colon)
648629-189-R-J1 (Bladder)
817829-284-R-J1 (Colon)
855422-203-R-J1 (Bladder)
883617-216-R-J1 (Bladder)
931267-113-T-J1 (Colorectal)
935938-334-R-J1 (Small intestine)
945468-187-T-J2 (Rectal)
945468-187-T-J3 (Rectal)
953936-095-R-J2 (Cervical)
958667-338-R-J2 (Colon)
967376-340-R-J2 (Colon)
996289-038-R-J1 (Colon)
997726-040-R-J2 (Lung)
K98450-129-R-J1 (Colon)
MDA-MB-231 (TNB)
MDA-MB-468 (TNB)
T-47D (TNB)
% Viability
Tovorafenib (µM)
Alpelisib (µM)
(-) Alpelisib
0.03 µM Alpelisib
0.1 µM Alpelisib
0.3 µM Alpelisib
1 µM Alpelisib
3 µM Alpelisib
10 µM Alpelisib

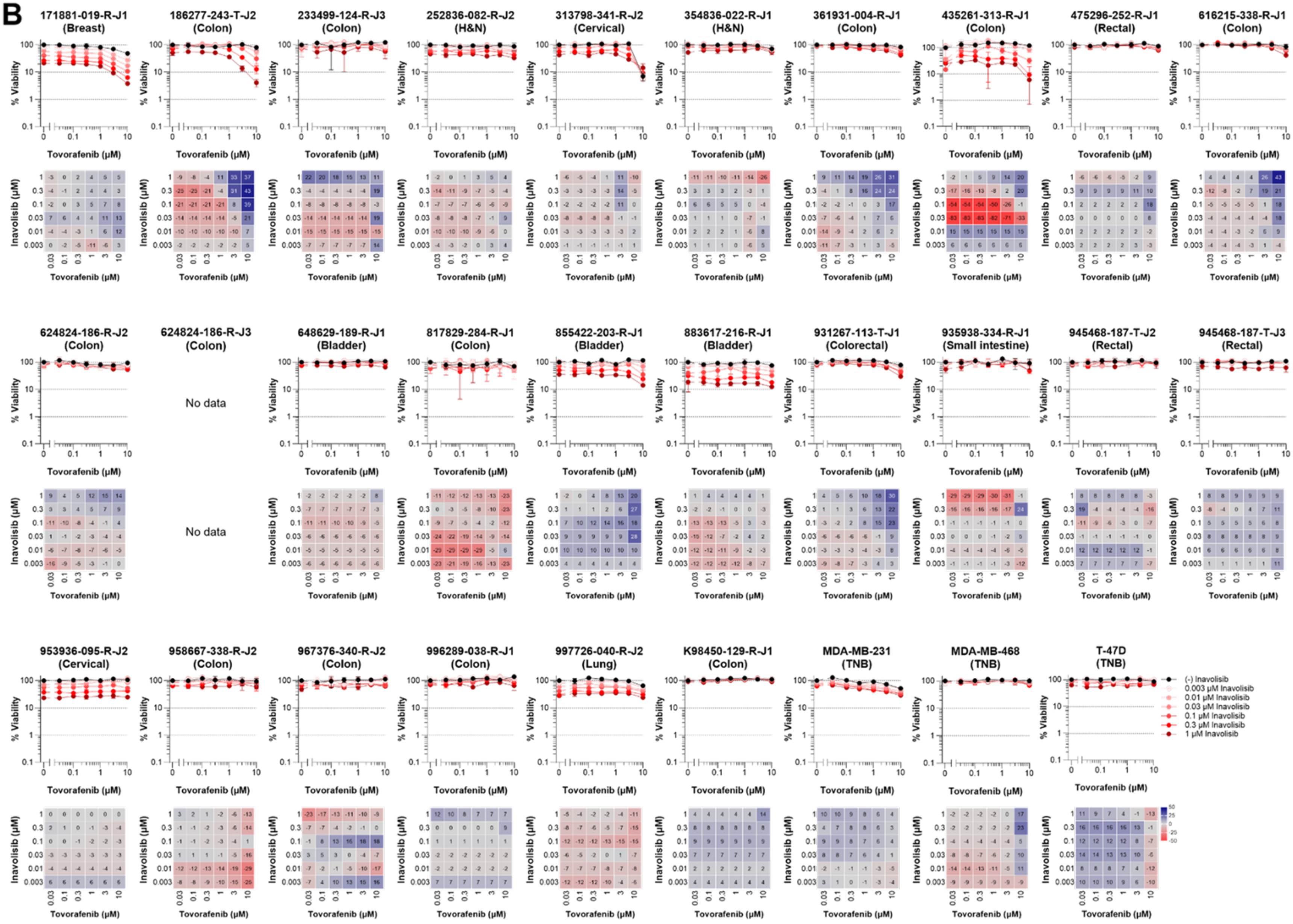
B
171881-019-R-J1 (Breast)
186277-243-T-J2 (Colon)
233499-124-R-J3 (Colon)
252836-082-R-J2 (H&N)
313798-341-R-J2 (Cervical)
354836-022-R-J1 (H&N)
361931-004-R-J1 (Colon)
435261-313-R-J1 (Colon)
475296-252-R-J1 (Rectal)
616215-338-R-J1 (Colon)
624824-186-R-J2 (Colon)
624824-186-R-J3 (Colon)
No data
No data
648629-189-R-J1 (Bladder)
817829-284-R-J1 (Colon)
855422-203-R-J1 (Bladder)
883617-216-R-J1 (Bladder)
931267-113-T-J1 (Colorectal)
935938-334-R-J1 (Small intestine)
945468-187-T-J2 (Rectal)
945468-187-T-J3 (Rectal)
953936-095-R-J2 (Cervical)
958667-338-R-J2 (Colon)
967376-340-R-J2 (Colon)
996289-038-R-J1 (Colon)
997726-040-R-J2 (Lung)
K98450-129-R-J1 (Colon)
MDA-MB-231 (TNB)
MDA-MB-468 (TNB)
T-47D (TNB)
% Viability
Tovorafenib (µM)
Inavolisib (µM)
(-) Inavolisib
0.003 µM Inavolisib
0.01 µM Inavolisib
0.03 µM Inavolisib
0.1 µM Inavolisib
0.3 µM Inavolisib
1 µM Inavolisib

C

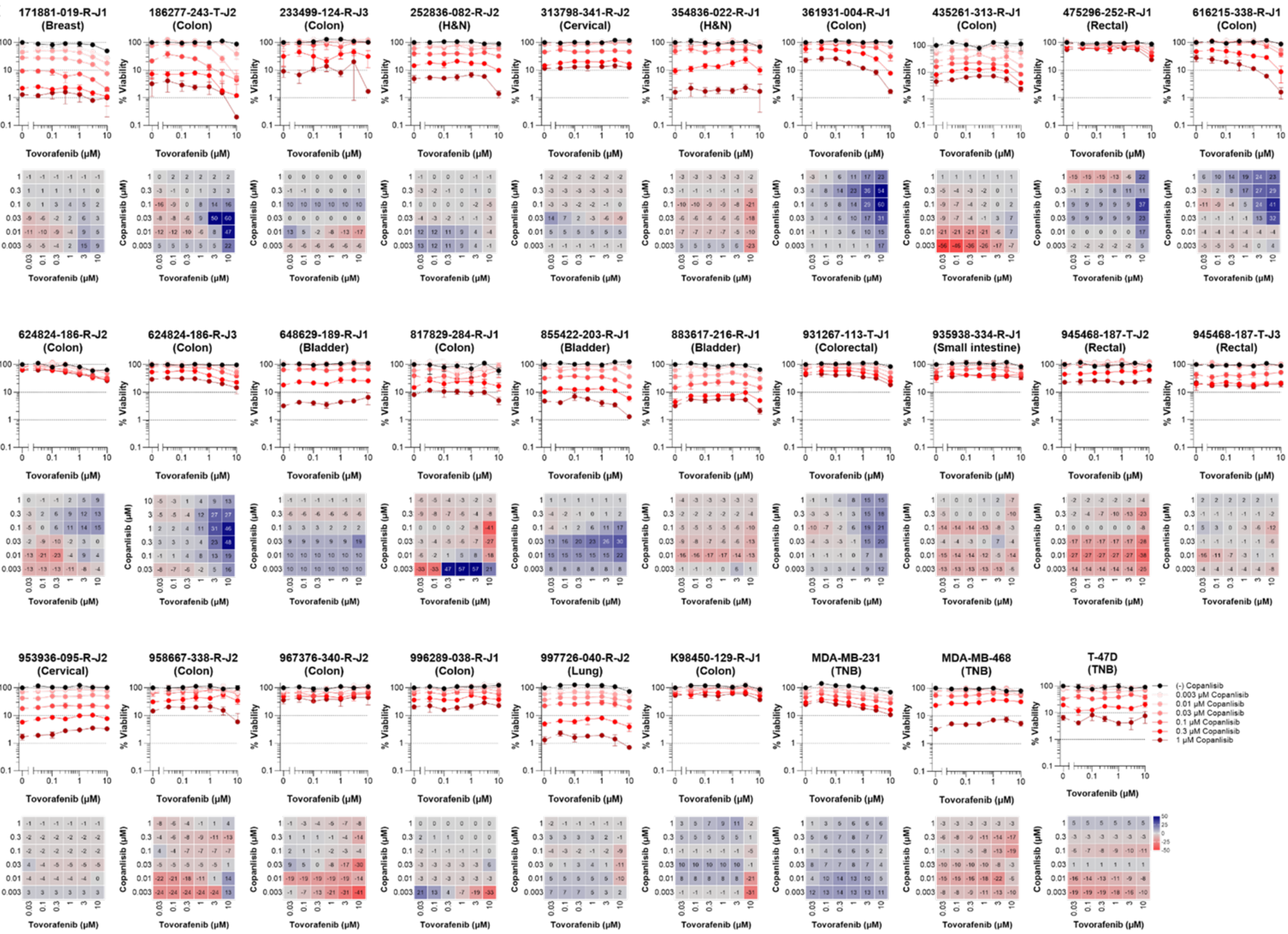
171881-019-R-J1 (Breast)
186277-243-T-J2 (Colon)
233499-124-R-J3 (Colon)
252836-082-R-J2 (H&N)
313798-341-R-J2 (Cervical)
354836-022-R-J1 (H&N)
361931-004-R-J1 (Colon)
435261-313-R-J1 (Colon)
475296-252-R-J1 (Rectal)
616215-338-R-J1 (Colon)
624824-186-R-J2 (Colon)
624824-186-R-J3 (Colon)
648629-189-R-J1 (Bladder)
817829-284-R-J1 (Colon)
855422-203-R-J1 (Bladder)
883617-216-R-J1 (Bladder)
931267-113-T-J1 (Colorectal)
935938-334-R-J1 (Small intestine)
945468-187-T-J2 (Rectal)
945468-187-T-J3 (Rectal)
953936-095-R-J2 (Cervical)
958667-338-R-J2 (Colon)
967376-340-R-J2 (Colon)
996289-038-R-J1 (Colon)
997726-040-R-J2 (Lung)
K98450-129-R-J1 (Colon)
MDA-MB-231 (TNB)
MDA-MB-468 (TNB)
T-47D (TNB)
% Viability
Tovorafenib (µM)
Copanlisib (µM)
(-) Copanlisib
0.003 µM Copanlisib
0.01 µM Copanlisib
0.03 µM Copanlisib
0.1 µM Copanlisib
0.3 µM Copanlisib
1 µM Copanlisib

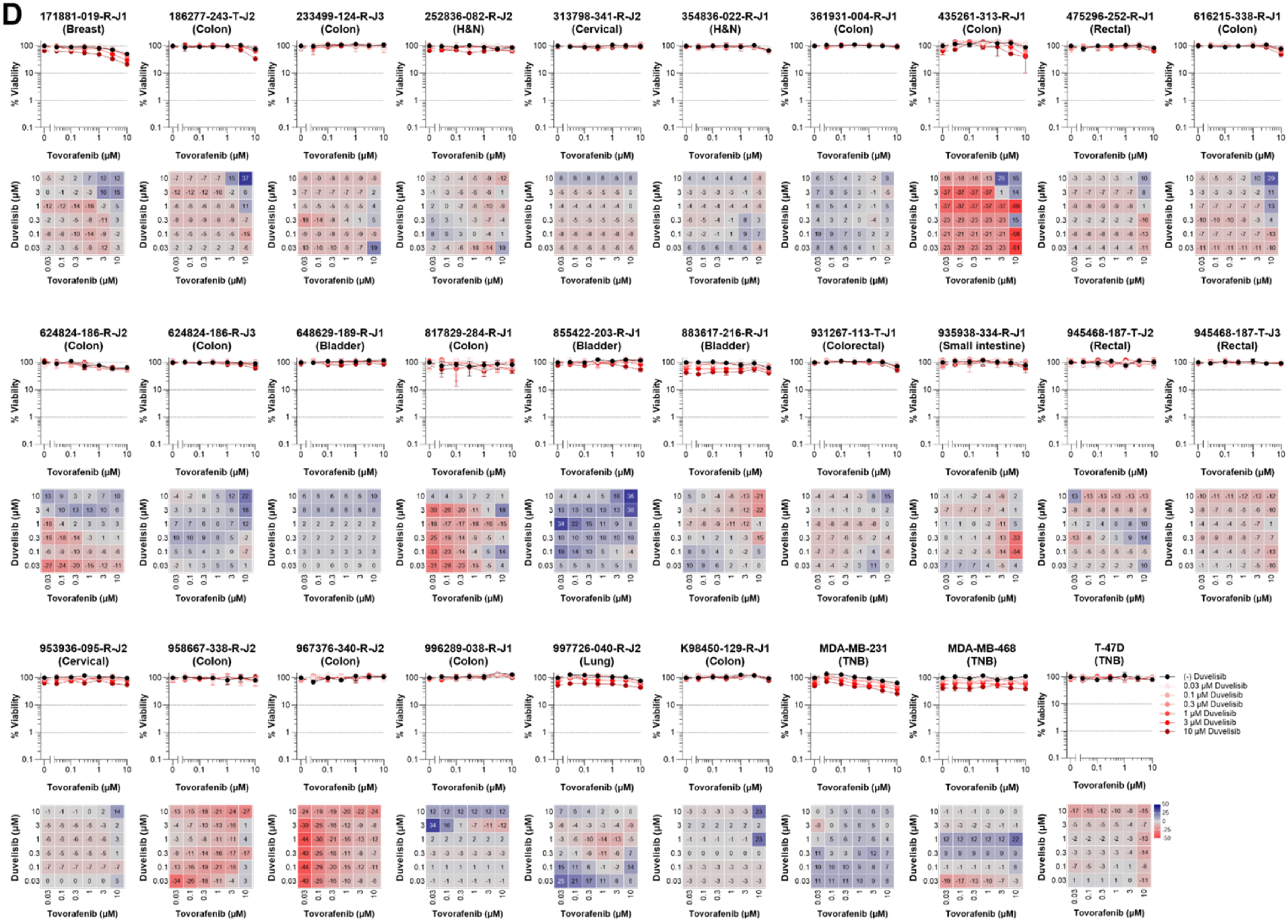
D
171881-019-R-J1 (Breast)
186277-243-T-J2 (Colon)
233499-124-R-J3 (Colon)
252836-082-R-J2 (H&N)
313798-341-R-J2 (Cervical)
354836-022-R-J1 (H&N)
361931-004-R-J1 (Colon)
435261-313-R-J1 (Colon)
475296-252-R-J1 (Rectal)
616215-338-R-J1 (Colon)
624824-186-R-J2 (Colon)
624824-186-R-J3 (Colon)
648629-189-R-J1 (Bladder)
817829-284-R-J1 (Colon)
855422-203-R-J1 (Bladder)
883617-216-R-J1 (Bladder)
931267-113-T-J1 (Colorectal)
935938-334-R-J1 (Small intestine)
945468-187-T-J2 (Rectal)
945468-187-T-J3 (Rectal)
953936-095-R-J2 (Cervical)
958667-338-R-J2 (Colon)
967376-340-R-J2 (Colon)
996289-038-R-J1 (Colon)
997726-040-R-J2 (Lung)
K98450-129-R-J1 (Colon)
MDA-MB-231 (TNB)
MDA-MB-468 (TNB)
T-47D (TNB)
% Viability
Tovorafenib (μM)
Duvelisib (μM)
(-) Duvelisib
0.03 μM Duvelisib
0.1 μM Duvelisib
0.3 μM Duvelisib
1 μM Duvelisib
3 μM Duvelisib
10 μM Duvelisib

Supplemental Figure S5. Combination activity for tovorafenib (DAY101) with a PI3K inhibitor. Concentration-response graphs (*top*, mean ± SD, *n* = 3 technical replicates) and Bliss independence scores across the concentration matrix (*bottom*, mean of *n* = 3 technical replicates) are shown as numerical values and heat maps (blue indicates synergy, gray indicates additivity, and red indicates antagonism). Data are presented, if available, from twenty-nine malignant cell lines grown as multi-cell type tumor spheroids and treated with tovorafenib (DAY101) in combination with (A) alpelisib, (B) inavolisib, (C) copanlisib, or (D) duvelisib. The tumor model name and type are indicated above each set of graphs.

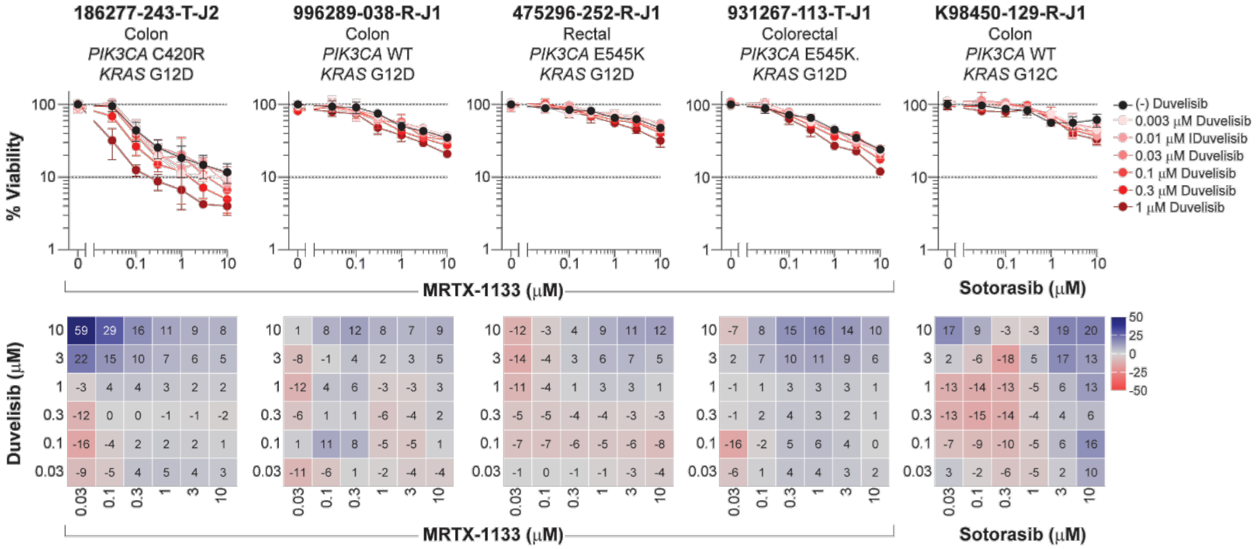

Supplemental Figure S6. Duvelisib in combination with the allele-specific KRAS inhibitor MRTX1133 (G12D) or sotorasib (G12C) in tumor cells carrying the respective variant. Concentration-response graphs (*top*, mean ± SD, n = 3 technical replicates) from combinations of MRTX1133 or sotorasib with duvelisib are shown with corresponding Bliss independence scores from each combination matrix (*bottom*, mean of n = 3 technical replicates) displayed numerically and as a heat map (blue indicates synergy, gray indicates additivity, and red indicates antagonism). Tumor cell line characteristics, including model name, cancer type, and PIK3CA and KRAS mutation status, are annotated.

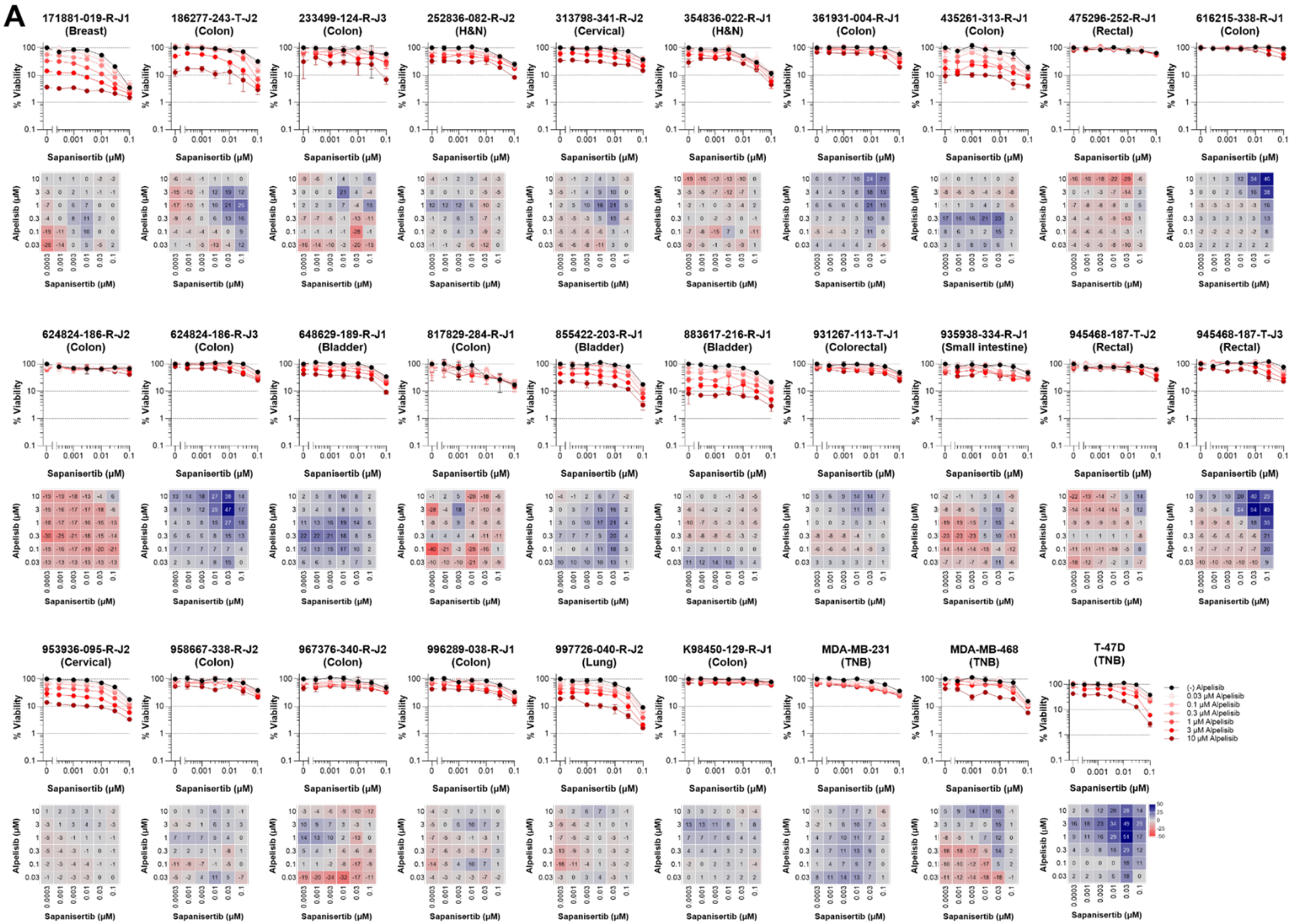
A
171881-019-R-J1 (Breast)
186277-243-T-J2 (Colon)
233499-124-R-J3 (Colon)
252836-082-R-J2 (H&N)
313798-341-R-J2 (Cervical)
354836-022-R-J1 (H&N)
361931-004-R-J1 (Colon)
435261-313-R-J1 (Colon)
475296-252-R-J1 (Rectal)
616215-338-R-J1 (Colon)
624824-186-R-J2 (Colon)
624824-186-R-J3 (Colon)
648629-189-R-J1 (Bladder)
817829-284-R-J1 (Colon)
855422-203-R-J1 (Bladder)
883617-216-R-J1 (Bladder)
931267-113-T-J1 (Colorectal)
935938-334-R-J1 (Small intestine)
945468-187-T-J2 (Rectal)
945468-187-T-J3 (Rectal)
953936-095-R-J2 (Cervical)
958667-338-R-J2 (Colon)
967376-340-R-J2 (Colon)
996289-038-R-J1 (Colon)
997726-040-R-J2 (Lung)
K98450-129-R-J1 (Colon)
MDA-MB-231 (TNB)
MDA-MB-468 (TNB)
T-47D (TNB)
% Viability
Sapanisertib (µM)
Alpelisib (µM)
(-) Alpelisib
0.03 µM Alpelisib
0.1 µM Alpelisib
0.3 µM Alpelisib
1 µM Alpelisib
3 µM Alpelisib
10 µM Alpelisib

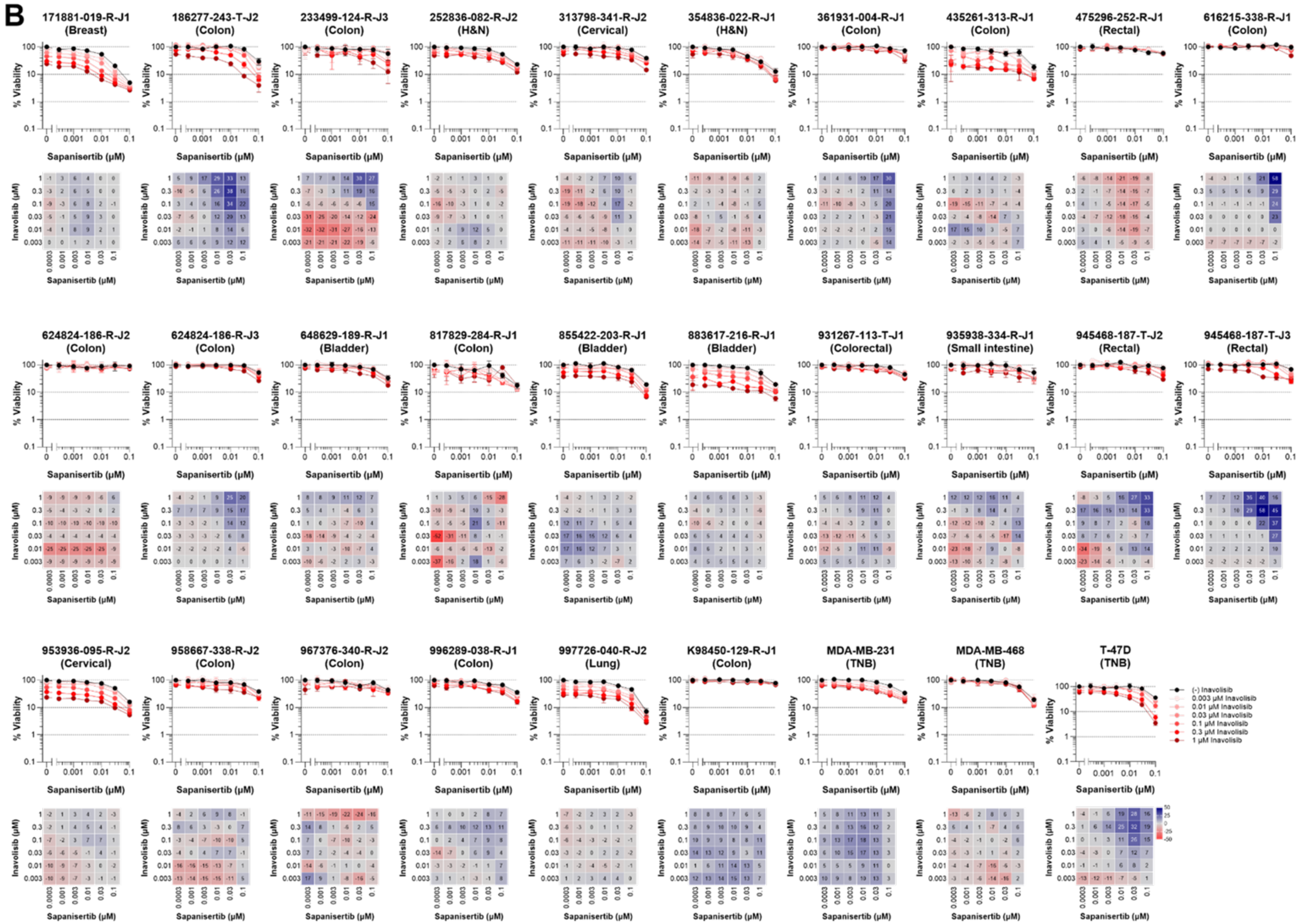
B
171881-019-R-J1 (Breast)
186277-243-T-J2 (Colon)
233499-124-R-J3 (Colon)
252836-082-R-J2 (H&N)
313798-341-R-J2 (Cervical)
354836-022-R-J1 (H&N)
361931-004-R-J1 (Colon)
435261-313-R-J1 (Colon)
475296-252-R-J1 (Rectal)
616215-338-R-J1 (Colon)
624824-186-R-J2 (Colon)
624824-186-R-J3 (Colon)
648629-189-R-J1 (Bladder)
817829-284-R-J1 (Colon)
855422-203-R-J1 (Bladder)
883617-216-R-J1 (Bladder)
931267-113-T-J1 (Colorectal)
935938-334-R-J1 (Small intestine)
945468-187-T-J2 (Rectal)
945468-187-T-J3 (Rectal)
953936-095-R-J2 (Cervical)
958667-338-R-J2 (Colon)
967376-340-R-J2 (Colon)
996289-038-R-J1 (Colon)
997726-040-R-J2 (Lung)
K98450-129-R-J1 (Colon)
MDA-MB-231 (TNB)
MDA-MB-468 (TNB)
T-47D (TNB)
% Viability
Sapanisertib (µM)
Inavolisib (µM)
(-) Inavolisib
0.003 µM Inavolisib
0.01 µM Inavolisib
0.03 µM Inavolisib
0.1 µM Inavolisib
0.3 µM Inavolisib
1 µM Inavolisib

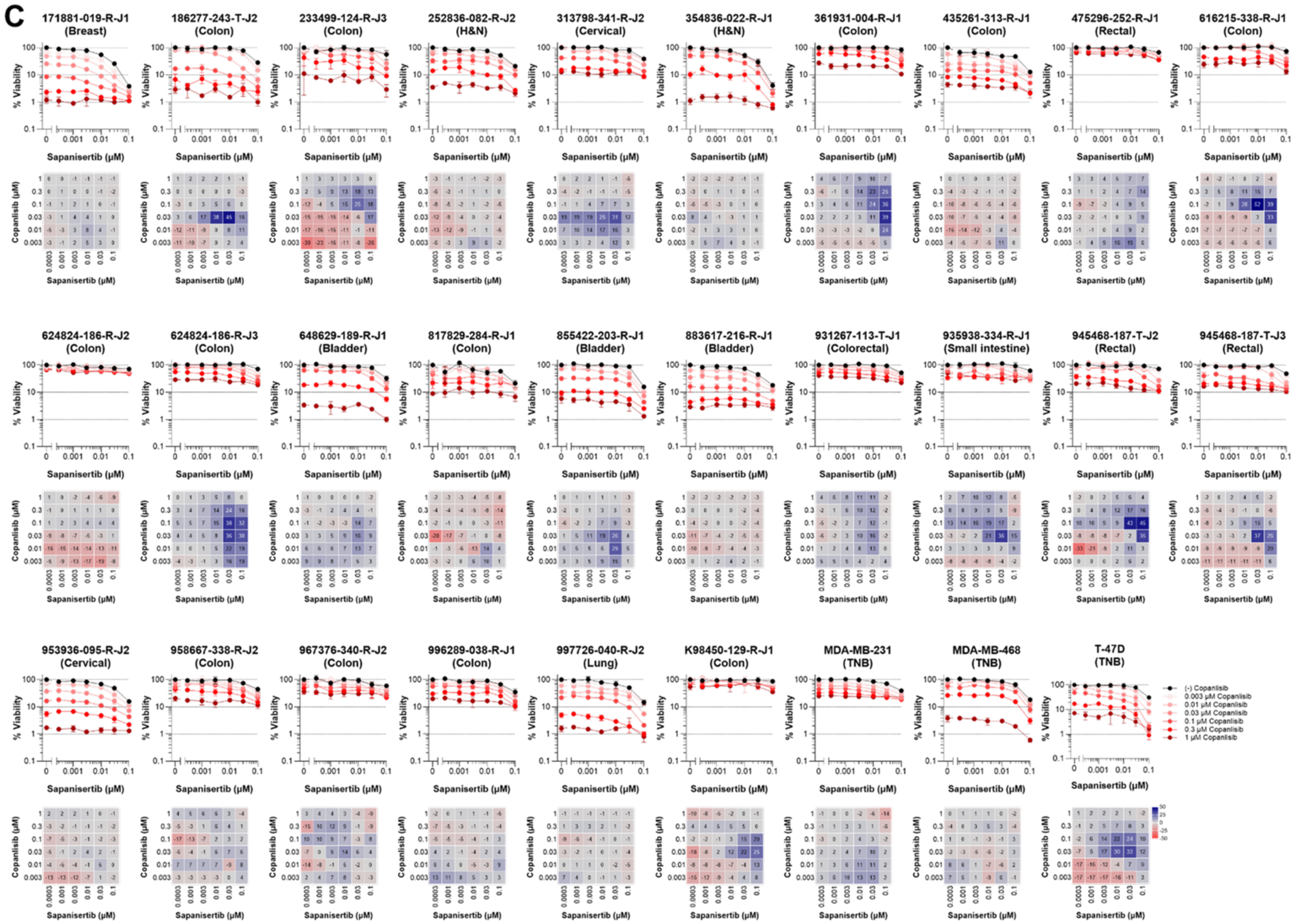
C
171881-019-R-J1 (Breast)
186277-243-T-J2 (Colon)
233499-124-R-J3 (Colon)
252836-082-R-J2 (H&N)
313798-341-R-J2 (Cervical)
354836-022-R-J1 (H&N)
361931-004-R-J1 (Colon)
435261-313-R-J1 (Colon)
475296-252-R-J1 (Rectal)
616215-338-R-J1 (Colon)
624824-186-R-J2 (Colon)
624824-186-R-J3 (Colon)
648629-189-R-J1 (Bladder)
817829-284-R-J1 (Colon)
855422-203-R-J1 (Bladder)
883617-216-R-J1 (Bladder)
931267-113-T-J1 (Colorectal)
935938-334-R-J1 (Small intestine)
945468-187-T-J2 (Rectal)
945468-187-T-J3 (Rectal)
953936-095-R-J2 (Cervical)
958667-338-R-J2 (Colon)
967376-340-R-J2 (Colon)
996289-038-R-J1 (Colon)
997726-040-R-J2 (Lung)
K98450-129-R-J1 (Colon)
MDA-MB-231 (TNB)
MDA-MB-468 (TNB)
T-47D (TNB)
% Viability
Sapanisertib (μM)
Copanlisib (μM)
(-) Copanlisib
0.003 μM Copanlisib
0.01 μM Copanlisib
0.03 μM Copanlisib
0.1 μM Copanlisib
0.3 μM Copanlisib
1 μM Copanlisib

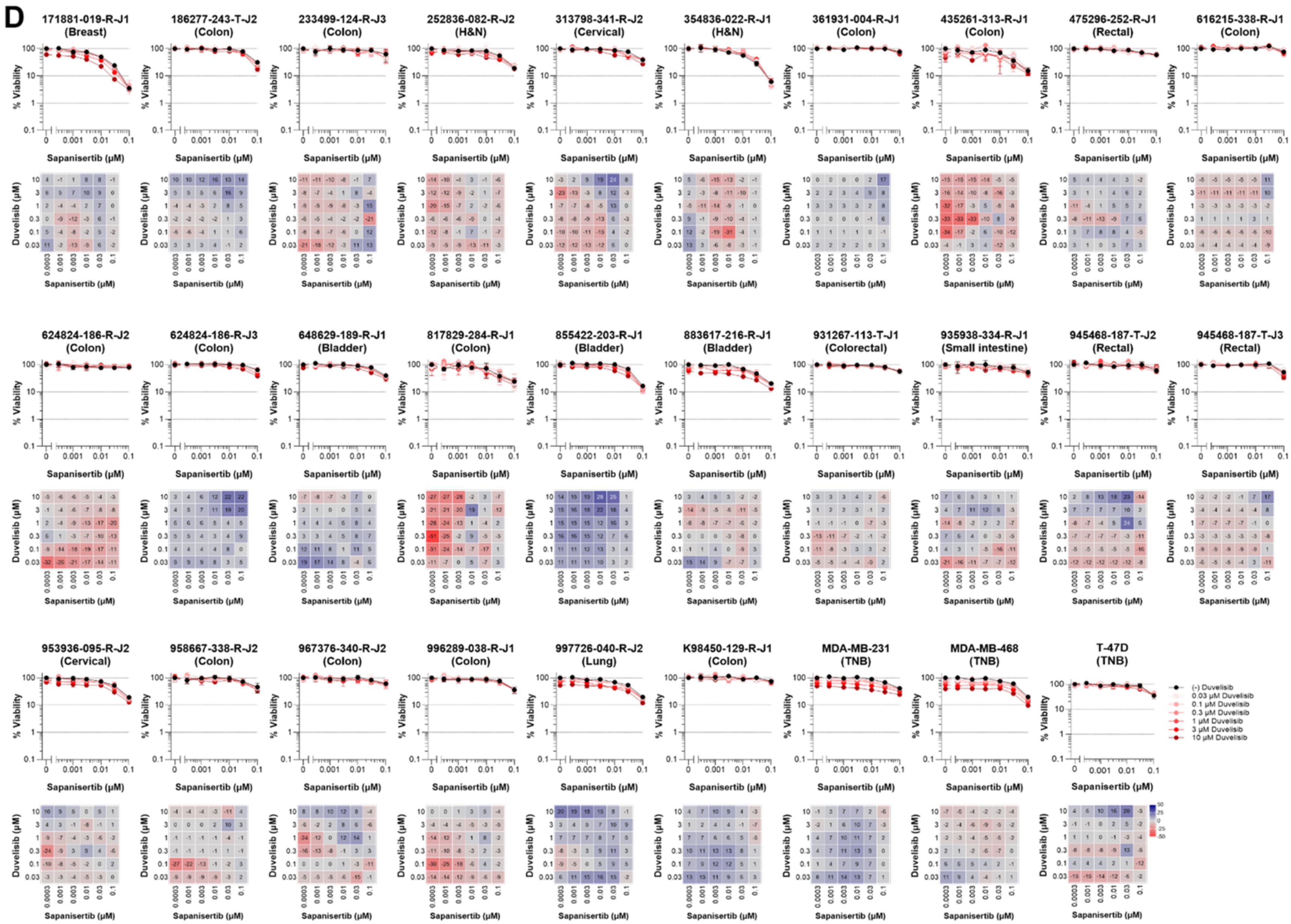
D
171881-019-R-J1 (Breast)
186277-243-T-J2 (Colon)
233499-124-R-J3 (Colon)
252836-082-R-J2 (H&N)
313798-341-R-J2 (Cervical)
354836-022-R-J1 (H&N)
361931-004-R-J1 (Colon)
435261-313-R-J1 (Colon)
475296-252-R-J1 (Rectal)
616215-338-R-J1 (Colon)
624824-186-R-J2 (Colon)
624824-186-R-J3 (Colon)
648629-189-R-J1 (Bladder)
817829-284-R-J1 (Colon)
855422-203-R-J1 (Bladder)
883617-216-R-J1 (Bladder)
931267-113-T-J1 (Colorectal)
935938-334-R-J1 (Small intestine)
945468-187-T-J2 (Rectal)
945468-187-T-J3 (Rectal)
953936-095-R-J2 (Cervical)
958667-338-R-J2 (Colon)
967376-340-R-J2 (Colon)
996289-038-R-J1 (Colon)
997726-040-R-J2 (Lung)
K98450-129-R-J1 (Colon)
MDA-MB-231 (TNB)
MDA-MB-468 (TNB)
T-47D (TNB)
% Viability
Sapanisertib (μM)
Duvelisib (μM)
(-) Duvelisib
0.03 μM Duvelisib
0.1 μM Duvelisib
0.3 μM Duvelisib
1 μM Duvelisib
3 μM Duvelisib
10 μM Duvelisib

Supplemental Figure S7. Combination activity for sapanisertib with a PI3K inhibitor. Concentration-response graphs (*top*, mean ± SD, n = 3 technical replicates) and Bliss independence scores across the concentration matrix (*bottom*, mean of n = 3 technical replicates) are shown as numerical values and heat maps (blue indicates synergy, gray indicates additivity, and red indicates antagonism). Data are presented, if available, from twenty-nine malignant cell lines grown as multi-cell type tumor spheroids and treated with sapanisertib in combination with (A) alpelisib, (B) inavolisib, (C) copanlisib, or (D) duvelisib. The tumor model name and type are indicated above each set of graphs.

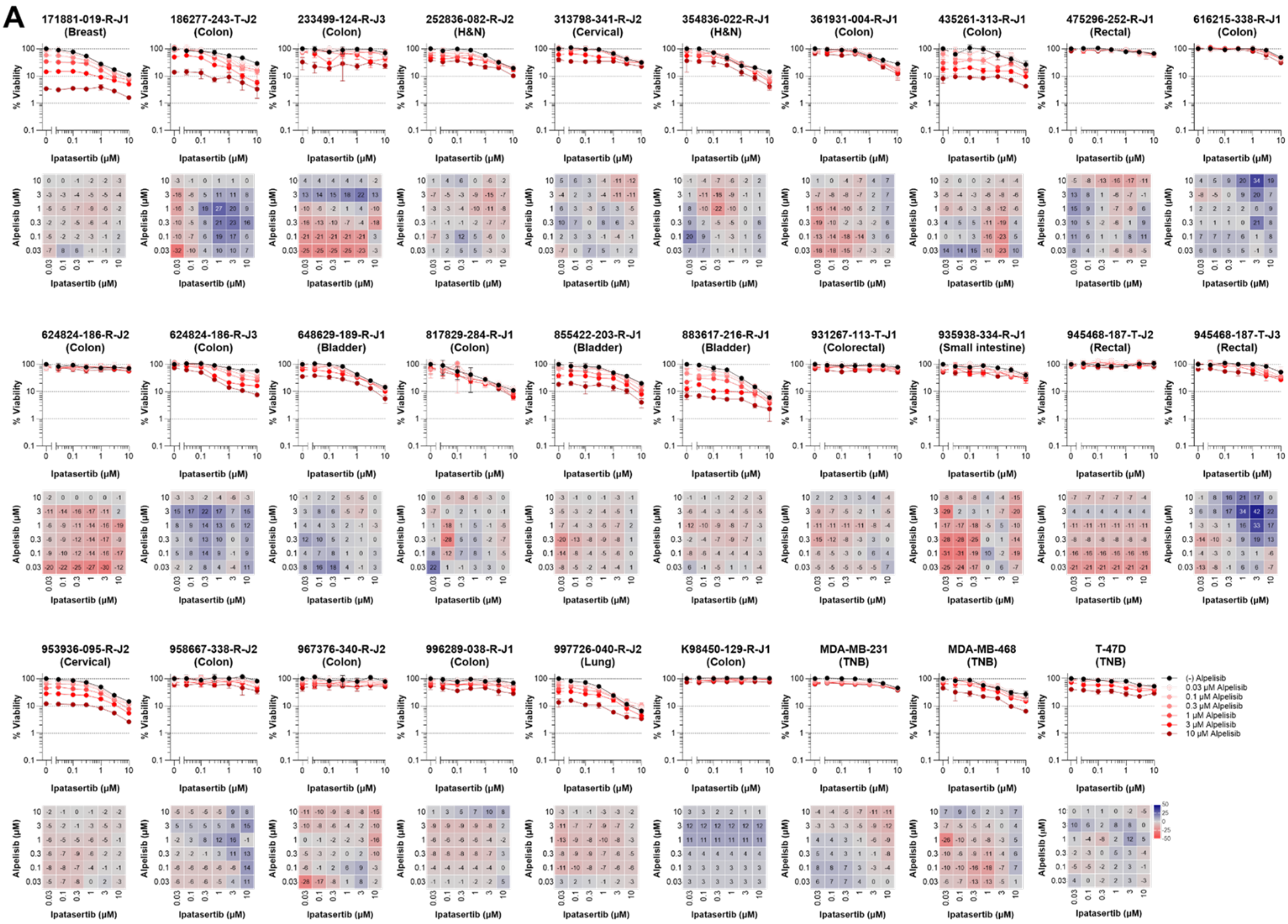
A
171881-019-R-J1 (Breast)
186277-243-T-J2 (Colon)
233499-124-R-J3 (Colon)
252836-082-R-J2 (H&N)
313798-341-R-J2 (Cervical)
354836-022-R-J1 (H&N)
361931-004-R-J1 (Colon)
435261-313-R-J1 (Colon)
475296-252-R-J1 (Rectal)
616215-338-R-J1 (Colon)
624824-186-R-J2 (Colon)
624824-186-R-J3 (Colon)
648629-189-R-J1 (Bladder)
817829-284-R-J1 (Colon)
855422-203-R-J1 (Bladder)
883617-216-R-J1 (Bladder)
931267-113-T-J1 (Colorectal)
935938-334-R-J1 (Small intestine)
945468-187-T-J2 (Rectal)
945468-187-T-J3 (Rectal)
953936-095-R-J2 (Cervical)
958667-338-R-J2 (Colon)
967376-340-R-J2 (Colon)
996289-038-R-J1 (Colon)
997726-040-R-J2 (Lung)
K98450-129-R-J1 (Colon)
MDA-MB-231 (TNB)
MDA-MB-468 (TNB)
T-47D (TNB)
% Viability
Ipatasertib (µM)
Alpelisib (µM)
(-) Alpelisib
0.03 µM Alpelisib
0.1 µM Alpelisib
0.3 µM Alpelisib
1 µM Alpelisib
3 µM Alpelisib
10 µM Alpelisib

B

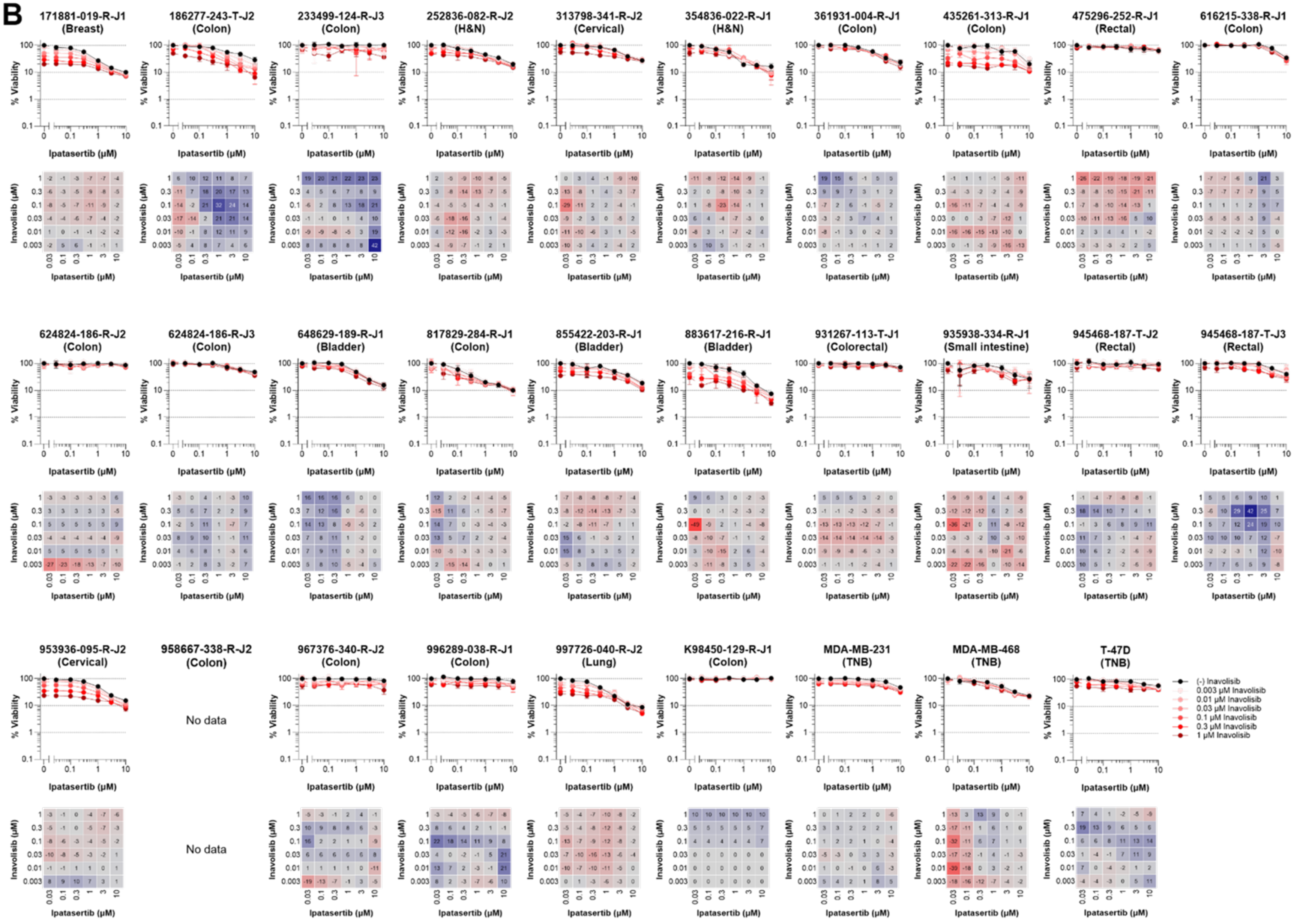
171881-019-R-J1 (Breast)
186277-243-T-J2 (Colon)
233499-124-R-J3 (Colon)
252836-082-R-J2 (H&N)
313798-341-R-J2 (Cervical)
354836-022-R-J1 (H&N)
361931-004-R-J1 (Colon)
435261-313-R-J1 (Colon)
475296-252-R-J1 (Rectal)
616215-338-R-J1 (Colon)
624824-186-R-J2 (Colon)
624824-186-R-J3 (Colon)
648629-189-R-J1 (Bladder)
817829-284-R-J1 (Colon)
855422-203-R-J1 (Bladder)
883617-216-R-J1 (Bladder)
931267-113-T-J1 (Colorectal)
935938-334-R-J1 (Small intestine)
945468-187-T-J2 (Rectal)
945468-187-T-J3 (Rectal)
953936-095-R-J2 (Cervical)
958667-338-R-J2 (Colon)
No data
No data
967376-340-R-J2 (Colon)
996289-038-R-J1 (Colon)
997726-040-R-J2 (Lung)
K98450-129-R-J1 (Colon)
MDA-MB-231 (TNB)
MDA-MB-468 (TNB)
T-47D (TNB)
% Viability
Ipatasertib (µM)
Inavolisib (µM)
(-) Inavolisib
0.003 µM Inavolisib
0.01 µM Inavolisib
0.03 µM Inavolisib
0.1 µM Inavolisib
0.3 µM Inavolisib
1 µM Inavolisib

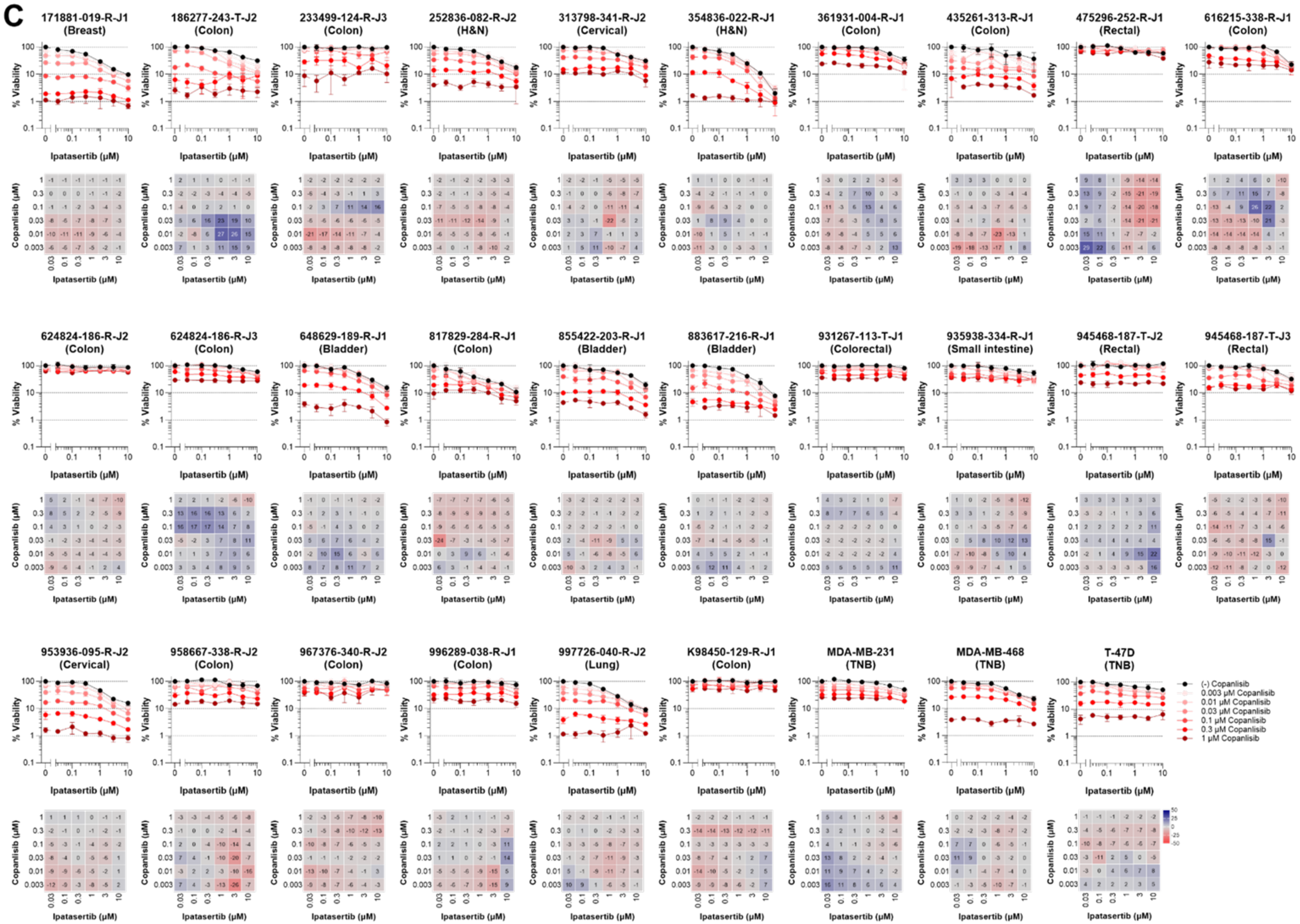
C
171881-019-R-J1 (Breast)
186277-243-T-J2 (Colon)
233499-124-R-J3 (Colon)
252836-082-R-J2 (H&N)
313798-341-R-J2 (Cervical)
354836-022-R-J1 (H&N)
361931-004-R-J1 (Colon)
435261-313-R-J1 (Colon)
475296-252-R-J1 (Rectal)
616215-338-R-J1 (Colon)
624824-186-R-J2 (Colon)
624824-186-R-J3 (Colon)
648629-189-R-J1 (Bladder)
817829-284-R-J1 (Colon)
855422-203-R-J1 (Bladder)
883617-216-R-J1 (Bladder)
931267-113-T-J1 (Colorectal)
935938-334-R-J1 (Small intestine)
945468-187-T-J2 (Rectal)
945468-187-T-J3 (Rectal)
953936-095-R-J2 (Cervical)
958667-338-R-J2 (Colon)
967376-340-R-J2 (Colon)
996289-038-R-J1 (Colon)
997726-040-R-J2 (Lung)
K98450-129-R-J1 (Colon)
MDA-MB-231 (TNB)
MDA-MB-468 (TNB)
T-47D (TNB)
% Viability
Ipatasertib (µM)
Copanlisib (µM)
(-) Copanlisib
0.003 µM Copanlisib
0.01 µM Copanlisib
0.03 µM Copanlisib
0.1 µM Copanlisib
0.3 µM Copanlisib
1 µM Copanlisib

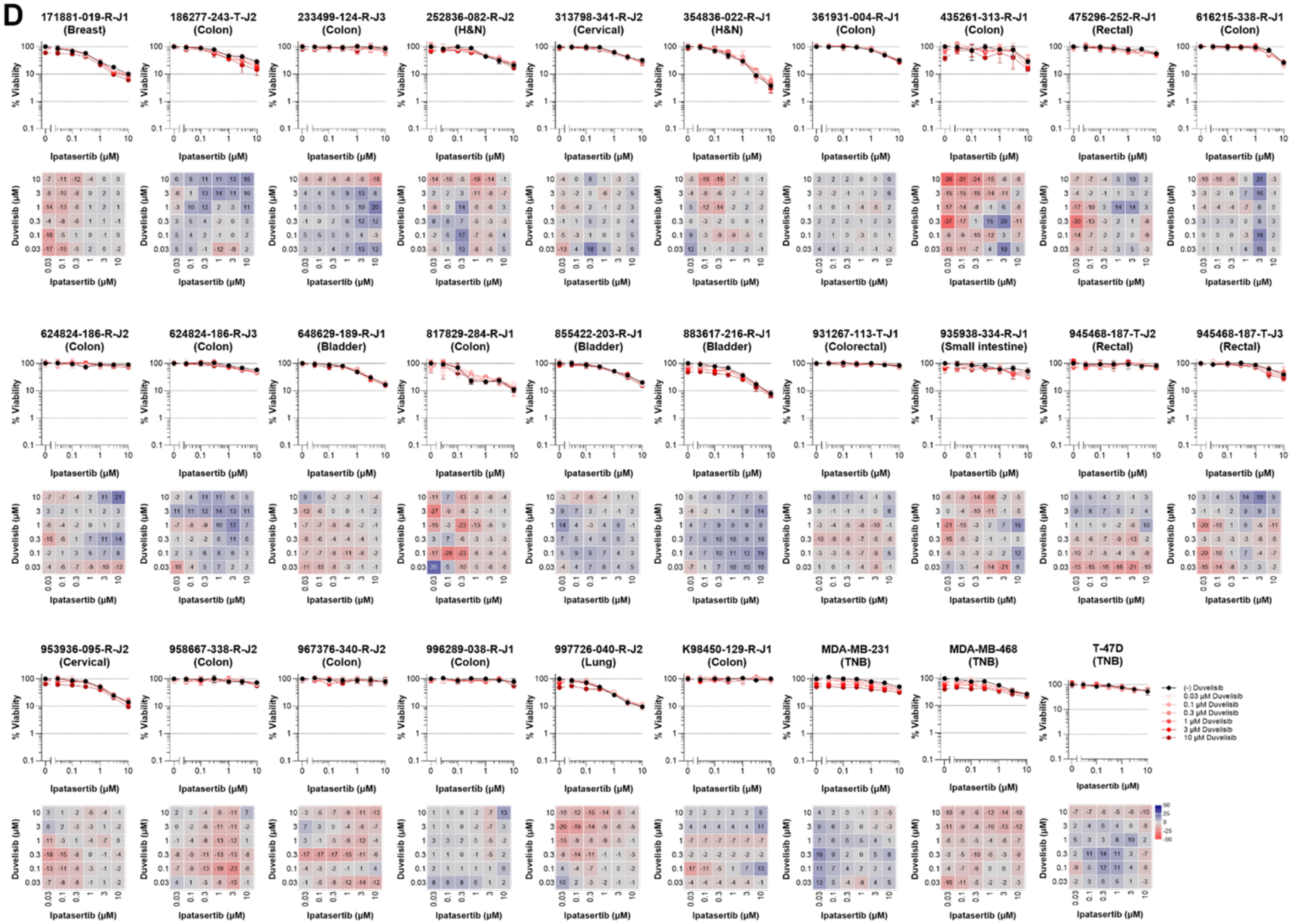
D
171881-019-R-J1 (Breast)
186277-243-T-J2 (Colon)
233499-124-R-J3 (Colon)
252836-082-R-J2 (H&N)
313798-341-R-J2 (Cervical)
354836-022-R-J1 (H&N)
361931-004-R-J1 (Colon)
435261-313-R-J1 (Colon)
475296-252-R-J1 (Rectal)
616215-338-R-J1 (Colon)
624824-186-R-J2 (Colon)
624824-186-R-J3 (Colon)
648629-189-R-J1 (Bladder)
817829-284-R-J1 (Colon)
855422-203-R-J1 (Bladder)
883617-216-R-J1 (Bladder)
931267-113-T-J1 (Colorectal)
935938-334-R-J1 (Small intestine)
945468-187-T-J2 (Rectal)
945468-187-T-J3 (Rectal)
953936-095-R-J2 (Cervical)
958667-338-R-J2 (Colon)
967376-340-R-J2 (Colon)
996289-038-R-J1 (Colon)
997726-040-R-J2 (Lung)
K98450-129-R-J1 (Colon)
MDA-MB-231 (TNB)
MDA-MB-468 (TNB)
T-47D (TNB)
% Viability
Ipatasertib (µM)
Duvelisib (µM)
(-) Duvelisib
0.03 µM Duvelisib
0.1 µM Duvelisib
0.3 µM Duvelisib
1 µM Duvelisib
3 µM Duvelisib
10 µM Duvelisib

Supplemental Figure S8. Combination activity from ipatasertib with a PI3K inhibitor. Concentration-response graphs (*top*, mean ± SD, $n = 3$ technical replicates) and Bliss independence scores across the concentration matrix (*bottom*, mean of $n = 3$ technical replicates) are shown as numerical values and heat maps (blue indicates synergy, gray indicates additivity, and red indicates antagonism). Data are presented, if available, from twenty-nine malignant cell lines grown as multi-cell type tumor spheroids and treated with ipatasertib in combination with (A) alpelisib, (B) inavolisib, (C) copanlisib, or (D) duvelisib. The tumor model name and type are indicated above each set of graphs.

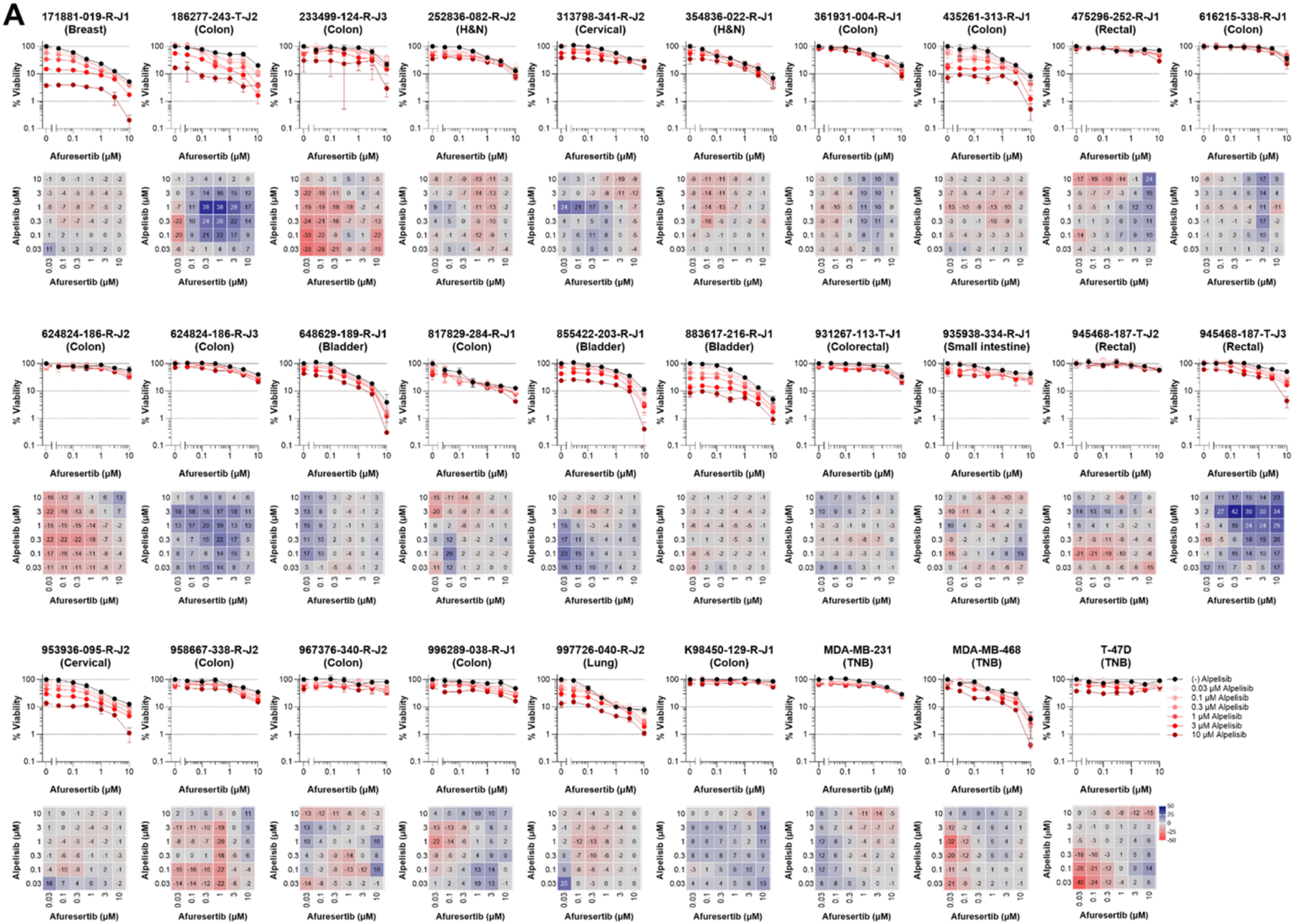
A
171881-019-R-J1 (Breast)
186277-243-T-J2 (Colon)
233499-124-R-J3 (Colon)
252836-082-R-J2 (H&N)
313798-341-R-J2 (Cervical)
354836-022-R-J1 (H&N)
361931-004-R-J1 (Colon)
435261-313-R-J1 (Colon)
475296-252-R-J1 (Rectal)
616215-338-R-J1 (Colon)
624824-186-R-J2 (Colon)
624824-186-R-J3 (Colon)
648629-189-R-J1 (Bladder)
817829-284-R-J1 (Colon)
855422-203-R-J1 (Bladder)
883617-216-R-J1 (Bladder)
931267-113-T-J1 (Colorectal)
935938-334-R-J1 (Small intestine)
945468-187-T-J2 (Rectal)
945468-187-T-J3 (Rectal)
953936-095-R-J2 (Cervical)
958667-338-R-J2 (Colon)
967376-340-R-J2 (Colon)
996289-038-R-J1 (Colon)
997726-040-R-J2 (Lung)
K98450-129-R-J1 (Colon)
MDA-MB-231 (TNB)
MDA-MB-468 (TNB)
T-47D (TNB)
% Viability
Afuresertib (µM)
Alpelisib (µM)
(-) Alpelisib
0.03 µM Alpelisib
0.1 µM Alpelisib
0.3 µM Alpelisib
1 µM Alpelisib
3 µM Alpelisib
10 µM Alpelisib
50
25
0
-25
-50

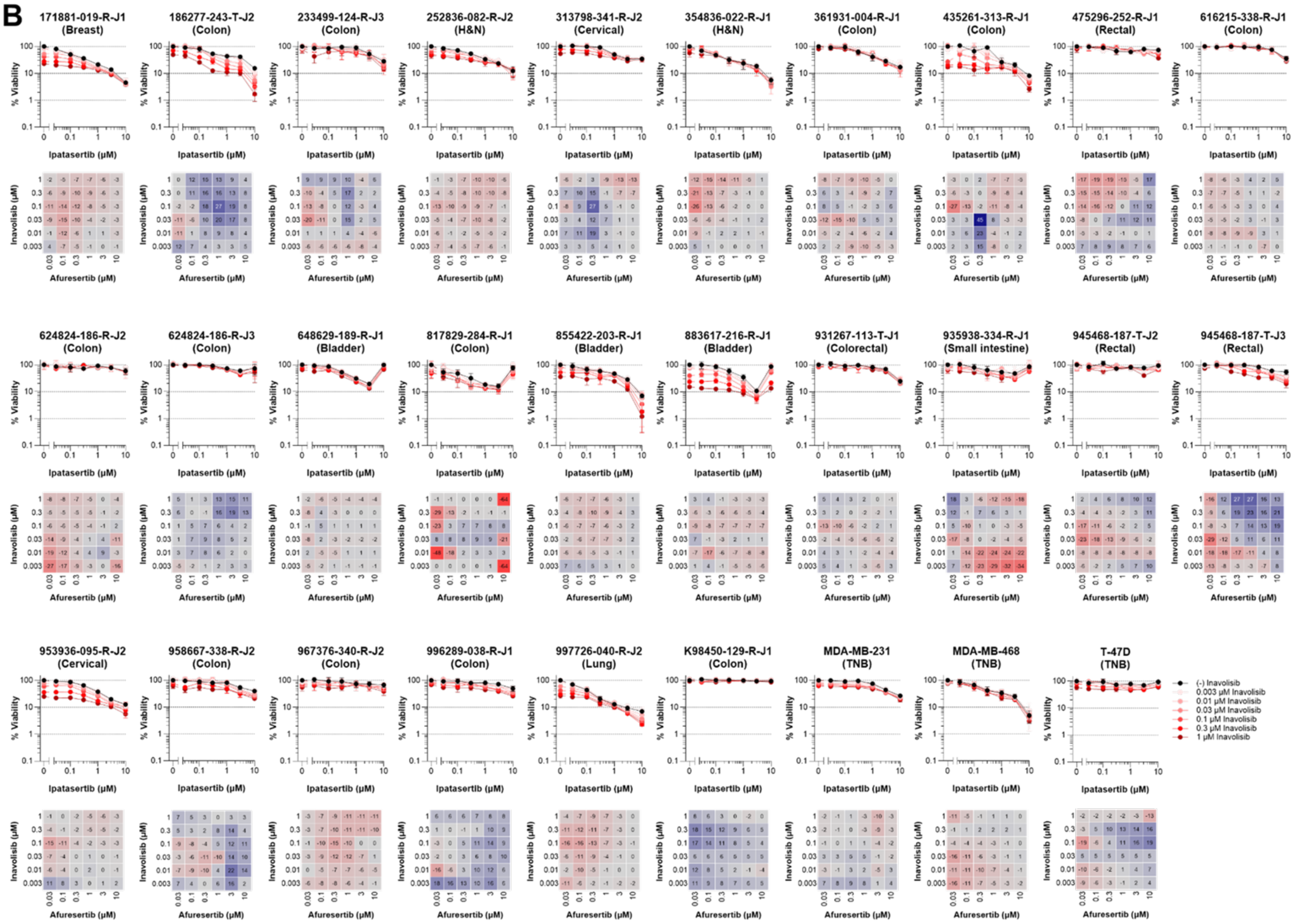
B
171881-019-R-J1 (Breast)
186277-243-T-J2 (Colon)
233499-124-R-J3 (Colon)
252836-082-R-J2 (H&N)
313798-341-R-J2 (Cervical)
354836-022-R-J1 (H&N)
361931-004-R-J1 (Colon)
435261-313-R-J1 (Colon)
475296-252-R-J1 (Rectal)
616215-338-R-J1 (Colon)
624824-186-R-J2 (Colon)
624824-186-R-J3 (Colon)
648629-189-R-J1 (Bladder)
817829-284-R-J1 (Colon)
855422-203-R-J1 (Bladder)
883617-216-R-J1 (Bladder)
931267-113-T-J1 (Colorectal)
935938-334-R-J1 (Small intestine)
945468-187-T-J2 (Rectal)
945468-187-T-J3 (Rectal)
953936-095-R-J2 (Cervical)
958667-338-R-J2 (Colon)
967376-340-R-J2 (Colon)
996289-038-R-J1 (Colon)
997726-040-R-J2 (Lung)
K98450-129-R-J1 (Colon)
MDA-MB-231 (TNB)
MDA-MB-468 (TNB)
T-47D (TNB)
% Viability
Ipatasertib (μM)
Inavolisib (μM)
Afuresertib (μM)
(-) Inavolisib
0.003 μM Inavolisib
0.01 μM Inavolisib
0.03 μM Inavolisib
0.1 μM Inavolisib
0.3 μM Inavolisib
1 μM Inavolisib

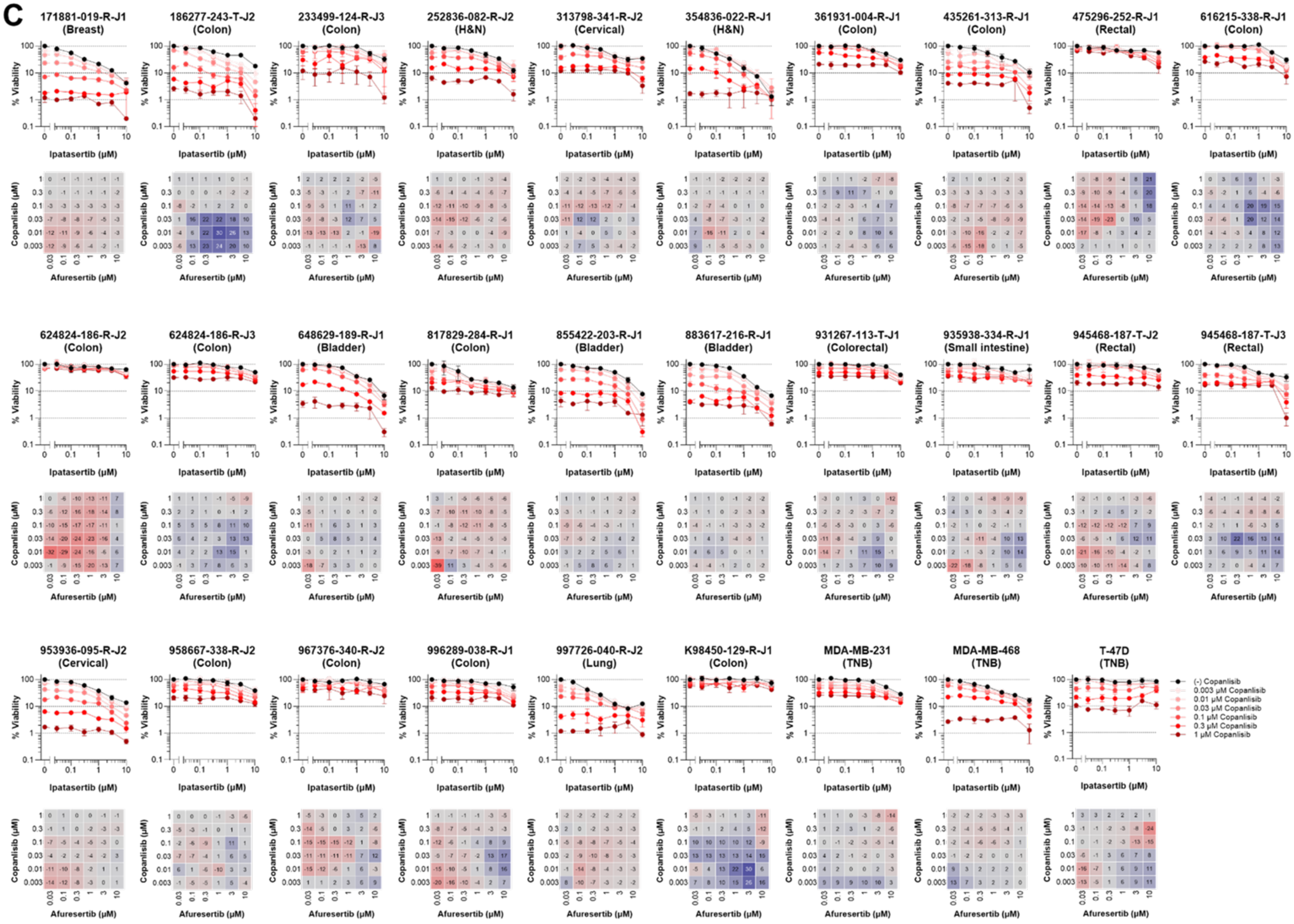
C
171881-019-R-J1 (Breast)
186277-243-T-J2 (Colon)
233499-124-R-J3 (Colon)
252836-082-R-J2 (H&N)
313798-341-R-J2 (Cervical)
354836-022-R-J1 (H&N)
361931-004-R-J1 (Colon)
435261-313-R-J1 (Colon)
475296-252-R-J1 (Rectal)
616215-338-R-J1 (Colon)
624824-186-R-J2 (Colon)
624824-186-R-J3 (Colon)
648629-189-R-J1 (Bladder)
817829-284-R-J1 (Colon)
855422-203-R-J1 (Bladder)
883617-216-R-J1 (Bladder)
931267-113-T-J1 (Colorectal)
935938-334-R-J1 (Small intestine)
945468-187-T-J2 (Rectal)
945468-187-T-J3 (Rectal)
953936-095-R-J2 (Cervical)
958667-338-R-J2 (Colon)
967376-340-R-J2 (Colon)
996289-038-R-J1 (Colon)
997726-040-R-J2 (Lung)
K98450-129-R-J1 (Colon)
MDA-MB-231 (TNB)
MDA-MB-468 (TNB)
T-47D (TNB)
% Viability
Ipatasertib (µM)
Copanlisib (µM)
Afuresertib (µM)
(-) Copanlisib
0.003 µM Copanlisib
0.01 µM Copanlisib
0.03 µM Copanlisib
0.1 µM Copanlisib
0.3 µM Copanlisib
1 µM Copanlisib

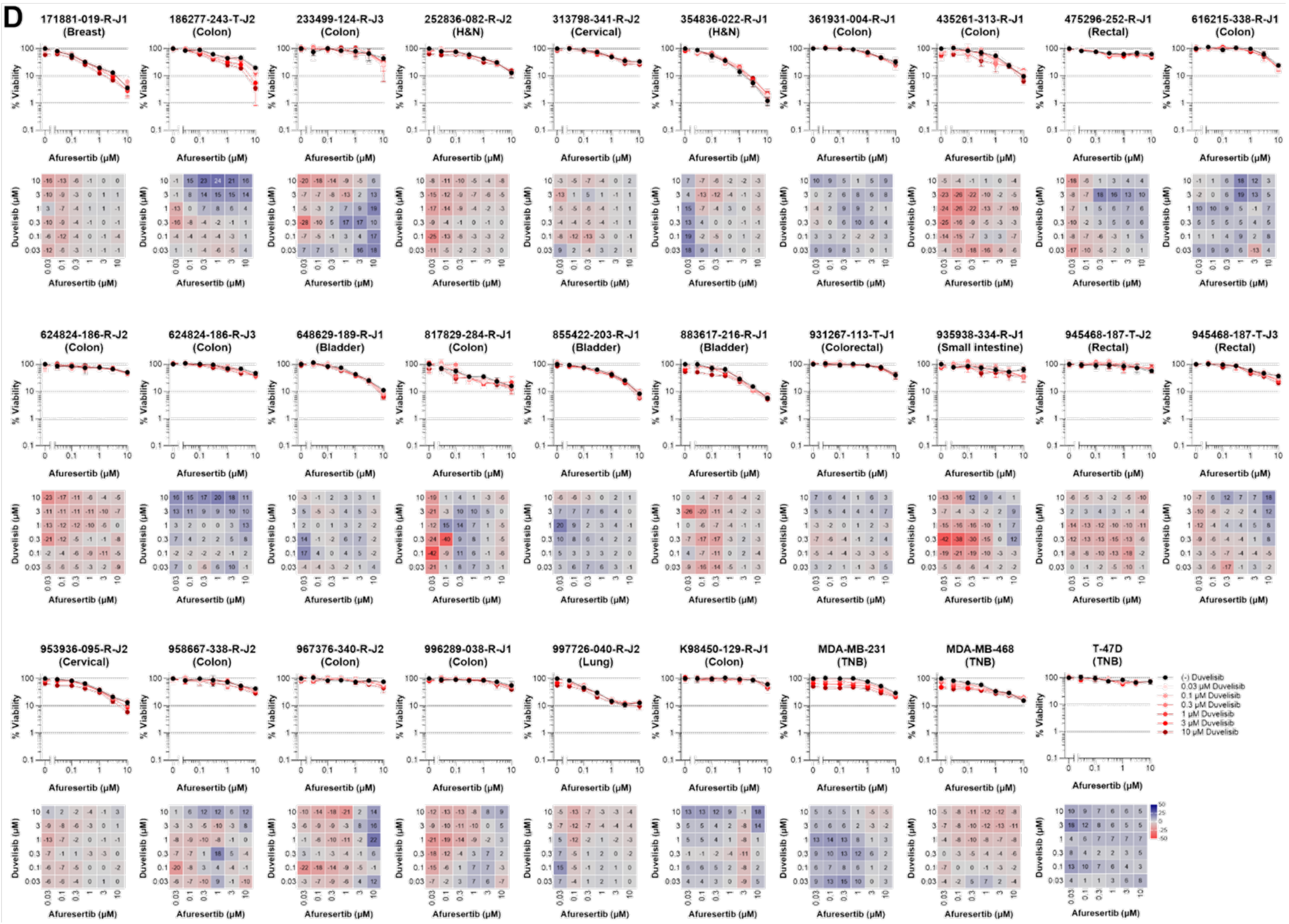
D
171881-019-R-J1 (Breast)
186277-243-T-J2 (Colon)
233499-124-R-J3 (Colon)
252836-082-R-J2 (H&N)
313798-341-R-J2 (Cervical)
354836-022-R-J1 (H&N)
361931-004-R-J1 (Colon)
435261-313-R-J1 (Colon)
475296-252-R-J1 (Rectal)
616215-338-R-J1 (Colon)
624824-186-R-J2 (Colon)
624824-186-R-J3 (Colon)
648629-189-R-J1 (Bladder)
817829-284-R-J1 (Colon)
855422-203-R-J1 (Bladder)
883617-216-R-J1 (Bladder)
931267-113-T-J1 (Colorectal)
935938-334-R-J1 (Small intestine)
945468-187-T-J2 (Rectal)
945468-187-T-J3 (Rectal)
953936-095-R-J2 (Cervical)
958667-338-R-J2 (Colon)
967376-340-R-J2 (Colon)
996289-038-R-J1 (Colon)
997726-040-R-J2 (Lung)
K98450-129-R-J1 (Colon)
MDA-MB-231 (TNB)
MDA-MB-468 (TNB)
T-47D (TNB)
% Viability
Afuresertib (μM)
Duvelisib (μM)
(-) Duvelisib
0.03 μM Duvelisib
0.1 μM Duvelisib
0.3 μM Duvelisib
1 μM Duvelisib
3 μM Duvelisib
10 μM Duvelisib

Supplemental Figure S9. Combination activity from afuresertib with a PI3K inhibitor. Concentration-response graphs (*top*, mean ± SD, $n = 3$ technical replicates) and Bliss independence scores across the concentration matrix (*bottom*, mean of $n = 3$ technical replicates) are shown as numerical values and heat maps (blue indicates synergy, gray indicates additivity, and red indicates antagonism). Data are presented, if available, from twenty-nine malignant cell lines grown as multi-cell type tumor spheroids and treated with afuresertib in combination with (A) alpelisib, (B) inavolisib, (C) copanlisib, or (D) duvelisib. The tumor model name and type are indicated above each set of graphs.

Supplemental Table S5

Name	Group	REGID	AID	Link to access data
Anticancer human tumor 171881-019-R-J1-PDC cell line growth inhibition [PI3K]	Combinations_Synergy_Screen_PI3K	CSSPI3K_01	1963986	https://pubchem.ncbi.nlm.nih.gov/bioassay/1963986
Anticancer human tumor 186277-243-T-J2-PDC cell line growth inhibition [PI3K]	Combinations_Synergy_Screen_PI3K	CSSPI3K_02	1963983	https://pubchem.ncbi.nlm.nih.gov/bioassay/1963983
Anticancer human tumor 233499-124-R-J3-PDC cell line growth inhibition [PI3K]	Combinations_Synergy_Screen_PI3K	CSSPI3K_03	1963984	https://pubchem.ncbi.nlm.nih.gov/bioassay/1963984
Anticancer human tumor 252836-082-R-J2-PDC cell line growth inhibition [PI3K]	Combinations_Synergy_Screen_PI3K	CSSPI3K_04	1963985	https://pubchem.ncbi.nlm.nih.gov/bioassay/1963985
Anticancer human tumor 313798-341-R-J2-PDC cell line growth inhibition [PI3K]	Combinations_Synergy_Screen_PI3K	CSSPI3K_05	1963982	https://pubchem.ncbi.nlm.nih.gov/bioassay/1963982
Anticancer human tumor 354836-022-R-J1-PDC cell line growth inhibition [PI3K]	Combinations_Synergy_Screen_PI3K	CSSPI3K_06	1963981	https://pubchem.ncbi.nlm.nih.gov/bioassay/1963981
Anticancer human tumor 361931-004-R-J1-PDC cell line growth inhibition [PI3K]	Combinations_Synergy_Screen_PI3K	CSSPI3K_07	1963979	https://pubchem.ncbi.nlm.nih.gov/bioassay/1963979
Anticancer human tumor 435261-313-R-J1-PDC cell line growth inhibition [PI3K]	Combinations_Synergy_Screen_PI3K	CSSPI3K_08	1963976	https://pubchem.ncbi.nlm.nih.gov/bioassay/1963976
Anticancer human tumor 475296-252-R-J1-PDC cell line growth inhibition [PI3K]	Combinations_Synergy_Screen_PI3K	CSSPI3K_09	1963980	https://pubchem.ncbi.nlm.nih.gov/bioassay/1963980
Anticancer human tumor 616215-338-R-J1-PDC cell line growth inhibition [PI3K]	Combinations_Synergy_Screen_PI3K	CSSPI3K_10	1963978	https://pubchem.ncbi.nlm.nih.gov/bioassay/1963978
Anticancer human tumor 624824-186-R-J2-PDC cell line growth inhibition [PI3K]	Combinations_Synergy_Screen_PI3K	CSSPI3K_11	1963975	https://pubchem.ncbi.nlm.nih.gov/bioassay/1963975
Anticancer human tumor 624824-186-R-J3-PDC cell line growth inhibition [PI3K]	Combinations_Synergy_Screen_PI3K	CSSPI3K_12	1963977	https://pubchem.ncbi.nlm.nih.gov/bioassay/1963977
Anticancer human tumor 648629-189-R-J1-PDC cell line growth inhibition [PI3K]	Combinations_Synergy_Screen_PI3K	CSSPI3K_13	1963974	https://pubchem.ncbi.nlm.nih.gov/bioassay/1963974
Anticancer human tumor 817829-284-R-J1-PDC cell line growth inhibition [PI3K]	Combinations_Synergy_Screen_PI3K	CSSPI3K_14	1963969	https://pubchem.ncbi.nlm.nih.gov/bioassay/1963969
Anticancer human tumor 855422-203-R-J1-PDC cell line growth inhibition [PI3K]	Combinations_Synergy_Screen_PI3K	CSSPI3K_15	1963967	https://pubchem.ncbi.nlm.nih.gov/bioassay/1963967
Anticancer human tumor 883617-216-R-J1-PDC cell line growth inhibition [PI3K]	Combinations_Synergy_Screen_PI3K	CSSPI3K_16	1963973	https://pubchem.ncbi.nlm.nih.gov/bioassay/1963973
Anticancer human tumor 931267-113-T-J1-PDC cell line growth inhibition [PI3K]	Combinations_Synergy_Screen_PI3K	CSSPI3K_17	1963970	https://pubchem.ncbi.nlm.nih.gov/bioassay/1963970
Anticancer human tumor 935938-334-R-J1-PDC cell line growth inhibition [PI3K]	Combinations_Synergy_Screen_PI3K	CSSPI3K_18	1963966	https://pubchem.ncbi.nlm.nih.gov/bioassay/1963966
Anticancer human tumor 945468-187-T-J2-PDC cell line growth inhibition [PI3K]	Combinations_Synergy_Screen_PI3K	CSSPI3K_19	1963972	https://pubchem.ncbi.nlm.nih.gov/bioassay/1963972
Anticancer human tumor 945468-187-T-J3-PDC cell line growth inhibition [PI3K]	Combinations_Synergy_Screen_PI3K	CSSPI3K_20	1963968	https://pubchem.ncbi.nlm.nih.gov/bioassay/1963968
Anticancer human tumor 953936-095-R-J2-PDC cell line growth inhibition [PI3K]	Combinations_Synergy_Screen_PI3K	CSSPI3K_21	1963971	https://pubchem.ncbi.nlm.nih.gov/bioassay/1963971
Anticancer human tumor 958667-338-R-J2-PDC cell line growth inhibition [PI3K]	Combinations_Synergy_Screen_PI3K	CSSPI3K_22	1963963	https://pubchem.ncbi.nlm.nih.gov/bioassay/1963963
Anticancer human tumor 967376-340-R-J2-PDC cell line growth inhibition [PI3K]	Combinations_Synergy_Screen_PI3K	CSSPI3K_23	1963962	https://pubchem.ncbi.nlm.nih.gov/bioassay/1963962
Anticancer human tumor 996289-038-R-J1-PDC cell line growth inhibition [PI3K]	Combinations_Synergy_Screen_PI3K	CSSPI3K_24	1963965	https://pubchem.ncbi.nlm.nih.gov/bioassay/1963965
Anticancer human tumor 997726-040-R-J2-PDC cell line growth inhibition [PI3K]	Combinations_Synergy_Screen_PI3K	CSSPI3K_25	1963964	https://pubchem.ncbi.nlm.nih.gov/bioassay/1963964
Anticancer human tumor K98450-129-R-PDC cell line growth inhibition [PI3K]	Combinations_Synergy_Screen_PI3K	CSSPI3K_26	1963961	https://pubchem.ncbi.nlm.nih.gov/bioassay/1963961
Anticancer human tumor MDA-MB-231 cell line growth inhibition [PI3K]	Combinations_Synergy_Screen_PI3K	CSSPI3K_27	1963958	https://pubchem.ncbi.nlm.nih.gov/bioassay/1963958
Anticancer human tumor MDA-MB-468 cell line growth inhibition [PI3K]	Combinations_Synergy_Screen_PI3K	CSSPI3K_28	1963960	https://pubchem.ncbi.nlm.nih.gov/bioassay/1963960
Anticancer human tumor T-47D cell line growth inhibition [PI3K]	Combinations_Synergy_Screen_PI3K	CSSPI3K_29	1963959	https://pubchem.ncbi.nlm.nih.gov/bioassay/1963959
